# Estimated frequency and economic burden of incident fragility fractures during 2023 in Mexico

**DOI:** 10.1007/s11657-024-01468-2

**Published:** 2024-11-07

**Authors:** Fernando Carlos-Rivera, Jorge Antonio Guzmán-Caniupan, Luis Miguel Camacho-Cordero, Therese Aubry de Maraumont, Noe Soria-Suárez

**Affiliations:** 1 Pharmacoeconomics Department, AHS Health Consulting, S.A.S. de C.V., Huixquilucan, State of Mexico Mexico; 2 Access Department, AHS Health Consulting, S.A.S. de C.V., Huixquilucan, State of Mexico Mexico; 3 Health Economics Department, Mexico City, Amgen Mexico Mexico; 4 Head Value & Access Department, Mexico City, Amgen Mexico Mexico; 5 Head Therapeutic Area Bone, Mexico City, Amgen Mexico Mexico

**Keywords:** Osteoporosis, Bone fractures, Epidemiology, Cost of illness, Healthcare costs, Mexico

## Abstract

***Summary*:**

Epidemiologic and economic data regarding osteoporotic fractures in Mexico is scarce and mostly outdated. Through a model, we estimated the incidence and costs of osteoporotic fractures in adults ≥ 50 years old in Mexico during the year 2023. Results showed that these events are both frequent and costly, leading to a considerable economic impact.

**Purpose:**

Osteoporosis and its fractures impose a high clinical and economic burden. The objective of this analysis was to estimate the frequency and costs owing to incident fragility fractures (FFs) during the year 2023 in Mexico.

**Methods:**

This is an incidence-based cost-of-illness study. The target population is adults ≥ 50 years old sustaining a fracture related to osteoporosis (caused by a fall on the same level). The model estimates the costs and productivity losses associated with their treatment within 1 year post-fracture. National epidemiologic databases supplemented with information, derived from literature when appropriate, were used to estimate the frequency of new FFs during 2023 in the study population. Resource use included surgical and non-surgical inpatient or ambulatory care the patients received immediately after fracture plus the outpatient physiotherapy post-discharge and the eventual follow-up with a specialist who may prescribe pharmacotherapy. Sick days taken in employed patients were estimated from the literature. Local unitary costs of services and drugs for both public and private settings as well as average income in those occupied were applied. All costs are reported in Mexican pesos (MXN) from 2023.

**Results:**

The model estimated a total of 229,239 FFs, among which 63% were classified as a major osteoporotic fracture, including 53,842 and 41,459 fractures located at the hip and vertebral, respectively. The total costs were estimated at 15,593 million MXN; most of them (75.2%) were attributable to acute-phase care.

**Conclusions:**

Fragility fractures represent a serious health problem for Mexico. Better preventive/therapeutic strategies may help to mitigate their significant financial toll.

**Supplementary Information:**

The online version contains supplementary material available at 10.1007/s11657-024-01468-2.

## Introduction

Osteoporosis (OP) is a disease of the skeleton that weakens the bones and increases the risk of fragility fractures (FFs, also known as osteoporotic fractures), where bones can break from low-level impact or stress that would not normally break a healthy bone [1, 2]. A comprehensive systematic review and meta-analysis estimated the global prevalence of OP in the world at 18.3% (95% confidence interval, 95% CI 16.2–20.7), with higher values in women than in men: 23.1% (95% CI 19.8–26.9) vs. 11.7% (95% CI 9.6–14.1) [3]. The prevalence of OP in adults aged 50 years and older in Mexico has been reported to be 16–17% in women and 6–7% in men [4].

The clinical relevance of OP lies in the associated FFs; until such an event occurs, there are usually no symptoms. In the Western World, about 1 in 3 women and 1 in 5 men above 50 years of age will fracture during their remaining lifetime [2]. After the age of 50 years, most sites of fracture can be considered characteristic of OP [2]. FFs are associated with serious clinical consequences, including chronic pain, skeletal deformities, loss of independence, and increased mortality [5]. The incidence of FF increases markedly with age, though the rate of rise with age differs for different fracture outcomes. For this reason, the proportion of fractures at any site also varies with age [6]. A prior fracture increases the risk of subsequent fractures [7].

OP and its fractures not only have a major impact on health and loss of independence but also impose a significant economic burden on the health system, with FF-related costs being the main cost driver [8]. The total cost of (incident and prevalent) FFs during 2019 in Europe amounted to €56.9 billion [6]. A study predicts the annual fractures in US women to increase from 1.9 million to 3.2 million (68%), from 2018 to 2040, with related costs rising from 57 billion to over 95 billion US dollars (USD) [9]. The total cost of incident FFs in four Latin American countries (Brazil, Mexico, Colombia, and Argentina) in 2018 amounted to 1.17 billion USD [7].

Data regarding the economic burden of OP and its fractures is scarce in Mexico. A previous study [10] could be somehow outdated since the projections made by Carlos et al. and the sources for costing the FFs are based on data from the year 2010. Also, Carlos et al. excluded some cost items, such as those related to the diagnosis and pharmacological management of OP and the indirect costs [10]. On the other hand, a 2019 study led by Aziziyeh et al. [7] carries some limitations, especially in the number of assumptions used as well as in some of the sources considered when they estimated the average costs per type of FF. Aziziyeh et al. [7] included indirect costs resulting from patient productivity loss, but they excluded follow-up costs.

## Purpose

To characterize the contemporary epidemiology of incident FFs in men and women over 50 years in Mexico in order to estimate the frequency and costs owing to the total number of new cases of FF in this population during the year 2023.

## Methods

### Study design and population

This is a cost-of-illness study that follows an incidence approach. An incidence-based cost-of-illness involves estimating the cost of the new cases of a condition or group of conditions which have their onset in a given period of time [11]. Specifically, the study focuses on the frequency of new FFs estimated in the target study population and their associated economic burden. So the broad study population comprises men and women were at least 50 years of age during 2023 in our country. Briefly, we first estimated the FF incidence rates by age and sex in Mexico during 2019 based on epidemiologic national data supplemented with literature, and then, we applied those rates to the demographic structure projected for 2023 in Mexico. Once the number of new FFs in the target population was obtained, we estimated the total costs due to these FFs in the first year post-fracture through an economic model.

### Time horizon

The individuals among the study population estimated to sustain a FF are followed through an economic model designed in Microsoft Excel® to determine the care pathway for a maximum of 12 months after they are discharged from the (initial) acute care received in the emergency room, the inpatient setting, or after an office visit (the latter only applies for vertebral fractures).

### Epidemiologic data sources

#### Definitions

The primary epidemiologic outcomes in this study were the new cases of FF [2, 12] diagnosed either in the inpatient or in the outpatient setting. The operational definition of FF occurred in the inpatient setting corresponds to the hospital discharges of interest. The outpatient setting refers to the cases where the patients received (initial) acute care after sustaining the fracture in the emergency room only (i.e., without being hospitalized) as well as to those individuals with clinical vertebral FF diagnosed as part of an ambulatory office visit to a specialist. The names and descriptions of the fractures evaluated in the present analysis are shown in Table 1. Briefly, there are two main categories: major osteoporotic fracture (MOF), defined as a grouping of the most common fractures comprising hip, spine (clinical vertebral and some major fractures of the lumbar spine and pelvis), proximal humerus, and distal forearm fractures, and non-MOF, a term that refers to “other” osteoporotic fractures that are not MOFs unless specifically defined [2]. It is important to remark that vertebral fractures relevant to the present investigation are those coming to seek medical attention rather than the broader subclass of morphometric fractures (asymptomatic, subclinical) [2, 12]. Furthermore, the proximal humerus fracture subcategory in this study includes those fractures located at the upper epiphysis as well as those located at the diaphysis (shaft) of the humerus.

**Table 1:** Categories of fragility fractures according to ICD-10 codes involved in its definition

Category	ICD-10 codes
Major osteoporotic fracture	
Hip	S72.0, S72.1, and S72.2
Vertebral	S12.0, S12.1, S12.2, S12.7, S22.0, S22.1, S32.0, and M48.5
Proximal humerus	S42.2 and S42.3
Distal forearm	S52.5, S52.6, and S52.7
Lumbar spine, pelvis	S32.7 and S32.8
Non-major osteoporotic fracture	
Thorax, lumbar zone, pelvis	S22.2, S22.3, S22.4, S22.5, S32.1, S32.2, S32.3, S32.4, and S32.5
Shoulder, upper arm	S42.0, S42.1, S42.4, S42.7, S42.8, and S42.9
Proximal forearm	S52.0, S52.1, S52.2, S52.3, S52.4, S52.8, and S52.9
Other femoral	S72.3, S72.4, S72.7, S72.8, and S72.9
Tibia and fibula^a^	S82.1, S82.2, S82.3, and S82.4

Source: own elaboration based on Borgström et al. [2]

*ICD-10* International Statistical Classification of Diseases and Related Health Problems, 10th revision

^a^Women only

In recent years, the term “osteoporotic fracture” has been interchangeably used with terms such as “fragility fracture,” “low-trauma fracture,” “low-level fracture,” “low-energy fracture,” or “low-impact fracture,” denoting that fractures would not ordinarily result from low mechanical forces. In general, such low mechanical forces have been quantified as equivalent to a fall from a standing height or less [13]. Hence, the operational definition for FF used in this study corresponds to those fractures caused by a fall on the same level (International Statistical Classification of Diseases and Related Health Problems, 10th revision [ICD-10] codes: W00–W03, W05–W08, and W18) [14]. Fractures of the categories of interest with unspecified falls (ICD-10 code W19) were proportionally distributed between “fall on the same level” and “fall from one level to another” according to their relative frequency based on the number of fractures with a specified type of fall.

#### Adults ≥ 50 years old in Mexico

The 2023 mid-year sex- and age-specific population of adults ≥ 50 years old in Mexico projected by the National Population Council (CONAPO) [15] is shown in Table 2. These numbers represent the individuals at risk of sustaining FF in terms of the present analysis, reaching around 29.8 million. Women represent a slight majority (53.6%) of the total population study. The population in each combination of sex and age group was multiplied for the incidence rates of FF (see below) to obtain the projected number of FFs according to sex and age group in Mexico for the year 2023.

**Table 2:** Epidemiologic data

Parameter/age group	50 to 59	60 to 69	70 to 79	80 to 89	90 +
Population, year 2023					
Men	6,471,202	4,292,776	2,135,982	788,978	154,288
Women	7,198,787	4,878,712	2,589,991	1,059,875	232,550
MOF, incidence per 100,000 men					
Hip	16	52	214	684	1,431
Vertebral^a^	51	70	186	304	318
Proximal humerus	15	26	53	58	155
Distal forearm	25	27	29	47	49
Lumbar spine, pelvis	1	2	4	13	22
MOF, incidence per 100,000 women					
Hip	22	115	459	1,361	2,104
Vertebral^a^	64	166	382	456	353
Proximal humerus	28	92	185	219	269
Distal forearm	108	167	239	287	212
Lumbar spine, pelvis	1	4	10	33	49
Non-MOF, incidence per 100,000 men					
Thorax, lumbar zone, pelvis	11	20	31	51	67
Shoulder, upper arm	21	24	31	50	57
Proximal forearm	39	50	61	120	137
Other femoral	13	22	82	207	338
Tibia and fibula	NA	NA	NA	NA	NA
Non-MOF, incidence per 100,000 women					
Thorax, lumbar zone, pelvis	8	15	54	108	86
Shoulder, upper arm	17	43	77	132	138
Proximal forearm	116	176	266	298	293
Other femoral	17	59	154	441	642
Tibia and fibula	81	82	72	95	66

Source: own estimates based on data extracted from references [12, 13], and [15–18]

*MOF* major osteoporotic fractures, *NA* not applicable

^a^Calculated by multiplying the rate of hip fracture to the sex and age-specific factor of vertebral to hip fractures incidence reported in Sweden[12]

#### Public healthcare sector

The eight public institutions evaluated were the Mexican Social Security Institute (IMSS), the Institute of Security and Social Services of State Workers (ISSSTE), the Secretariat of Health (SSA), the Mexican state-owned petroleum company (PEMEX), the IMSS-Bienestar program, the health services in the 32 states or provinces in which is divided the country, the Secretariat of National Defense (SEDENA), and the Secretariat of the Navy (SEMAR).

#### Incidence rates of fragility fractures

The model considers data from the year 2019 because the more recent years (2020 to 2022) are atypical due to the COVID-19 pandemic. The process for estimating the incidence rates of FF in each category of interest was as follows. First, the sex- and age group–specific all-causes (i.e., osteoporotic and non-osteoporotic) fractures in the inpatient setting were identified for six types of public institution (SSA, IMSS, ISSSTE, PEMEX, IMSS-Bienestar, and health state services (Supplementary Table 1 for men and Supplementary Table 2 for women)). The data was gathered from the Discharges Dynamic Cubes of the General Direction of Health Information (DGIS for its Spanish acronym) in the SSA [16], which provides specific information by public institutions in a standardized format. Given that the DGIS did not have data related to hospital discharges in the year 2019 reported for SEMAR and SEDENA, we estimated their number of fractures in the inpatient setting indirectly. Thus, the number of fractures in the inpatient setting for the SEMAR was estimated from the relation in the frequency of hospital discharges due to fractures between that institution and the SSA in the year 2021 [16] (Supplementary Table 3). The number of fractures in the inpatient setting for the SEDENA was assumed to be directly proportional to the ratio of beneficiaries of SEDENA to SEMAR in the year 2019 (a factor of 3.82, considering their average number of beneficiaries between December 2018 and December 2019) [17]. Then, the hospital discharges in the eight public institutions were aggregated. The number of fractures in the inpatient setting for the private sector was calculated by multiplying the cumulative frequencies of fracture in IMSS, ISSSTE, SEDENA, and PEMEX by a factor of 0.35 that was derived from the relation of overall (all-causes, not only fractures) hospital discharges between the private hospital and clinics [18] and these public institutions [16] (1,942,738 vs. 5,556,264 overall hospital discharges, respectively, in the year 2019). Second, the ratio of fractures in emergency room vs. hospital discharges for each category of FF of interest in the SSA was calculated from the Emergencies and Discharges Dynamic Cubes of the DGIS [16], and the obtained ratios (Supplementary Table 4) were applied to the number of hospital discharges due to the fractures in both the public and private sectors to determine the number of cases of fractures treated in the emergency room only in each sector. To avoid double-counting, the cases where patients received initial care in the emergency room and then transferred to a hospital as part of the acute care were excluded. Third, these aggregated numbers (i.e., the sum of fractures in the inpatient setting and the fractures that received initial care at the emergency room only across each sector) were filtered by the corresponding percentage of all fractures that are caused by a fall on the same level (Supplementary Table 5). For most categories of fracture, these percentages were estimated with data extracted for the year 2019 from the Injuries Dynamic Cube of the DGIS in the SSA [16], following the criteria explained previously in the subsection named definitions. In the categories of vertebral, other major fractures at the lumbar spine or pelvis, and non-MOF located at the thorax, lumbar zone, and pelvis, the local calculated values were considered as low plausible and then substituted by the best-matched values published by Muschitz et al. [13]. In the absence of specific data for the private healthcare sector, the values of Supplementary Table 5 were applied to both the public and private sectors.

The sex- and age group–specific FFs estimated for the year 2019 broken down by type of initial care received and sector are shown in Supplementary Table 6 (men) and Supplementary Table 7 (women). Given that clinical vertebral fractures are not commonly diagnosed either as a hospital discharge or as a case attended in an emergency room but as an ambulatory setting through a consultation visit to a specialist, we included the category specialty visit only as an additional term of initial care for this type of fracture. The sex and age group number of total vertebral FFs were calculated by multiplying the corresponding sex and age group number of total hip FFs by an adjustment factor. The adjustment factors (Supplementary Table 8) were based on the ratio of vertebral to hip fracture by sex- and age group–specific incidence reported in Sweden [12]. Therefore, the number of vertebral FFs considered as a specialty visit only was calculated as the total minus the sum of hospital discharges and those cases treated as an emergency room visit only.

In the end, sex- and age group–specific fractures classified as FF (Supplementary Tables 6 and 7) were divided by the 2019 mid-year sex- and age group–specific population in Mexico (Supplementary Table 9) giving, as a result, the estimated incidence rates of FF in the year 2019 (Table 2). The sex- and age group–specific FFs by type in the year 2023 were obtained by combining the mid-year population data (Table 2) with those rates. The proportion of FFs requiring hospitalization as part of the acute care is displayed in Table 3. The numbers were derived from Supplementary Table 6 (men) and Supplementary Table 7 (women). The proportion of vertebral FFs receiving a specialty visit only as acute care is also based on those sources. Based on the data from the year 2019, it was considered that 74.5% of all FFs in 2023 were treated in the public sector.

**Table 3:** Proportion of fragility fractures requiring hospitalization as part of the acute care

Parameter/age group	50 to 59	60 to 69	70 to 79	80 to 89	90 +
Data for men					
Hip	68.3%	63.8%	70.4%	67.7%	66.2%
Vertebral	3.7%	5.4%	2.7%	1.9%	1.1%
Proximal humerus	50.6%	40.1%	38.9%	41.2%	32.4%
Distal forearm	39.6%	31.2%	34.8%	24.6%	10.6%
Lumbar spine, pelvis	54.2%	51.2%	44.1%	34.0%	33.3%
Thorax, lumbar zone, pelvis	21.2%	25.9%	25.6%	24.4%	21.7%
Shoulder, upper arm	29.4%	26.6%	22.8%	22.0%	21.1%
Forearm	39.5%	35.1%	30.2%	27.6%	14.0%
Other femoral	65.6%	68.1%	56.6%	57.4%	64.4%
Tibia and fibula	NA	NA	NA	NA	NA
Data for women					
Hip	63.4%	63.1%	66.0%	69.1%	64.3%
Vertebral	3.6%	2.9%	2.2%	1.6%	1.2%
Proximal humerus	41.8%	35.4%	33.5%	34.8%	23.6%
Distal forearm	24.3%	27.7%	25.7%	27.1%	24.4%
Lumbar spine, pelvis	48.4%	25.8%	36.9%	27.7%	19.5%
Thorax, lumbar zone, pelvis	13.6%	16.5%	10.1%	11.0%	21.6%
Shoulder, upper arm	27.0%	24.7%	23.3%	19.0%	19.7%
Forearm	29.3%	31.2%	29.3%	28.4%	23.1%
Other femoral	61.7%	65.0%	62.2%	61.0%	57.5%
Tibia and fibula	38.6%	42.7%	42.6%	49.4%	43.7%

Source: own estimates based on data extracted from references [12, 13], and [16–18] NA not applicable

### Economic data sources

All costs are expressed in Mexican pesos for the year 2023 (exchange rate as on November 17, 2023, 17.2708 and 18.7077 Mexican pesos for 1 USD and 1 euro, respectively). The main economic parameters are shown in Table 4 (See Supplementary Table 10 for the corresponding values in USD). In the analysis, total costs are divided into direct medical and indirect costs. Direct medical costs include acute care costs and follow-up costs. Acute care costs comprise either the cost of an emergency visit or a weighted cost if the patient is hospitalized (Supplementary Table 11). These weighted costs reflect the fact that some patients will have a surgical procedure while others will not (i.e., conservative treatment). For the hip and vertebral, the weighted cost also considers the different types of surgery (e.g., partial/total hip replacement vs. open/closed reduction with/without internal fixation) that can be performed. Individuals with clinical vertebral fractures diagnosed in outpatient care were assigned the costs of two specialty visits plus radiography as an acute care cost category. Follow-up costs for 1 year include the costs of rehabilitation (Supplementary Table 12), specialty visits (Supplementary Table 13), laboratory (Supplementary Table 14 for the public sector and Supplementary Table 15 for the private sector) and imaging (Supplementary Table 16 for the public sector and Supplementary Table 17 for the private sector) tests, and drugs (pharmacological treatment and supplementation) given in the outpatient setting after the acute care phase (Supplementary Table 18 for the public sector and Supplementary Table 19 for the private sector). Indirect costs refer to the productivity losses due to the fracture.

**Table 4:** Cost per event of fragility fracture by category: Mexican pesos

Type of fragility fracture	Direct medical costs: inpatient acute phase plus outpatient care given during the follow-up period^a^						Indirect costs
	Acute phase^b^	Rehabilitation	Specialty visits	Laboratory tests	Imaging tests	Drugs^c^	Productivity losses
Hip	$117,933	$3400	$2786	$3225	$1518	$1315	$3775
	$333,751	$6531	$4130	$19,260	$4074	$5605	
Vertebral	$62,546	$2707	$2248	$2916	$1567	$546	$2123
	$177,006	$5250	$3226	$18,732	$4004	$2256	
Proximal humerus	$61,863	$3400	$1233	$2433	$917	$546	$1143
	$175,073	$6234	$1615	$15,231	$1717	$2256	
Distal forearm	$61,970	$3117	$1318	$2564	$960	$92	$1222
	$175,374	$6070	$1675	$15,585	$2255	$379	
Lumbar spine, pelvis	$74,803	$3683	$2521	$2934	$1604	$546	$1158
	$211,692	$6070	$2720	$16,900	$3225	$2256	
Thorax, lumbar zone, pelvis	$95,765	$1148	$1011	$2303	$954	$546	$1365
	$271,014	$1406	$1251	$14,260	$1822	$2256	
Shoulder, upper arm	$60,161	$3400	$1233	$2433	$917	$546	$1354
	$170,255	$6234	$1615	$15,231	$1717	$2256	
Proximal forearm	$61,970	$3117	$1318	$2564	$960	$92	$1291
	$175,374	$6070	$1675	$15,585	$2255	$379	
Other femoral	$95,937	$3117	$1318	$2564	$960	$546	$1141
	$271,501	$6070	$1675	$15,585	$2255	$2256	
Tibia and fibula	$72,103	$3758	$1348	$2337	$905	$546	$1227
	$204,052	$5469	$1861	$14,057	$1557	$2256	

Source: own estimates. See Supplementary Tables 11 to 20 to more details

^a^Each type of fracture has two rows: the upper value is the cost at the public sector, whereas the lower value corresponds to the cost at the private sector. Productivity losses are the same for both sectors

^b^For individuals who were hospitalized. Patients treated only at the emergency room or with a vertebral fracture diagnosed at the outpatient level without any inpatient care entail different costs (see text)

^c^Includes both pharmacological treatment and supplementation with calcium plus vitamin D

The resource use in both the public and the private sector was mainly derived from an expert panel with local key opinion leaders. The estimates for the public sector consider the costs of surgical/medical diagnosis-related groups (DRG; see Supplementary Table 11 for specific codes) and the unit cost of a specialty visit and a session of rehabilitation requested to the IMSS [19] as well as the unit cost of an emergency visit and some laboratory and imaging tests reported in the catalog for services interchanges among the IMSS, SSA, ISSSTE and PEMEX [20]. The unit cost of other laboratories in the public sector was defined according to its recovery fee in the sixth socioeconomic level at the National Nutrition Institute [21]. The estimates for the private sector were performed with information gathered mainly through phone calls to a private medical center located in Mexico City. The acute care for patients hospitalized in the private sector was calculated by applying an adjustment fixed factor of 2.83 (data rounded to only two decimals) to their corresponding public cost values. This factor reflects the relative cost difference for an arthroplasty in the private medical center used as reference ($372,450 Mexican pesos; 21,565 USD) to the DRG cost in the IMSS ($131,496 Mexican pesos; $7614 USD; see Supplementary Table 11). The proportion of patients receiving pharmacological treatment or supplementation with calcium and vitamin D after a hip, vertebral, and forearm FFs was gathered from Macías-Hernández et al. [22]. It was assumed that the rest of the FFs have the same percentages of these therapies as vertebral fractures. The proportional distribution among pharmacological therapy (alendronate, risedronate, zoledronic acid, denosumab, and teriparatide) in both sectors (public and private) was defined according to their estimated market share in the public sector obtained as part of this study. The prices of the pharmacological treatment products and supplementation in the public sector were obtained from the Annual Program of Acquisitions, Leases, Services and Public Works (PAAASOP) [23]. This source was also used to estimate the market share. For the private sector, these prices were obtained from two online pharmacies (Farmacia del Ahorro and Farmalisto). The unit cost per intravenous infusion of zoledronic acid in the public sector corresponds to the value derived from the list of services at the IMSS [19]; in the private sector, that cost was obtained through a phone call to one provider of the service located in the Monterrey City (Biológicos Especializados). Supplementary Tables 18 and 19 present detailed information regarding the costing estimates of drugs.

The productivity losses due to FF (Supplementary Table 20) were estimated using the human capital approach considering four relevant parameters. First, the proportional distribution by sex and age group (50–59 and 60 + years) among those with the type of FF of interest, estimated for year 2023 in Mexico, was derived from Supplementary Table 21 for men and Supplementary Table 22 for women. Second, the values obtained were combined with the sex and age group population economically active based on data produced by the CONAPO [15] and the National Occupation and Employment Survey (ENOE) [24]. The sum product of these values gives an overall percentage of occupied population for those patients 50 + years by type of FF (for example, 33.93% in the case of hip fractures). The third relevant parameter was the average daily income for the target population, derived from the ENOE ($264.89 Mexican pesos; $15.34 USD) [24]. The fourth and last parameter was the number of sick days per type of FF, as reported by Borgström et al. [2]. The indirect cost per type of fracture was calculated as the product of these three factors: (i) the percentage of patients sustaining a FF that were economically active, (ii) the average daily income for the target population, and (iii) the number of sick days. Using hip fracture as an example, the indirect costs were equal to 0.3393 × 264.89 × 42 = $3775 Mexican pesos (Supplementary Table 20).

## Results

Table 5 presents the total estimated new cases of FF in 2023 by type of fracture and category of initial care received. Of 144,367 total MOFs, hip fracture was 53,842 (37.3%), vertebral fracture was 41,459 (28.7%), and others were 49,066 (34.0%). Around 36.2% of them (52,234) corresponded to fractures requiring inpatient initial care. The model also estimated a total of 84,872 non-MOFs, with forearm (34,783) and other femoral fractures (19,932) being the most frequent sites. Approximately 37.6% of the non-MOFs were treated in the inpatient setting as part of their acute care. Adding MOFs and non-MOFs yielded 229,239 total fractures. The MOFs represented the majority among the two big categories, representing 63% of the grand total, with 84,178 (36.7%) of them being classified as hospital discharges during the acute care phase. Further details are available in Supplementary Table 21 (men), Supplementary Table 22 (women) and Supplementary Table 23 (all).

**Table 5:** Projected number of fragility fractures per category: Mexico 2023

Category	Frequency			% Distribution vs. grand total
	Hospital discharge^a^	Emergency room visit^b^	Total	
Major osteoporotic fracture	52,234	52,835	144,367	63.0%
Hip	35,968	17,874	53,842	23.5%
Vertebral	1144	1017	41,459^c^	18.1%
Proximal humerus	6652	11,552	18,204	7.9%
Distal forearm	8001	21,492	29,493	12.9%
Lumbar spine, pelvis	469	900	1369	0.6%
Non-major osteoporotic fracture	31,944	52,928	84,872	37.0%
Thorax, lumbar zone, pelvis	1174	5600	6774	3.0%
Shoulder, upper arm	2566	7979	10,545	4.6%
Proximal forearm	10,624	24,159	34,783	15.2%
Other femoral	12,268	7664	19,932	8.7%
Tibia and fibula	5311	7527	12,838	5.6%
Fragility fractures, grand total	84,178	105,763	229,239	100.0%

See Supplementary Tables 21 to 23 for further details

^a^Number of fragility fractures diagnosed as a hospital discharge because the patient needed (surgical or non-surgical) inpatient care immediately after sustained the fracture

^b^Number of fragility fractures diagnosed as an only emergency room visit because the patient was not hospitalized after sustained the fracture. It excludes cases where the patient was initially received in an emergency service and then transferred to a hospital for inpatient care

^c^Include 39,298 patients estimated to be diagnosed with a clinical vertebral fracture in the ambulatory setting after a visit to a specialist (e.g., rheumatologist, endocrinologist, gynecologist, geriatrist)

Tables 6 and 7 display the projected economic burden (see Supplementary Table 24 for estimates in USD). In Table 6, the disaggregated costs are presented for the main FF categories of interest. As can be observed, the costs during acute care represent the main driver in all types of fractures except vertebral fractures. The total costs owing MOFs were calculated at 10,655 million Mexican pesos (almost 617 million USD), with hip fractures being responsible for 70.3% of that figure. The total costs in the non-MOF category were 4938 million Mexican pesos (almost 286 million USD). The grand total costs reached 15,593 million Mexican pesos (almost 903 million USD); 68.3% of them were attributable to the MOFs. Detailed information is provided in Supplementary Tables 25 to 35.

**Table 6:** Projected costs due to fragility fractures: Mexico 2023 (Mexican pesos). Data broken down by main categories of FF

Description	Hip	Vertebral	MOF	All FF
Direct medical costs	$7,292,116,837	$926,679,743	$10,305,041,190	$15,136,580,995
Acute care	$6,257,267,045	$264,494,346	$7,972,212,929	$11,723,408,804
Follow-up	$1,034,849,792	$662,185,397	$2,332,828,261	$3,413,172,191
Rehabilitation	$226,056,751	$139,102,727	$560,219,970	$877,505,581
Specialty visits	$168,455,539	$103,548,295	$341,300,748	$458,671,433
Laboratory test	$393,799,308	$288,093,172	$968,027,826	$1,454,681,749
Imaging tests	$116,836,852	$90,732,831	$268,778,198	$372,881,988
Drugs^a^	$129,701,341	$40,708,372	$194,501,518	$249,431,440
Indirect costs	$203,233,275	$88,036,073	$349,704,105	$456,630,604
Total costs	$7,495,350,112	$1,014,715,816	$10,654,745,295	$15,593,211,598

See Supplementary Tables 25 to 35 for details

*FF* fragility fractures, *MOF* major osteoporotic fractures

^a^Includes pharmacological treatment and supplementation with calcium plus vitamin D

**Table 7:** Projected costs due to fragility fractures: Mexico 2023 (Mexican pesos). Data broken down by health care sector

Description	Public sector	Private sector	Both sectors
Direct medical costs	$7,650,794,043	$7,485,786,951	$15,136,580,995
Acute care	$6,030,188,681	$5,693,220,123	$11,723,408,804
Follow-up	$1,620,605,362	$1,792,566,828	$3,413,172,191
Rehabilitation	$533,826,389	$343,679,193	$877,505,581
Specialty visits	$310,854,730	$147,816,703	$458,671,433
Laboratory test	$469,350,767	$985,330,982	$1,454,681,749
Imaging tests	$204,240,566	$168,641,423	$372,881,988
Drugs^a^	$102,332,912	$147,098,528	$249,431,440
Indirect costs	$340,189,800	$116,440,804	$456,630,604
Total costs	$7,990,983,843	$7,602,227,755	$15,593,211,598

See Supplementary Tables 25 to 36 for details

^a^Includes pharmacological treatment and supplementation with calcium plus vitamin D

Table 7 displays the projected costs for the public and the private healthcare sectors, expressed in Mexican pesos (see Supplementary Table 24 for estimates in USD). Given the higher cost vectors in the private sector, this sector had 48.8% of the total costs, whereas the public healthcare sector was responsible for the other 51.2% (7991 million Mexican pesos; 462.7 million USD; see Supplementary Table 36 for details).

## Discussion

In the current analysis, we estimated the total number of new FFs that would occur in the year 2023 in adults 50 years and older in Mexico. The most frequent type of fracture identified through the model was hip fracture, with 53,842 cases (38,411 in women). This figure is in line with the projections undertaken by Johansson et al. in 2010 [25], and is relatively similar to the number of hip fractures predicted by Aziziyeh et al. [7] for the year 2022 (46,793 hip fractures). Compared to this later reference, the number of total FFs calculated in this study is slightly lower (229,239 vs. 256,701), and the difference could be due to different approaches to estimating the incidence of the fractures. FFs were 3.1 times more likely to occur in women than in men. As comparison, this ratio was around two in Borgström et al. [2]; this difference could be explained by the high frequency of forearm FFs which are much more common in the female population.

As expected, hip fractures led to the highest economic impact among FFs, accounting for 48.1% of the total economic burden. Our estimate of the economic impact of FFs in Mexico is quite notably higher than the figure published by Aziziyeh et al. [7]. The reasons for that are mainly the differences in the methodological approaches applied and partly because the values are expressed in different years (2023 vs. 2018, respectively). Considering a gross domestic product (GDP) of 32,022.251 billion Mexican pesos [26] and that Mexico spends 5.5% of its GDP on health [27], the projected financial toll of FFs is equivalent to 0.9% of the (public plus private) health expenditure expected in our country for 2023.

One of the strengths of the study is that it included more categories of FF than the previous analysis conducted by Carlos et al. [10]. A second strength is that it includes the classification published by Borgström et al. [2] A third strength consisted of the effort to estimate not only direct medical costs, but also indirect costs associated with sick days in the patients that were actively working at the time of the fractures. However, the human capital method may underestimate the economic impact due to FF for those retired. In addition, productivity losses in the patients are only part of indirect costs, with time of caregivers being also important. A fourth strength is that most of the epidemiologic data was gathered from local and/or official sources in Mexico. For example, the proportional distribution of the type of surgery after a hip fracture was obtained from the Mexican hip fracture audit [28]. It is also noteworthy that our estimate of the weighted cost of a hip fracture requiring surgical management ($123,689 Mexican pesos) is almost the same as the figure published by Viveros-García et al. ($124,957 Mexican pesos) [29].

Among the limitations, we can state that it was necessary to rely on published literature to adjust the incidence of vertebral fractures. Another limitation is that, given the scarcity of data, it was also needed to include several assumptions regarding the ratio of private to public discharges and the ratio of emergency visits to hospital discharges for other institutions different from SSA. When performing the cost analysis for the public sector, it would have been ideal to address them first by specific institution and then summed up to give a grand total. This was not feasible due to the lack of costs per DRG code in institutions other than IMSS and because the unitary costs per service we used are mainly generic instead of specific by the institution [20]. Yet it should be noted that the economic information provided by IMSS (unitary costs, cost by DRG code) is widely accepted as representative of the public health care system in Mexico due to its large number of beneficiaries. When performing the cost analysis for the private sector, it was also needed to include some assumptions. Therefore, the data should be taken with caution. Another limitation is that the economic burden imposed on informal caregivers was excluded from the analysis. Furthermore, to simplify the costing analysis, neither mortality nor persistence with pharmacologic treatment was accounted for. However, some patients may die during the acute phase of care or during the first year after sustaining the fracture, while others may discontinue their pharmacologic therapy before completing a year. By assigning a full year of follow-up costs to those cases, we are overestimating this cost item. In contrast, death of a patient may represent additional expenses in terms of hospitalization and specialty care needed at the end-of-life process. Likewise, fractures (especially the hip) continue to be associated with significant costs beyond the first year of fracture [2]. These two potential sources of additional costs might lead to an underestimation of the true economic impact of FF.

## Conclusion

Given their high incidence plus the significant costs associated with their pathway care, fragility fractures represent a serious health problem for Mexico. Hip fractures impose the highest economic impact among them. Better preventive and therapeutic strategies may help to mitigate their significant financial toll. Improvement in the diagnostic approach and policies that ensure market access to innovative therapies are key to reducing the clinical and economic burden of fragility fractures.

## Supplementary Information

Below is the link to the electronic supplementary material.

Supplementary file1 (DOCX 394 KB)

## Data Availability

The datasets used and/or analyzed during the current study are available from the corresponding author upon reasonable request.
