# Supplementary material for: Estimated frequency and economic burden of incident fragility fractures during 2023 in Mexico
Source: Arch Osteoporos. 2024 Nov 7;19(1):109. doi: 10.1007/s11657-024-01468-2 (PMC11541356; doi:10.1007/s11657-024-01468-2)
Supplement: Supplementary file 1 — Supplementary file1 (DOCX 394 KB) [file 11657_2024_1468_MOESM1_ESM.docx]

**Journal:** *Osteoporosis International*

**Supplementary Material**

**Estimated frequency and economic burden of incident**

**fragility fractures during 2023 in Mexico**

**List of Supplementary Tables**

[**Table S1.** Hospital discharges due to fractures in most of public institutions, Mexico 2019: men 3](#_Toc176392967)

[**Table S2** Hospital discharges due to fractures in most of public institutions, Mexico 2019: women 6](#_Toc176392968)

[**Table S3** Ratio of discharges due to fractures occurred in SEMAR vs. SSA, 2021 9](#_Toc176392969)

[**Table S4** Ratio of emergency room visits vs. hospital discharges due to fractures in SSA, 2019 10](#_Toc176392970)

[**Table S5** Percentage of all fracture caused by a fall on the same level, SSA 2019 11](#_Toc176392971)

[**Table S6** Estimate of fragility fractures broken down by categories, Mexico 2019: men 12](#_Toc176392972)

[**Table S7** Estimate of fragility fractures broken down by categories, Mexico 2019: women 16](#_Toc176392973)

[**Table S8** Ratio of the incidence of vertebral to hip fractures based on Kanis et al. (2001) 20](#_Toc176392974)

[**Table S9** Midyear population by sex and age-group, Mexico 2019 21](#_Toc176392975)

[**Table S10** Cost per event of fragility fracture by category: US Dollars 22](#_Toc176392976)

[**Table S11** Estimate of the acute phase cost for those hospitalized per event by type of fracture 23](#_Toc176392977)

[**Table S12** Estimate of the cost per event due to rehabilitation by type of fracture and sector 27](#_Toc176392978)

[**Table S13** Estimate of the cost per event due to specialty visits by type of fracture and sector 28](#_Toc176392979)

[**Table S14** Estimate of the cost per event due to laboratory tests by type of fracture: public sector 29](#_Toc176392980)

[**Table S15** Estimate of the cost per event due to laboratory tests by type of fracture: private sector 33](#_Toc176392981)

[**Table S16** Estimate of the cost per event due to imaging tests by type of fracture: public sector 37](#_Toc176392982)

[**Table S17** Estimate of the cost per event due to imaging tests by type of fracture: private sector 38](#_Toc176392983)

[**Table S18** Estimate of the cost per event due to drugs by type of fracture: public sector 39](#_Toc176392984)

[**Table S19** Estimate of the cost per event due to drugs by type of fracture: private sector 41](#_Toc176392985)

[**Table S20** Estimate of the indirect costs per event by type of fracture 43](#_Toc176392986)

[**Table S21** Estimated number of fragility fractures in men 50+ years 48](#_Toc176392987)

[**Table S22** Estimated number of fragility fractures in women 50+ years 53](#_Toc176392988)

[**Table S23** Estimated number of fragility fractures in all 50+ years 58](#_Toc176392989)

[**Table S24** Projected costs due to fragility fractures: Mexico 2023 (US Dollars) 63](#_Toc176392990)

[**Table S25** Projected costs by type of fragility fracture: hip 64](#_Toc176392991)

[**Table S26** Projected costs by type of fragility fracture: vertebral 65](#_Toc176392992)

[**Table S27** Projected costs by type of fragility fracture: proximal humerus 66](#_Toc176392993)

[**Table S28** Projected costs by type of fragility fracture: distal forearm 67](#_Toc176392994)

[**Table S29** Projected costs by type of fragility fracture: lumbar spine, pelvis 68](#_Toc176392995)

[**Table S30** Projected costs by type of fragility fracture: thorax, lumbar zone, pelvis 69](#_Toc176392996)

[**Table S31** Projected costs by type of fragility fracture: shoulder, upper arm 70](#_Toc176392997)

[**Table S32** Projected costs by type of fragility fracture: proximal forearm 71](#_Toc176392998)

[**Table S33** Projected costs by type of fragility fracture: other femoral 72](#_Toc176392999)

[**Table S34** Projected costs by type of fragility fracture: tibia and fibula 73](#_Toc176393000)

[**Table S35** Projected costs by category of type of fragility fracture 74](#_Toc176393001)

[**Table S36** Projected costs by category of health care sector 75](#_Toc176393002)

**Table** **S1.** Hospital discharges due to fractures in most of public institutions, Mexico 2019: men

| **Type of fracture / Institution** | **Age groups (years)** | | | | | |
| --- | --- | --- | --- | --- | --- | --- |
|  | **50-59** | **60-69** | **70-79** | **80-89** | **90+** | **Total (50+)** |
| **Hip** | | | | | | |
| SSA | 351 | 472 | 738 | 824 | 273 | **2,658** |
| IMSS | 456 | 826 | 1,469 | 1,604 | 573 | **4,928** |
| ISSSTE | 77 | 203 | 326 | 431 | 133 | **1,170** |
| PEMEX | 7 | 21 | 42 | 68 | 20 | **158** |
| IMSS-Bienestar | 4 | 4 | 11 | 5 | 2 | **26** |
| State services | 0 | 1 | 3 | 5 | 1 | **10** |
| **Vertebral** | | | | | | |
| SSA | 85 | 51 | 26 | 7 | 3 | **172** |
| IMSS | 203 | 143 | 61 | 23 | 0 | **430** |
| ISSSTE | 24 | 33 | 18 | 3 | 1 | **79** |
| PEMEX | 10 | 9 | 4 | 3 | 0 | **26** |
| IMSS-Bienestar | 2 | 0 | 0 | 0 | 0 | **2** |
| State services | 0 | 0 | 0 | 0 | 0 | **0** |
| **Proximal humerus** | | | | | | |
| SSA | 271 | 131 | 109 | 51 | 11 | **573** |
| IMSS | 363 | 325 | 230 | 121 | 27 | **1,066** |
| ISSSTE | 83 | 91 | 52 | 27 | 11 | **264** |
| PEMEX | 7 | 6 | 8 | 5 | 1 | **27** |
| IMSS-Bienestar | 1 | 1 | 3 | 0 | 0 | **5** |
| State services | 2 | 1 | 1 | 0 | 0 | **4** |
| **Distal forearm** | | | | | | |
| SSA | 319 | 133 | 74 | 28 | 2 | **556** |
| IMSS | 562 | 313 | 144 | 51 | 7 | **1,077** |
| ISSSTE | 65 | 50 | 27 | 6 | 1 | **149** |
| PEMEX | 18 | 17 | 11 | 5 | 0 | **51** |
| IMSS-Bienestar | 3 | 3 | 0 | 0 | 0 | **6** |
| State services | 1 | 0 | 0 | 0 | 0 | **1** |

**Table S1** Hospital discharges due to fractures in most of public institutions, Mexico 2019: men (cont.)

| **Type of fracture / Institution** | **Age groups (years)** | | | | | |
| --- | --- | --- | --- | --- | --- | --- |
|  | **50-59** | **60-69** | **70-79** | **80-89** | **90+** | **Total (50+)** |
| **Lumbar spine, pelvis** | | | | | | |
| SSA | 38 | 27 | 13 | 12 | 2 | **92** |
| IMSS | 52 | 33 | 25 | 11 | 4 | **125** |
| ISSSTE | 13 | 11 | 10 | 5 | 3 | **42** |
| PEMEX | 0 | 0 | 0 | 0 | 0 | **0** |
| IMSS-Bienestar | 1 | 0 | 0 | 0 | 0 | **1** |
| State services | 0 | 0 | 0 | 0 | 0 | **0** |
| **Thorax, lumbar zone, pelvis** | | | | | | |
| SSA | 145 | 99 | 67 | 30 | 5 | **346** |
| IMSS | 185 | 160 | 92 | 34 | 9 | **480** |
| ISSSTE | 19 | 43 | 19 | 7 | 3 | **91** |
| PEMEX | 1 | 8 | 3 | 4 | 0 | **16** |
| IMSS-Bienestar | 3 | 4 | 3 | 0 | 0 | **10** |
| State services | 1 | 1 | 0 | 0 | 0 | **2** |
| **Shoulder, upper arm** | | | | | | |
| SSA | 250 | 117 | 51 | 26 | 8 | **452** |
| IMSS | 465 | 258 | 95 | 43 | 4 | **865** |
| ISSSTE | 84 | 54 | 19 | 7 | 3 | **167** |
| PEMEX | 6 | 4 | 4 | 0 | 1 | **15** |
| IMSS-Bienestar | 9 | 2 | 2 | 1 | 0 | **14** |
| State services | 2 | 0 | 0 | 0 | 0 | **2** |
| **Proximal forearm** | | | | | | |
| SSA | 618 | 272 | 123 | 60 | 7 | **1,080** |
| IMSS | 815 | 502 | 238 | 108 | 18 | **1,681** |
| ISSSTE | 135 | 128 | 51 | 22 | 3 | **339** |
| PEMEX | 16 | 3 | 3 | 2 | 0 | **24** |
| IMSS-Bienestar | 3 | 3 | 0 | 2 | 1 | **9** |
| State services | 3 | 0 | 1 | 1 | 0 | **5** |

**Table S1** Hospital discharges due to fractures in most of public institutions, Mexico 2019: men (cont.)

| **Type of fracture / Institution** | **Age groups (years)** | | | | | |
| --- | --- | --- | --- | --- | --- | --- |
|  | **50-59** | **60-69** | **70-79** | **80-89** | **90+** | **Total (50+)** |
| **Other femoral** | | | | | | |
| SSA | 383 | 297 | 313 | 273 | 105 | **1,371** |
| IMSS | 456 | 421 | 466 | 436 | 128 | **1,907** |
| ISSSTE | 53 | 79 | 112 | 127 | 42 | **413** |
| PEMEX | 2 | 5 | 7 | 7 | 3 | **24** |
| IMSS-Bienestar | 0 | 2 | 1 | 4 | 1 | **8** |
| State services | 4 | 2 | 0 | 4 | 0 | **10** |
| **Tibia and fibula** | | | | | | |
| SSA | NA | NA | NA | NA | NA | **NA** |
| IMSS | NA | NA | NA | NA | NA | **NA** |
| ISSSTE | NA | NA | NA | NA | NA | **NA** |
| PEMEX | NA | NA | NA | NA | NA | **NA** |
| IMSS-Bienestar | NA | NA | NA | NA | NA | **NA** |
| State services | NA | NA | NA | NA | NA | **NA** |

SSA: Secretariat of Health; IMSS: Mexican Social Security Institute; ISSSTE: Institute of Security and Social Services of State Workers; PEMEX: Mexican state-owned petroleum company; NA: not applicable because of this type of fracture was considered only in women.

Source: Dynamic Cubes of the General Direction of Health Information in the SSA [16].

**Table S2** Hospital discharges due to fractures in most of public institutions, Mexico 2019: women

| **Type of fracture / Institution** | **Age groups (years)** | | | | | |
| --- | --- | --- | --- | --- | --- | --- |
|  | **50-59** | **60-69** | **70-79** | **80-89** | **90+** | **Total (50+)** |
| **Hip** | | | | | | |
| SSA | 297 | 693 | 1,532 | 2,071 | 660 | **5,253** |
| IMSS | 476 | 1,418 | 3,064 | 3,691 | 1,124 | **9,773** |
| ISSSTE | 118 | 361 | 854 | 1,231 | 379 | **2,943** |
| PEMEX | 3 | 31 | 86 | 169 | 66 | **355** |
| IMSS-Bienestar | 1 | 5 | 13 | 36 | 9 | **64** |
| State services | 1 | 2 | 5 | 12 | 4 | **24** |
| **Vertebral** | | | | | | |
| SSA | 46 | 34 | 32 | 9 | 0 | **121** |
| IMSS | 120 | 130 | 88 | 27 | 4 | **369** |
| ISSSTE | 33 | 28 | 34 | 15 | 2 | **112** |
| PEMEX | 14 | 12 | 8 | 6 | 1 | **41** |
| IMSS-Bienestar | 0 | 0 | 0 | 0 | 0 | **0** |
| State services | 0 | 0 | 1 | 0 | 0 | **1** |
| **Proximal humerus** | | | | | | |
| SSA | 243 | 277 | 237 | 170 | 26 | **953** |
| IMSS | 503 | 778 | 730 | 382 | 61 | **2,454** |
| ISSSTE | 129 | 190 | 213 | 149 | 16 | **697** |
| PEMEX | 11 | 28 | 28 | 9 | 1 | **77** |
| IMSS-Bienestar | 2 | 5 | 2 | 6 | 1 | **16** |
| State services | 3 | 0 | 1 | 0 | 0 | **4** |
| **Distal forearm** | | | | | | |
| SSA | 436 | 400 | 269 | 147 | 23 | **1,275** |
| IMSS | 946 | 980 | 751 | 335 | 29 | **3,041** |
| ISSSTE | 172 | 218 | 135 | 74 | 22 | **621** |
| PEMEX | 51 | 58 | 62 | 31 | 3 | **205** |
| IMSS-Bienestar | 1 | 5 | 2 | 2 | 0 | **10** |
| State services | 1 | 2 | 1 | 0 | 0 | **4** |

**Table S2** Hospital discharges due to fractures in most of public institutions, Mexico 2019: women (cont.)

| **Type of fracture / Institution** | **Age groups (years)** | | | | | |
| --- | --- | --- | --- | --- | --- | --- |
|  | **50-59** | **60-69** | **70-79** | **80-89** | **90+** | **Total (50+)** |
| **Lumbar spine, pelvis** | | | | | | |
| SSA | 27 | 15 | 30 | 26 | 6 | **104** |
| IMSS | 29 | 26 | 30 | 27 | 5 | **117** |
| ISSSTE | 8 | 13 | 14 | 16 | 2 | **53** |
| PEMEX | 0 | 0 | 0 | 0 | 1 | **1** |
| IMSS-Bienestar | 1 | 0 | 0 | 0 | 1 | **2** |
| State services | 0 | 0 | 0 | 0 | 1 | **1** |
| **Thorax, lumbar zone, pelvis** | | | | | | |
| SSA | 40 | 42 | 22 | 21 | 12 | **137** |
| IMSS | 65 | 58 | 75 | 47 | 14 | **259** |
| ISSSTE | 18 | 15 | 16 | 21 | 4 | **74** |
| PEMEX | 2 | 7 | 5 | 3 | 1 | **18** |
| IMSS-Bienestar | 2 | 2 | 2 | 1 | 0 | **7** |
| State services | 0 | 1 | 0 | 0 | 0 | **1** |
| **Shoulder, upper arm** | | | | | | |
| SSA | 125 | 118 | 99 | 48 | 15 | **405** |
| IMSS | 183 | 293 | 226 | 118 | 26 | **846** |
| ISSSTE | 52 | 76 | 57 | 42 | 5 | **232** |
| PEMEX | 5 | 10 | 6 | 9 | 1 | **31** |
| IMSS-Bienestar | 1 | 1 | 0 | 3 | 0 | **5** |
| State services | 0 | 1 | 0 | 0 | 0 | **1** |
| **Proximal forearm** | | | | | | |
| SSA | 708 | 614 | 409 | 206 | 36 | **1,973** |
| IMSS | 1,109 | 1,216 | 831 | 340 | 57 | **3,553** |
| ISSSTE | 308 | 326 | 242 | 115 | 24 | **1,015** |
| PEMEX | 8 | 11 | 15 | 5 | 1 | **40** |
| IMSS-Bienestar | 6 | 3 | 2 | 3 | 0 | **14** |
| State services | 4 | 3 | 1 | 0 | 0 | **8** |

**Table S2** Hospital discharges due to fractures in most of public institutions, Mexico 2019: women (cont.)

| **Type of fracture / Institution** | **Age groups (years)** | | | | | |
| --- | --- | --- | --- | --- | --- | --- |
|  | **50-59** | **60-69** | **70-79** | **80-89** | **90+** | **Total (50+)** |
| **Other femoral** | | | | | | |
| SSA | 273 | 457 | 665 | 721 | 227 | **2,343** |
| IMSS | 362 | 736 | 1,136 | 1,050 | 277 | **3,561** |
| ISSSTE | 56 | 152 | 272 | 374 | 114 | **968** |
| PEMEX | 2 | 12 | 17 | 43 | 12 | **86** |
| IMSS-Bienestar | 1 | 4 | 5 | 3 | 2 | **15** |
| State services | 2 | 4 | 8 | 4 | 5 | **23** |
| **Tibia and fibula** | | | | | | |
| SSA | 629 | 431 | 205 | 107 | 27 | **1,399** |
| IMSS | 1,099 | 873 | 439 | 176 | 40 | **2,627** |
| ISSSTE | 234 | 199 | 91 | 46 | 12 | **582** |
| PEMEX | 36 | 26 | 18 | 5 | 1 | **86** |
| IMSS-Bienestar | 12 | 9 | 4 | 3 | 1 | **29** |
| State services | 2 | 1 | 1 | 1 | 0 | **5** |

SSA: Secretariat of Health; IMSS: Mexican Social Security Institute; ISSSTE: Institute of Security and Social Services of State Workers; PEMEX: Mexican state-owned petroleum company.

Source: Dynamic Cubes of the General Direction of Health Information in the SSA [16].

**Table S3** Ratio of discharges due to fractures occurred in SEMAR vs. SSA, 2021

| **Type of fracture** | **Age groups (years)** | | | | | |
| --- | --- | --- | --- | --- | --- | --- |
|  | **50-59** | **60-69** | **70-79** | **80-89** | **90+** | **Total (50+)** |
| Hip | 0.0020 | 0.0066 | 0.0114 | 0.0093 | 0.0120 | **0.0090** |
| Vertebral | 0.0348 | 0.0189 | 0.1034 | 0.0000 | 0.0000 | **0.0385** |
| Proximal humerus | 0.0125 | 0.0222 | 0.0246 | 0.0000 | 0.1071 | **0.0184** |
| Distal forearm | 0.0142 | 0.0362 | 0.0274 | 0.0000 | 0.0435 | **0.0225** |
| Lumbar spine, pelvis | 0.0000 | 0.0000 | 0.0000 | 0.0000 | 0.0000 | **0.0000** |
| Thorax, lumbar zone, pelvis | 0.0063 | 0.0360 | 0.0192 | 0.0000 | 0.0000 | **0.0163** |
| Shoulder, upper arm | 0.0124 | 0.0260 | 0.0085 | 0.0000 | 0.0000 | **0.0145** |
| Proximal forearm | 0.0076 | 0.0086 | 0.0125 | 0.0060 | 0.0000 | **0.0084** |
| Other femoral | 0.0032 | 0.0061 | 0.0056 | 0.0028 | 0.0050 | **0.0045** |
| Tibia and fibula | 0.0134 | 0.0054 | 0.0180 | 0.0000 | 0.0000 | **0.0105** |

SEMAR: Secretariat of the Navy; SSA: Secretariat of Health.

Source: Own estimates based on extracted data from the Dynamic Cubes of the General Direction of Health Information in the SSA [16].

**Table S4** Ratio of emergency room visits vs. hospital discharges due to fractures in SSA, 2019

| **Type of fracture** | **Age groups (years)** | | | | | |
| --- | --- | --- | --- | --- | --- | --- |
|  | **50-59** | **60-69** | **70-79** | **80-89** | **90+** | **Total (50+)** |
| **Hip** | | | | | | |
| Men | 0.4651 | 0.5678 | 0.4206 | 0.4764 | 0.5115 | **0.4793** |
| Women | 0.5772 | 0.5844 | 0.5146 | 0.4472 | 0.5558 | **0.5060** |
| **Vertebral** | | | | | | |
| Men | 0.8657 | 0.5002 | 0.6923 | 0.8571 | 0.6667 | **0.7273** |
| Women | 1.0689 | 1.0588 | 0.6967 | 1.8056 | 0.0000 | **1.0534** |
| **Proximal humerus** | | | | | | |
| Men | 0.9749 | 1.4920 | 1.5729 | 1.4254 | 2.0909 | **1.2684** |
| Women | 1.3932 | 1.8286 | 1.9881 | 1.8744 | 3.2308 | **1.8037** |
| **Distal forearm** | | | | | | |
| Men | 1.5274 | 2.2028 | 1.8713 | 3.0663 | 8.4091 | **1.8370** |
| Women | 3.1163 | 2.6105 | 2.8914 | 2.6899 | 3.0951 | **2.8606** |
| **Lumbar spine, pelvis** | | | | | | |
| Men | 0.8456 | 0.9544 | 1.2692 | 1.9375 | 2.0000 | **1.1049** |
| Women | 1.0672 | 2.8823 | 1.7095 | 2.6154 | 4.1250 | **2.0777** |
| **Thorax, lumbar zone, pelvis** | | | | | | |
| Men | 3.7073 | 2.8633 | 2.9006 | 3.0942 | 3.6000 | **3.2549** |
| Women | 6.3458 | 5.0423 | 8.8684 | 8.0744 | 3.6399 | **6.3792** |
| **Shoulder, upper arm** | | | | | | |
| Men | 2.3989 | 2.7601 | 3.3796 | 3.5488 | 3.7500 | **2.6931** |
| Women | 2.7066 | 3.0434 | 3.2886 | 4.2612 | 4.0667 | **3.1816** |
| **Proximal forearm** | | | | | | |
| Men | 1.5314 | 1.8482 | 2.3160 | 2.6175 | 6.1429 | **1.7907** |
| Women | 2.4179 | 2.2074 | 2.4135 | 2.5200 | 3.3333 | **2.3788** |
| **Other femoral** | | | | | | |
| Men | 0.5252 | 0.4680 | 0.7669 | 0.7435 | 0.5521 | **0.6135** |
| Women | 0.6202 | 0.5379 | 0.6067 | 0.6390 | 0.7388 | **0.6176** |
| **Tibia and fibula** | | | | | | |
| Men | NA | NA | NA | NA | NA | **NA** |
| Women | 1.5877 | 1.3437 | 1.3480 | 1.0246 | 1.2878 | **1.4286** |

SSA: Secretariat of Health; NA: not applicable.

Source: Own estimates based on extracted data from the Dynamic Cubes of the General Direction of Health Information in the SSA [16].

**Table S5** Percentage of all fracture caused by a fall on the same level, SSA 2019

| **Type of fracture** | **Age groups (years)** | | | | | |
| --- | --- | --- | --- | --- | --- | --- |
|  | **50-59** | **60-69** | **70-79** | **80-89** | **90+** | **Total (50+)** |
| **Hip** | | | | | | |
| Men | 0.5169 | 0.5961 | 0.7904 | 0.8111 | 0.9335 | **0.7500** |
| Women | 0.7450 | 0.8937 | 0.8929 | 0.8986 | 0.8959 | **0.8882** |
| **Vertebral** | | | | | | |
| Men^a^ | 0.246 | 0.433 | 0.590 | 0.839 | 0.839 | **0.491** |
| Women^a^ | 0.511 | 0.717 | 0.779 | 0.883 | 0.883 | **0.764** |
| **Proximal humerus** | | | | | | |
| Men | 0.4557 | 0.5005 | 0.6871 | 0.6044 | 0.9231 | **0.5495** |
| Women | 0.6332 | 0.7787 | 0.8320 | 0.7407 | 0.8333 | **0.7594** |
| **Distal forearm** | | | | | | |
| Men | 0.4416 | 0.4390 | 0.5313 | 0.6599 | 0.5000 | **0.4750** |
| Women | 0.7722 | 0.8362 | 0.8158 | 0.9192 | 0.9630 | **0.8225** |
| **Lumbar spine, pelvis** | | | | | | |
| Men^a^ | 0.286 | 0.339 | 0.535 | 0.810 | 0.810 | **0.519** |
| Women^a^ | 0.434 | 0.577 | 0.784 | 0.922 | 0.922 | **0.829** |
| **Thorax, lumbar zone, pelvis** | | | | | | |
| Men^a^ | 0.295 | 0.441 | 0.575 | 0.854 | 0.854 | **0.499** |
| Women^a^ | 0.402 | 0.579 | 0.743 | 0.893 | 0.893 | **0.759** |
| **Shoulder, upper arm** | | | | | | |
| Men | 0.3219 | 0.3874 | 0.5714 | 0.7391 | 0.7500 | **0.4002** |
| Women | 0.6043 | 0.6416 | 0.7607 | 0.7946 | 0.8692 | **0.6977** |
| **Proximal forearm** | | | | | | |
| Men | 0.4218 | 0.5227 | 0.5963 | 0.8781 | 0.6667 | **0.5128** |
| Women | 0.7536 | 0.7769 | 0.8493 | 0.8747 | 0.8618 | **0.7963** |
| **Other femoral** | | | | | | |
| Men | 0.4164 | 0.5091 | 0.7059 | 0.7214 | 0.7749 | **0.5999** |
| Women | 0.7084 | 0.8600 | 0.7525 | 0.8488 | 0.8676 | **0.8085** |
| **Tibia and fibula** | | | | | | |
| Men | NA | NA | NA | NA | NA | **NA** |
| Women | 0.7368 | 0.7015 | 0.6565 | 0.9655 | 0.5385 | **0.7214** |

SSA: Secretariat of Health; NA: not applicable.

^a^From Muschitz et al. [13]. Data for lumbar vertebra, pelvis, and rib, respectively.

Source: Own estimates based on the Dynamic Cubes of the General Direction of Health Information in the SSA [16].

**Table S6** Estimate of fragility fractures broken down by categories, Mexico 2019: men

| **Type of fracture / category of initial care** | **Age groups (years)** | | | | | |
| --- | --- | --- | --- | --- | --- | --- |
|  | **50-59** | **60-69** | **70-79** | **80-89** | **90+** | **Total (50+)** |
| **Hip** | **916** | **1,938** | **3,964** | **4,787** | **1,929** | **13,534** |
| Hospital discharge | 625 | 1,236 | 2,790 | 3,242 | 1,276 | **9,170** |
| Public sector | 464 | 919 | 2,078 | 2,412 | 950 | **6,824** |
| Private sector | 161 | 317 | 712 | 830 | 326 | **2,346** |
| Emergency visit only | 291 | 702 | 1,174 | 1,545 | 653 | **4,364** |
| Public sector | 216 | 522 | 874 | 1,149 | 486 | **3,247** |
| Private sector | 75 | 180 | 299 | 395 | 167 | **1,117** |
| **Vertebral^a^** | **3,033** | **2,599** | **3,444** | **2,130** | **429** | **11,634** |
| Hospital discharge | 111 | 140 | 94 | 41 | 5 | **391** |
| Public sector | 83 | 104 | 72 | 30 | 3 | **293** |
| Private sector | 28 | 36 | 22 | 11 | 1 | **98** |
| Emergency visit only | 96 | 70 | 65 | 35 | 3 | **269** |
| Public sector | 72 | 52 | 50 | 26 | 2 | **202** |
| Private sector | 24 | 18 | 16 | 9 | 1 | **67** |
| Specialty visit only^b^ | 2,826 | 2,389 | 3,284 | 2,054 | 422 | **10,974** |
| Public sector | 2,105 | 1,780 | 2,447 | 1,530 | 314 | **8,176** |
| Private sector | 721 | 609 | 837 | 524 | 107 | **2,798** |
| **Proximal humerus** | **897** | **951** | **982** | **404** | **209** | **3,442** |
| Hospital discharge | 454 | 382 | 382 | 166 | 68 | **1,451** |
| Public sector | 339 | 285 | 286 | 123 | 51 | **1,084** |
| Private sector | 115 | 97 | 96 | 43 | 16 | **367** |

**Table S6** Estimate of fragility fractures broken down by categories, Mexico 2019: men (cont.)

| **Type of fracture / category of initial care** | **Age groups (years)** | | | | | |
| --- | --- | --- | --- | --- | --- | --- |
|  | **50-59** | **60-69** | **70-79** | **80-89** | **90+** | **Total (50+)** |
| Emergency visit only | 443 | 569 | 600 | 237 | 141 | **1,991** |
| Public sector | 330 | 425 | 450 | 176 | 107 | **1,488** |
| Private sector | 112 | 144 | 151 | 61 | 34 | **503** |
| **Distal forearm** | **1,481** | **1,010** | **542** | **326** | **65** | **3,425** |
| Hospital discharge | 586 | 315 | 189 | 80 | 7 | **1,177** |
| Public sector | 437 | 237 | 141 | 59 | 5 | **880** |
| Private sector | 149 | 79 | 48 | 21 | 2 | **298** |
| Emergency visit only | 895 | 695 | 353 | 246 | 59 | **2,248** |
| Public sector | 668 | 521 | 264 | 182 | 44 | **1,679** |
| Private sector | 227 | 173 | 89 | 64 | 15 | **568** |
| **Lumbar spine, pelvis** | **74** | **63** | **79** | **90** | **30** | **335** |
| Hospital discharge | 40 | 32 | 35 | 31 | 10 | **148** |
| Public sector | 30 | 24 | 26 | 23 | 7 | **109** |
| Private sector | 10 | 8 | 9 | 8 | 3 | **38** |
| Emergency visit only | 34 | 31 | 44 | 59 | 20 | **188** |
| Public sector | 25 | 23 | 33 | 44 | 15 | **139** |
| Private sector | 9 | 8 | 11 | 15 | 5 | **49** |
| **Thorax, lumbar zone, pelvis** | **668** | **751** | **569** | **354** | **90** | **2,431** |
| Hospital discharge | 142 | 194 | 146 | 86 | 20 | **588** |
| Public sector | 106 | 146 | 109 | 64 | 15 | **440** |
| Private sector | 36 | 48 | 36 | 22 | 5 | **148** |

**Table S6** Estimate of fragility fractures broken down by categories, Mexico 2019: men (cont.)

| **Type of fracture / category of initial care** | **Age groups (years)** | | | | | |
| --- | --- | --- | --- | --- | --- | --- |
|  | **50-59** | **60-69** | **70-79** | **80-89** | **90+** | **Total (50+)** |
| Emergency visit only | 526 | 556 | 423 | 267 | 71 | **1,843** |
| Public sector | 392 | 419 | 317 | 198 | 52 | **1,379** |
| Private sector | 134 | 137 | 106 | 69 | 18 | **464** |
| **Shoulder, upper arm** | **1,217** | **876** | **581** | **348** | **77** | **3,099** |
| Hospital discharge | 358 | 233 | 133 | 77 | 16 | **816** |
| Public sector | 268 | 174 | 99 | 57 | 12 | **610** |
| Private sector | 91 | 59 | 34 | 20 | 4 | **207** |
| Emergency visit only | 859 | 643 | 448 | 272 | 61 | **2,283** |
| Public sector | 642 | 481 | 334 | 202 | 45 | **1,704** |
| Private sector | 217 | 162 | 114 | 70 | 16 | **579** |
| **Proximal forearm** | **2,313** | **1,840** | **1,124** | **838** | **185** | **6,300** |
| Hospital discharge | 914 | 646 | 339 | 232 | 26 | **2,156** |
| Public sector | 680 | 480 | 252 | 173 | 19 | **1,605** |
| Private sector | 234 | 165 | 87 | 59 | 7 | **551** |
| Emergency visit only | 1,399 | 1,194 | 785 | 606 | 159 | **4,143** |
| Public sector | 1,042 | 888 | 585 | 452 | 119 | **3,085** |
| Private sector | 358 | 306 | 200 | 154 | 40 | **1,058** |
| **Other femoral** | **773** | **818** | **1,524** | **1,446** | **456** | **5,016** |
| Hospital discharge | 507 | 558 | 862 | 829 | 294 | **3,049** |
| Public sector | 376 | 415 | 641 | 617 | 218 | **2,267** |
| Private sector | 130 | 143 | 222 | 213 | 75 | **783** |

**Table S6** Estimate of fragility fractures broken down by categories, Mexico 2019: men (cont.)

| **Type of fracture / category of initial care** | **Age groups (years)** | | | | | |
| --- | --- | --- | --- | --- | --- | --- |
|  | **50-59** | **60-69** | **70-79** | **80-89** | **90+** | **Total (50+)** |
| Emergency visit only | 266 | 261 | 661 | 617 | 162 | **1,967** |
| Public sector | 198 | 194 | 491 | 458 | 120 | **1,462** |
| Private sector | 68 | 67 | 170 | 158 | 42 | **505** |
| **Tibia and fibula** | **NA** | **NA** | **NA** | **NA** | **NA** | **NA** |
| Hospital discharge | NA | NA | NA | NA | NA | **NA** |
| Public sector | NA | NA | NA | NA | NA | **NA** |
| Private sector | NA | NA | NA | NA | NA | **NA** |
| Emergency visit only | NA | NA | NA | NA | NA | **NA** |
| Public sector | NA | NA | NA | NA | NA | **NA** |
| Private sector | NA | NA | NA | NA | NA | **NA** |

NA: not applicable because of this type of fracture was considered only in women.

^a^Calculated by aplying the corresponding adjustment factors (see S8 next) to the total number of hip fractures.

^b^Calculated as the difference between the total number of vertebral fractures and the sum of hospital discharges or emergency visit only cases of vertebral fracture.

Source: Own estimates based on data extracted from references [12], [13], [15], [16], [17], and [18].

**Table S7** Estimate of fragility fractures broken down by categories, Mexico 2019: women

| **Type of fracture / category of initial care** | **Age groups (years)** | | | | | |
| --- | --- | --- | --- | --- | --- | --- |
|  | **50-59** | **60-69** | **70-79** | **80-89** | **90+** | **Total (50+)** |
| **Hip** | **1,423** | **4,824** | **10,243** | **12,754** | **4,264** | **33,509** |
| Hospital discharge | 902 | 3,045 | 6,763 | 8,813 | 2,741 | **22,264** |
| Public sector | 670 | 2,263 | 5,034 | 6,563 | 2,043 | **16,572** |
| Private sector | 233 | 782 | 1,728 | 2,250 | 698 | **5,692** |
| Emergency visit only | 521 | 1,779 | 3,480 | 3,941 | 1,524 | **11,245** |
| Public sector | 386 | 1,322 | 2,591 | 2,935 | 1,135 | **8,370** |
| Private sector | 134 | 457 | 890 | 1,006 | 388 | **2,875** |
| **Vertebral^a^** | **4,155** | **6,940** | **8,522** | **4,276** | **715** | **24,607** |
| Hospital discharge | 151 | 200 | 184 | 68 | 8 | **610** |
| Public sector | 113 | 148 | 139 | 50 | 6 | **457** |
| Private sector | 38 | 51 | 44 | 18 | 2 | **153** |
| Emergency visit only | 161 | 211 | 128 | 123 | 0 | **623** |
| Public sector | 121 | 157 | 97 | 91 | 0 | **466** |
| Private sector | 41 | 54 | 31 | 32 | 0 | **157** |
| Specialty visit only^b^ | 3,842 | 6,529 | 8,211 | 4,085 | 707 | **23,374** |
| Public sector | 2,863 | 4,864 | 6,117 | 3,044 | 526 | **17,413** |
| Private sector | 980 | 1,665 | 2,094 | 1,042 | 180 | **5,960** |
| **Proximal humerus** | **1,842** | **3,861** | **4,131** | **2,053** | **546** | **12,432** |
| Hospital discharge | 770 | 1,365 | 1,382 | 714 | 129 | **4,360** |
| Public sector | 573 | 1,018 | 1,031 | 530 | 99 | **3,252** |
| Private sector | 196 | 347 | 351 | 184 | 30 | **1,108** |

**Table S7** Estimate of fragility fractures broken down by categories, Mexico 2019: women (cont.)

| **Type of fracture / category of initial care** | **Age groups (years)** | | | | | |
| --- | --- | --- | --- | --- | --- | --- |
|  | **50-59** | **60-69** | **70-79** | **80-89** | **90+** | **Total (50+)** |
| Emergency visit only | 1,072 | 2,496 | 2,748 | 1,339 | 417 | **8,072** |
| Public sector | 799 | 1,862 | 2,050 | 994 | 319 | **6,024** |
| Private sector | 273 | 634 | 699 | 345 | 98 | **2,048** |
| **Distal forearm** | **6,987** | **6,980** | **5,337** | **2,694** | **429** | **22,427** |
| Hospital discharge | 1,697 | 1,933 | 1,371 | 730 | 105 | **5,837** |
| Public sector | 1,264 | 1,449 | 1,024 | 541 | 79 | **4,358** |
| Private sector | 433 | 484 | 347 | 189 | 26 | **1,479** |
| Emergency visit only | 5,290 | 5,047 | 3,965 | 1,964 | 324 | **16,590** |
| Public sector | 3,939 | 3,783 | 2,961 | 1,456 | 244 | **12,384** |
| Private sector | 1,350 | 1,264 | 1,004 | 507 | 80 | **4,206** |
| **Lumbar spine, pelvis** | **78** | **163** | **212** | **310** | **99** | **863** |
| Hospital discharge | 38 | 42 | 78 | 86 | 19 | **263** |
| Public sector | 28 | 31 | 58 | 64 | 15 | **196** |
| Private sector | 10 | 11 | 20 | 22 | 5 | **68** |
| Emergency visit only | 40 | 121 | 134 | 225 | 79 | **600** |
| Public sector | 30 | 90 | 99 | 166 | 61 | **446** |
| Private sector | 10 | 31 | 35 | 58 | 19 | **153** |
| **Thorax, lumbar zone, pelvis** | **508** | **612** | **1,197** | **1,014** | **173** | **3,505** |
| Hospital discharge | 69 | 101 | 121 | 112 | 37 | **441** |
| Public sector | 52 | 77 | 91 | 83 | 28 | **330** |
| Private sector | 18 | 25 | 31 | 29 | 10 | **111** |

**Table S7** Estimate of fragility fractures broken down by categories, Mexico 2019: women (cont.)

| **Type of fracture / category of initial care** | **Age groups (years)** | | | | | |
| --- | --- | --- | --- | --- | --- | --- |
|  | **50-59** | **60-69** | **70-79** | **80-89** | **90+** | **Total (50+)** |
| Emergency visit only | 439 | 511 | 1,076 | 903 | 136 | **3,064** |
| Public sector | 327 | 386 | 804 | 671 | 101 | **2,289** |
| Private sector | 111 | 125 | 272 | 232 | 35 | **775** |
| **Shoulder, upper arm** | **1,122** | **1,784** | **1,722** | **1,237** | **279** | **6,144** |
| Hospital discharge | 303 | 441 | 401 | 235 | 55 | **1,436** |
| Public sector | 226 | 330 | 298 | 175 | 41 | **1,069** |
| Private sector | 77 | 111 | 103 | 60 | 14 | **366** |
| Emergency visit only | 820 | 1,343 | 1,320 | 1,002 | 224 | **4,708** |
| Public sector | 611 | 1,003 | 981 | 745 | 166 | **3,506** |
| Private sector | 209 | 339 | 339 | 257 | 58 | **1,202** |
| **Proximal forearm** | **7,507** | **7,366** | **5,938** | **2,795** | **595** | **24,201** |
| Hospital discharge | 2,197 | 2,297 | 1,739 | 794 | 137 | **7,164** |
| Public sector | 1,634 | 1,708 | 1,295 | 590 | 102 | **5,329** |
| Private sector | 562 | 589 | 445 | 204 | 36 | **1,835** |
| Emergency visit only | 5,311 | 5,070 | 4,198 | 2,001 | 458 | **17,037** |
| Public sector | 3,952 | 3,770 | 3,125 | 1,488 | 339 | **12,674** |
| Private sector | 1,359 | 1,299 | 1,073 | 513 | 119 | **4,363** |
| **Other femoral** | **1,082** | **2,451** | **3,448** | **4,131** | **1,302** | **12,413** |
| Hospital discharge | 668 | 1,594 | 2,146 | 2,521 | 749 | **7,676** |
| Public sector | 496 | 1,185 | 1,596 | 1,871 | 557 | **5,706** |
| Private sector | 172 | 408 | 550 | 649 | 191 | **1,970** |

**Table S7** Estimate of fragility fractures broken down by categories, Mexico 2019: women (cont.)

| **Type of fracture / category of initial care** | **Age groups (years)** | | | | | |
| --- | --- | --- | --- | --- | --- | --- |
|  | **50-59** | **60-69** | **70-79** | **80-89** | **90+** | **Total (50+)** |
| Emergency visit only | 414 | 857 | 1,302 | 1,611 | 553 | **4,737** |
| Public sector | 308 | 638 | 968 | 1,196 | 412 | **3,521** |
| Private sector | 106 | 220 | 334 | 415 | 141 | **1,216** |
| **Tibia and fibula** | **5,246** | **3,428** | **1,602** | **889** | **134** | **11,298** |
| Hospital discharge | 2,027 | 1,463 | 682 | 439 | 59 | **4,670** |
| Public sector | 1,512 | 1,087 | 509 | 326 | 44 | **3,479** |
| Private sector | 515 | 375 | 173 | 113 | 15 | **1,190** |
| Emergency visit only | 3,219 | 1,965 | 920 | 450 | 76 | **6,629** |
| Public sector | 2,401 | 1,461 | 687 | 334 | 56 | **4,940** |
| Private sector | 817 | 504 | 233 | 116 | 19 | **1,689** |

^a^Calculated by aplying the corresponding adjustment factors (see S8 next) to the total number of hip fractures.

^b^Calculated as the difference between the total number of vertebral fractures and the sum of hospital discharges or emergency visit only cases of vertebral fracture.

Source: Own estimates based on data extracted from references [12], [13], [15], [16], [17], and [18].

**Table S8** Ratio of the incidence of vertebral to hip fractures based on Kanis et al. (2001)

| **Men** | **50-54 years** | **55-59 years** | **60-64 years** | **65-69 years** | **70-74 years** | **75-79 years** | **80-84 years** | **85-89 years** |
| --- | --- | --- | --- | --- | --- | --- | --- | --- |
| Incidence^a^, vertebral fracture | 195 | 119 | 226 | 242 | 499 | 619 | 933 | 1,194 |
| Incidence^a^, hip fracture | 42 | 68 | 134 | 274 | 495 | 940 | 1923 | 3,241 |
| Ratio: vertebral to hip fractures | 4.64 | 1.75 | 1.69 | 0.88 | 1.01 | 0.66 | 0.49 | 0.37 |
| Mid-year population, Mexico^b^ | 3,490,083 | 2,981,119 | 2,443,857 | 1,848,919 | 1,285,565 | 850,417 | 516,828 | 272,150 |
| Distribution of the population within each decade (e.g., 50-59) | 53.9% | 46.1% | 56.9% | 43.1% | 60.2% | 39.8% | 65.5% | 34.5% |
| **Weighted ratio: incidence of vertebral to hip fractures** | **50-59 years**  3.31 | | **60-69 years**  1.34 | | **70-79 years**  0.87 | | **80-89 years**  0.44 | |
|  | | | | | | | | |
| **Women** | **50-54 years** | **55-59 years** | **60-64 years** | **65-69 years** | **70-74 years** | **75-79 years** | **80-84 years** | **85-89 years** |
| Incidence^a^, vertebral fracture | 161 | 158 | 303 | 439 | 778 | 1,111 | 1,163 | 1,641 |
| Incidence^a^, hip fracture | 41 | 91 | 181 | 387 | 817 | 1,689 | 3,364 | 5,183 |
| Ratio: vertebral to hip fractures | 3.93 | 1.74 | 1.67 | 1.13 | 0.95 | 0.66 | 0.35 | 0.32 |
| Mid-year population, Mexico^b^ | 3,886,219 | 3,312,568 | 2,749,726 | 2,128,986 | 1,531,996 | 1,057,995 | 679,284 | 380,591 |
| Distribution of the population within each decade (e.g., 50-59) | 54.0% | 46.0% | 56.4% | 43.6% | 59.2% | 40.8% | 64.1% | 35.9% |
| **Weighted ratio: incidence of vertebral to hip fractures** | **50-59 years**  2.92 | | **60-69 years**  1.44 | | **70-79 years**  0.83 | | **80-89 years**  0.34 | |

^a^Per 100,000 per year as reported by From Kanis et al. (data from Sweden) [12].

^b^Data for year 2023 in Mexico as projected by CONAPO [15].

Note: During the analysis, the subgroup of 90+ years was assumed to have a weighted ratio equal to a half of value for the 80-89 subgroup.

Source: own estimate based on extracted data from Reference [12] and Reference [15].

**Table S9** Midyear population by sex and age-group, Mexico 2019

| **Sex** | **50 to 59 years** | **60 to 69 years** | **70 to 79 years** | **80 to 89 years** | **90 to 99 years** | **All 50+ years** |
| --- | --- | --- | --- | --- | --- | --- |
| Men | 5,908,141 | 3,698,885 | 1,850,767 | 699,922 | 134,831 | **12,292,546** |
| Women | 6,489,491 | 4,179,351 | 2,231,678 | 937,362 | 202,705 | **14,040,587** |

Source: Projections elaborated by the National Population Council in Mexico [15].

**Table S10** Cost per event of fragility fracture by category: US Dollars

| Type of fragility fracture | Direct medical costs: inpatient acute phase plus outpatient care given during the follow-up period^a^ | | | | | | Indirect costs |
| --- | --- | --- | --- | --- | --- | --- | --- |
|  | Acute phase^b^ | Rehabili-tation | Specialty visits | Laboratory tests | Imaging tests | Drugs^c^ | Productivity losses |
| Hip | $6,828 | $196.87 | $161.32 | $186.73 | $87.90 | $76.13 | $218.55 |
|  | $19,325 | $378.17 | $239.11 | $1,115.19 | $235.91 | $324.56 |  |
| Vertebral | $3,622 | $156.72 | $130.18 | $168.82 | $90.73 | $31.60 | $122.95 |
|  | $10,249 | $303.98 | $186.80 | $1,084.61 | $231.84 | $130.63 |  |
| Proximal humerus | $3,582 | $196.87 | $71.42 | $140.90 | $53.10 | $31.60 | $66.16 |
|  | $10,137 | $360.98 | $93.51 | $881.89 | $99.42 | $130.63 |  |
| Distal forearm | $3,588 | $180.46 | $76.30 | $148.45 | $55.56 | $5.33 | $70.77 |
|  | $10,154 | $351.48 | $96.99 | $902.36 | $130.55 | $21.94 |  |
| Lumbar spine, pelvis | $4,331 | $213.27 | $145.95 | $169.87 | $92.90 | $31.60 | $67.07 |
|  | $12,257 | $351.48 | $157.50 | $978.51 | $186.76 | $130.63 |  |
| Thorax, lumbar zone, pelvis | $5,545 | $66.49 | $58.55 | $133.37 | $55.22 | $31.60 | $79.06 |
|  | $15,692 | $81.42 | $72.41 | $825.69 | $105.49 | $130.63 |  |
| Shoulder, upper arm | $3,483 | $196.87 | $71.42 | $140.90 | $53.10 | $31.60 | $78.39 |
|  | $9,858 | $360.98 | $93.51 | $881.89 | $99.42 | $130.63 |  |
| Proximal forearm | $3,588 | $180.46 | $76.30 | $148.45 | $55.56 | $5.33 | $74.75 |
|  | $10,154 | $351.48 | $96.99 | $902.36 | $130.55 | $21.94 |  |
| Other femoral | $5,555 | $180.46 | $76.30 | $148.45 | $55.56 | $31.60 | $66.08 |
|  | $15,720 | $351.48 | $96.99 | $902.36 | $130.55 | $130.63 |  |
| Tibia and fibula | $4,175 | $217.59 | $78.06 | $135.33 | $52.40 | $31.60 | $71.03 |
|  | $11,815 | $316.65 | $107.77 | $813.94 | $90.14 | $130.63 |  |

^a^Each type of fracture has two rows: the upper value is the cost at the public sector whereas the lower value corresponds to the cost at the private sector. Productivity losses are the same for both sectors.

^b^For individuals who were hospitalized. Patients treated only at the emergency room or with a vertebral fracture diagnosed at the outpatient level without any inpatient care entail different costs (see text).

^c^Includes both pharmacological treatment and supplementation with calcium plus vitamin D.

Source: Own estimates. See Tables S11 to S20 to more details.

**Table S11** Estimate of the acute phase cost for those hospitalized per event by type of fracture

| **Type of FF / Cost per event^a^** | **Type of medical care** | **% of patients^b^** | **Type of DRG** | **DRG code** | **DRG cost^c^** | | **DRG % cases^d^** | **DRG weighted cost^e^** | |
| --- | --- | --- | --- | --- | --- | --- | --- | --- | --- |
|  |  |  |  |  | **MXN** | **USD** |  | **MXN** | **USD** |
| Hip  $117,933 MXN  $6,828 USD | Conservative treatment | 8.25% | Medical | 535 | $189,312 | $10,961 | 3.5% | $53,919 | $3,122 |
|  |  |  |  | 536 | $49,065 | $2,841 | 96.5% |  |  |
|  | Reduction with/without internal fixation | 47.53% | Surgical | 480 | $161,227 | $9,335 | 2.0% | $116,425 | $6,741 |
|  |  |  |  | 481 | $150,629 | $8,722 | 2.4% |  |  |
|  |  |  |  | 482 | $114,638 | $6,638 | 95.6% |  |  |
|  | Partial/total hip replacement | 44.22% | Surgical | 462 | $161,027 | $9,324 | 0.2% | $131,496 | $7,614 |
|  |  |  |  | 469 | $240,765 | $13,941 | 0.7% |  |  |
|  |  |  |  | 470 | $130,661 | $7,565 | 99.1% |  |  |
| Vertebral  $62,546 MXN  $3,622 USD | Conservative treatment | 68.75% | Medical | 551 | $75,101 | $4,348 | 0.6% | $45,170 | $2,615 |
|  |  |  |  | 552 | $44,977 | $2,604 | 99.4% |  |  |
|  | Vertebroplasty or kyphoplasty | 12.91% | Surgical | 515 | $353,780 | $20,484 | 1.0% | $91,046 | $5,272 |
|  |  |  |  | 516 | $140,636 | $8,143 | 1.6% |  |  |
|  |  |  |  | 517 | $87,465 | $5,064 | 97.3% |  |  |
|  | Laminoplasty or laminectomy | 18.34% | Surgical | 028 | $239,276 | $13,854 | 0.6% | $107,619 | $6,231 |
|  |  |  |  | 029 | $185,430 | $10,737 | 0.4% |  |  |
|  |  |  |  | 030 | $162,514 | $9,410 | 13.8% |  |  |
|  |  |  |  | 518 | $166,495 | $9,640 | 0.7% |  |  |
|  |  |  |  | 519 | $124,109 | $7,186 | 0.9% |  |  |
|  |  |  |  | 520 | $96,625 | $5,595 | 83.7% |  |  |

**Table S11** Estimate of the acute phase cost for those hospitalized per event by type of fracture (cont.)

| **Type of FF / Cost per event^a^** | **Type of medical care** | **% of patients^b^** | **Type of DRG** | **DRG code** | **DRG cost^c^** | | **DRG % cases^d^** | **DRG weighted cost^e^** | |
| --- | --- | --- | --- | --- | --- | --- | --- | --- | --- |
|  |  |  |  |  | **MXN** | **USD** |  | **MXN** | **USD** |
| Proximal humerus  $61,863 MXN  $3,582 USD | Conservative treatment | 47.50% | Medical | 562 | $61,029 | $3,534 | 0.4% | $26,024 | $1,507 |
|  |  |  |  | 563 | $25,880 | $1,498 | 99.6% |  |  |
|  | Reduction with/without internal fixation | 52.50% | Surgical | 492 | $293,553 | $16,997 | 0.4% | $94,289 | $5,459 |
|  |  |  |  | 493 | $176,034 | $10,193 | 1.6% |  |  |
|  |  |  |  | 494 | $92,051 | $5,330 | 98.0% |  |  |
| Distal forearm  $61,970 MXN  $3,588 USD | Conservative treatment | 30.00% | Medical | 562 | $61,029 | $3,534 | 0.4% | $26,024 | $1,507 |
|  |  |  |  | 563 | $25,880 | $1,498 | 99.6% |  |  |
|  | Reduction with/without internal fixation | 70.00% | Surgical | 510 | $118,474 | $6,860 | 0.3% | $77,375 | $4,480 |
|  |  |  |  | 511 | $98,175 | $5,684 | 1.0% |  |  |
|  |  |  |  | 512 | $77,043 | $4,461 | 98.7% |  |  |
| Lumbar spine, pelvis  $74,803 MXN  $4,331 USD | Conservative treatment | 43.75% | Medical | 535 | $189,312 | $10,961 | 3.5% | $53,919 | $3,122 |
|  |  |  |  | 536 | $49,065 | $2,841 | 96.5% |  |  |
|  | Reduction with/without internal fixation | 56.25% | Surgical | 515 | $353,780 | $20,484 | 1.0% | $91,046 | $5,272 |
|  |  |  |  | 516 | $140,636 | $8,143 | 1.6% |  |  |
|  |  |  |  | 517 | $87,465 | $5,064 | 97.3% |  |  |
| Thorax, lumbar zone, pelvis  $95,765 MXN  $5,545 USD | Conservative treatment | 80.00% | Medical | 205 | $144,507 | $8,367 | 42.5% | $96,944 | $5,613 |
|  |  |  |  | 206 | $61,757 | $3,576 | 57.5% |  |  |
|  | Reduction with/without internal fixation | 20.00% | Surgical | 515 | $353,780 | $20,484 | 1.0% | $91,046 | $5,272 |
|  |  |  |  | 516 | $140,636 | $8,143 | 1.6% |  |  |
|  |  |  |  | 517 | $87,465 | $5,064 | 97.3% |  |  |

**Table S11** Estimate of the acute phase cost for those hospitalized per event by type of fracture (cont.)

| **Type of FF / Cost per event^a^** | **Type of medical care** | **% of patients^b^** | **Type of DRG** | **DRG code** | **DRG cost^c^** | | **DRG % cases^d^** | **DRG weighted cost^e^** | |
| --- | --- | --- | --- | --- | --- | --- | --- | --- | --- |
|  |  |  |  |  | **MXN** | **USD** |  | **MXN** | **USD** |
| Shoulder, upper arm  $60,161 MXN  $3,483 USD | Conservative treatment | 47.50% | Medical | 562 | $61,029 | $3,534 | 0.4% | $26,024 | $1,507 |
|  |  |  |  | 563 | $25,880 | $1,498 | 99.6% |  |  |
|  | Reduction with/without internal fixation | 52.50% | Surgical | 515 | $353,780 | $20,484 | 1.0% | $91,046 | $5,272 |
|  |  |  |  | 516 | $140,636 | $8,143 | 1.6% |  |  |
|  |  |  |  | 517 | $87,465 | $5,064 | 97.3% |  |  |
| Proximal forearm  $61,970 MXN  $3,588 USD | Conservative treatment | 30.00% | Medical | 562 | $61,029 | $3,534 | 0.4% | $26,024 | $1,507 |
|  |  |  |  | 563 | $25,880 | $1,498 | 99.6% |  |  |
|  | Reduction with/without internal fixation | 70.00% | Surgical | 510 | $118,474 | $6,860 | 0.3% | $77,375 | $4,480 |
|  |  |  |  | 511 | $98,175 | $5,684 | 1.0% |  |  |
|  |  |  |  | 512 | $77,043 | $4,461 | 98.7% |  |  |
| Other femoral  $95,937 MXN  $5,555 USD | Conservative treatment | 30.00% | Medical | 533 | $64,030 | $3,707 | 1.7% | $48,131 | $2,787 |
|  |  |  |  | 534 | $47,856 | $2,771 | 98.3% |  |  |
|  | Reduction with/without internal fixation | 70.00% | Surgical | 480 | $161,227 | $9,335 | 2.0% | $116,425 | $2,787 |
|  |  |  |  | 481 | $150,629 | $8,722 | 2.4% |  |  |
|  |  |  |  | 482 | $114,638 | $6,638 | 95.6% |  |  |
| Tibia and fibula  $72,103 MXN  $4,175 USD | Conservative treatment | 32.50% | Medical | 562 | $61,029 | $3,534 | 0.4% | $26,024 | $1,507 |
|  |  |  |  | 563 | $25,880 | $1,498 | 99.6% |  |  |
|  | Reduction with/without internal fixation | 67.50% | Surgical | 492 | $293,553 | $16,997 | 0.4% | $94,289 | $5,459 |
|  |  |  |  | 493 | $176,034 | $10,193 | 1.6% |  |  |
|  |  |  |  | 494 | $92,051 | $5,330 | 98.0% |  |  |

DRG: diagnosis related groups. Exchange rate: 17.2708 Mexican pesos (MXN) per 1 US Dollar (USD).

^a^Sum product of the values in the column named `% of patients´ and the values in the column named `DRG weighted cost´.

^b^Data from experts.

^c^Values in MXN as reported in Reference [19].

^d^Derived from the cumulative number of cases reported for each DRG during the period 2014-2017 at the Mexican Social Security Institute.

^e^Sum product of the values in the column named `DRG cost´ and the values in the column named `DRG % cases´.

**Table S12** Estimate of the cost per event due to rehabilitation by type of fracture and sector

| **Type of sector and fragility fracture (anatomic site)** | **Percentage of patients** | **Sessions if received** | **Unit cost per session** | **Weighted cost per event** | |
| --- | --- | --- | --- | --- | --- |
|  |  |  |  | **MXN** | **USD** |
| **Public sector** | | | | | |
| Hip | 95.0% | 3.00 | $1,193 MXN  69.08 USD | $3,400 | $196.87 |
| Vertebral | 82.5% | 2.75 |  | $2,707 | $156.72 |
| Proximal humerus | 95.0% | 3.00 |  | $3,400 | $196.87 |
| Distal forearm | 95.0% | 2.75 |  | $3,117 | $180.46 |
| Lumbar spine, pelvis | 95.0% | 3.25 |  | $3,683 | $213.27 |
| Thorax, lumbar zone, pelvis | 55.0% | 1.75 |  | $1,148 | $66.49 |
| Shoulder, upper arm | 95.0% | 3.00 |  | $3,400 | $196.87 |
| Proximal forearm | 95.0% | 2.75 |  | $3,117 | $180.46 |
| Other femoral | 95.0% | 2.75 |  | $3,117 | $180.46 |
| Tibia and fibula | 90.0% | 3.50 |  | $3,758 | $217.59 |
| **Private sector** | | | | | |
| Hip | 95.0% | 5.50 | $1,250 MXN  72.38 USD | $6,531 | $378.17 |
| Vertebral | 80.0% | 5.25 |  | $5,250 | $303.98 |
| Proximal humerus | 95.0% | 5.25 |  | $6,234 | $360.98 |
| Distal forearm | 92.5% | 5.25 |  | $6,070 | $351.48 |
| Lumbar spine, pelvis | 92.5% | 5.25 |  | $6,070 | $351.48 |
| Thorax, lumbar zone, pelvis | 50.0% | 2.25 |  | $1,406 | $81.42 |
| Shoulder, upper arm | 95.0% | 5.25 |  | $6,234 | $360.98 |
| Proximal forearm | 92.5% | 5.25 |  | $6,070 | $351.48 |
| Other femoral | 92.5% | 5.25 |  | $6,070 | $351.48 |
| Tibia and fibula | 87.5% | 5.00 |  | $5,469 | $316.65 |

Exchange rate: 17.2708 Mexican pesos (MXN) per 1 US Dollar (USD).

Source: Own estimates with data from experts, reference [19] for the cost of a rehabilitation session in the public sector and the price of a rehabilitation session in a private medical center located in Mexico City.

**Table S13** Estimate of the cost per event due to specialty visits by type of fracture and sector

| **Type of sector and fragility fracture (anatomic site)** | **Percentage of patients** | **Visits if received** | **Unit cost per specialty visit** | **Weighted cost per event** | |
| --- | --- | --- | --- | --- | --- |
|  |  |  |  | **MXN** | **USD** |
| **Public sector** | | | | | |
| Hip | 52.9% | 3.0 | $1,757 MXN  101.73 USD | $2,786 | $161.32 |
| Vertebral | 47.1% | 2.7 |  | $2,248 | $130.18 |
| Proximal humerus | 30.7% | 2.3 |  | $1,233 | $71.42 |
| Distal forearm | 35.0% | 2.1 |  | $1,318 | $76.30 |
| Lumbar spine, pelvis | 52.9% | 2.7 |  | $2,521 | $145.95 |
| Thorax, lumbar zone, pelvis | 33.6% | 1.7 |  | $1,011 | $58.55 |
| Shoulder, upper arm | 30.7% | 2.3 |  | $1,233 | $71.42 |
| Proximal forearm | 35.0% | 2.1 |  | $1,318 | $76.30 |
| Other femoral | 35.0% | 2.1 |  | $1,318 | $76.30 |
| Tibia and fibula | 33.6% | 2.3 |  | $1,348 | $78.06 |
| **Private sector** | | | | | |
| Hip | 50.7% | 4.3 | $1,900 MXN  110.01 USD | $4,130 | $239.11 |
| Vertebral | 45.7% | 3.7 |  | $3,226 | $186.80 |
| Proximal humerus | 35.0% | 2.4 |  | $1,615 | $93.51 |
| Distal forearm | 34.3% | 2.6 |  | $1,675 | $96.99 |
| Lumbar spine, pelvis | 43.6% | 3.3 |  | $2,720 | $157.50 |
| Thorax, lumbar zone, pelvis | 30.7% | 2.1 |  | $1,251 | $72.41 |
| Shoulder, upper arm | 35.0% | 2.4 |  | $1,615 | $93.51 |
| Proximal forearm | 34.3% | 2.6 |  | $1,675 | $96.99 |
| Other femoral | 34.3% | 2.6 |  | $1,675 | $96.99 |
| Tibia and fibula | 42.9% | 2.3 |  | $1,861 | $107.77 |

Exchange rate: 17.2708 Mexican pesos (MXN) per 1 US Dollar (USD).

Source: Own estimates with data from experts, reference [19] for the cost of a specialty visit in the public sector and the price of a specialty visit in a private medical center located in Mexico City.

**Table S14** Estimate of the cost per event due to laboratory tests by type of fracture: public sector

| **Type of fracture and laboratory test** | **Laboratory tests** | | **Unit cost per test** | | **Weighted cost per event** | |
| --- | --- | --- | --- | --- | --- | --- |
|  | **% Patients** | **Frequency** | **MXN** | **USD** | **MXN** | **USD** |
| **Hip** | | | | | **$3,225** | **$186.73** |
| Blood count | 92.9% | 3.0 | $131 | $7.59 |  |  |
| Blood chemistry | 92.9% | 3.3 | $137 | $7.93 |  |  |
| Liver function | 92.9% | 2.4 | $437 | $25.30 |  |  |
| General urine | 88.6% | 2.4 | $103 | $5.96 |  |  |
| Serum calcium | 85.7% | 2.1 | $81 | $4.69 |  |  |
| Phosphorus | 90.0% | 1.7 | $81 | $4.69 |  |  |
| Alkaline phosphatase | 90.0% | 2.0 | $91 | $5.27 |  |  |
| Creatinine | 92.9% | 2.9 | $81 | $4.69 |  |  |
| Protein electrophoresis | 44.3% | 1.1 | $242 | $14.01 |  |  |
| N-Telopeptide | 30.0% | 1.0 | $586 | $33.93 |  |  |
| Calcium in urine | 40.7% | 1.3 | $81 | $4.69 |  |  |
| 25-hydroxyvitamin D | 77.1% | 1.6 | $123 | $7.12 |  |  |
| Parathyroid hormone | 54.3% | 1.0 | $168 | $9.73 |  |  |
| **Vertebral** | | | | | **$2,916** | **$168.82** |
| Blood count | 98.6% | 2.4 | $131 | $7.59 |  |  |
| Blood chemistry | 91.4% | 2.7 | $137 | $7.93 |  |  |
| Liver function | 84.9% | 2.3 | $437 | $25.30 |  |  |
| General urine | 92.9% | 2.1 | $103 | $5.96 |  |  |
| Serum calcium | 85.7% | 2.1 | $81 | $4.69 |  |  |
| Phosphorus | 90.0% | 1.7 | $81 | $4.69 |  |  |
| Alkaline phosphatase | 90.0% | 2.0 | $91 | $5.27 |  |  |
| Creatinine | 85.7% | 2.6 | $81 | $4.69 |  |  |
| Protein electrophoresis | 43.6% | 1.1 | $242 | $14.01 |  |  |
| N-Telopeptide | 30.4% | 1.0 | $586 | $33.93 |  |  |
| Calcium in urine | 41.4% | 1.3 | $81 | $4.69 |  |  |
| 25-hydroxyvitamin D | 80.0% | 1.6 | $123 | $7.12 |  |  |
| Parathyroid hormone | 47.1% | 1.0 | $168 | $9.73 |  |  |

**Table S14** Estimate of the cost per event due to laboratory tests by type of fracture: public sector (cont.)

| **Type of fracture and laboratory test** | **Laboratory tests** | | **Unit cost per test** | | **Weighted cost per event** | |
| --- | --- | --- | --- | --- | --- | --- |
|  | **% Patients** | **Frequency** | **MXN** | **USD** | **MXN** | **USD** |
| **Proximal humerus / Shoulder, upper arm** | | | | | **$2,433** | **$140.90** |
| Blood count | 92.9% | 2.3 | $131 | $7.59 |  |  |
| Blood chemistry | 92.9% | 2.3 | $137 | $7.93 |  |  |
| Liver function | 87.1% | 2.0 | $437 | $25.30 |  |  |
| General urine | 85.7% | 1.6 | $103 | $5.96 |  |  |
| Serum calcium | 80.0% | 2.0 | $81 | $4.69 |  |  |
| Phosphorus | 84.3% | 1.6 | $81 | $4.69 |  |  |
| Alkaline phosphatase | 82.9% | 1.7 | $91 | $5.27 |  |  |
| Creatinine | 82.9% | 2.1 | $81 | $4.69 |  |  |
| Protein electrophoresis | 27.1% | 1.0 | $242 | $14.01 |  |  |
| N-Telopeptide | 28.6% | 1.0 | $586 | $33.93 |  |  |
| Calcium in urine | 32.9% | 1.5 | $81 | $4.69 |  |  |
| 25-hydroxyvitamin D | 54.3% | 1.6 | $123 | $7.12 |  |  |
| Parathyroid hormone | 44.3% | 1.0 | $168 | $9.73 |  |  |
| **Distal forearm / Proximal forearm / Other femoral** | | | | | **$2,564** | **$148.45** |
| Blood count | 92.9% | 2.6 | $131 | $7.59 |  |  |
| Blood chemistry | 92.9% | 2.4 | $137 | $7.93 |  |  |
| Liver function | 88.6% | 2.0 | $437 | $25.30 |  |  |
| General urine | 85.7% | 1.9 | $103 | $5.96 |  |  |
| Serum calcium | 80.0% | 2.0 | $81 | $4.69 |  |  |
| Phosphorus | 84.3% | 1.6 | $81 | $4.69 |  |  |
| Alkaline phosphatase | 82.9% | 1.9 | $91 | $5.27 |  |  |
| Creatinine | 82.9% | 2.4 | $81 | $4.69 |  |  |
| Protein electrophoresis | 30.0% | 1.0 | $242 | $14.01 |  |  |
| N-Telopeptide | 30.0% | 1.0 | $586 | $33.93 |  |  |
| Calcium in urine | 32.9% | 1.5 | $81 | $4.69 |  |  |
| 25-hydroxyvitamin D | 64.3% | 1.6 | $123 | $7.12 |  |  |
| Parathyroid hormone | 30.7% | 1.0 | $168 | $9.73 |  |  |

**Table S14** Estimate of the cost per event due to laboratory tests by type of fracture: public sector (cont.)

| **Type of fracture and laboratory test** | **Laboratory tests** | | **Unit cost per test** | | **Weighted cost per event** | |
| --- | --- | --- | --- | --- | --- | --- |
|  | **% Patients** | **Frequency** | **MXN** | **USD** | **MXN** | **USD** |
| **Lumbar spine, pelvis** | | | | | **$2,934** | **$169.87** |
| Blood count | 97.1% | 3.0 | $131 | $7.59 |  |  |
| Blood chemistry | 82.9% | 3.1 | $137 | $7.93 |  |  |
| Liver function | 97.1% | 2.1 | $437 | $25.30 |  |  |
| General urine | 88.6% | 2.3 | $103 | $5.96 |  |  |
| Serum calcium | 81.4% | 2.1 | $81 | $4.69 |  |  |
| Phosphorus | 87.1% | 1.7 | $81 | $4.69 |  |  |
| Alkaline phosphatase | 87.1% | 1.9 | $91 | $5.27 |  |  |
| Creatinine | 88.6% | 2.7 | $81 | $4.69 |  |  |
| Protein electrophoresis | 30.7% | 1.0 | $242 | $14.01 |  |  |
| N-Telopeptide | 28.6% | 1.0 | $586 | $33.93 |  |  |
| Calcium in urine | 34.3% | 1.5 | $81 | $4.69 |  |  |
| 25-hydroxyvitamin D | 57.1% | 1.6 | $123 | $7.12 |  |  |
| Parathyroid hormone | 45.7% | 1.0 | $168 | $9.73 |  |  |
| **Thorax, lumbar zone, pelvis** | | | | | **$2,303** | **$133.37** |
| Blood count | 90.0% | 2.1 | $131 | $7.59 |  |  |
| Blood chemistry | 81.4% | 2.3 | $137 | $7.93 |  |  |
| Liver function | 92.9% | 1.8 | $437 | $25.30 |  |  |
| General urine | 82.9% | 1.6 | $103 | $5.96 |  |  |
| Serum calcium | 75.7% | 1.9 | $81 | $4.69 |  |  |
| Phosphorus | 71.4% | 1.6 | $81 | $4.69 |  |  |
| Alkaline phosphatase | 78.6% | 1.7 | $91 | $5.27 |  |  |
| Creatinine | 90.0% | 1.9 | $81 | $4.69 |  |  |
| Protein electrophoresis | 31.4% | 1.0 | $242 | $14.01 |  |  |
| N-Telopeptide | 28.6% | 1.0 | $586 | $33.93 |  |  |
| Calcium in urine | 31.4% | 1.5 | $81 | $4.69 |  |  |
| 25-hydroxyvitamin D | 51.4% | 1.6 | $123 | $7.12 |  |  |
| Parathyroid hormone | 42.9% | 1.0 | $168 | $9.73 |  |  |

**Table S14** Estimate of the cost per event due to laboratory tests by type of fracture: public sector (cont.)

| **Type of fracture and laboratory test** | **Laboratory tests** | | **Unit cost per test** | | **Weighted cost per event** | |
| --- | --- | --- | --- | --- | --- | --- |
|  | **% Patients** | **Frequency** | **MXN** | **USD** | **MXN** | **USD** |
| **Tibia and fibula** | | | | | **$2,337** | **$135.33** |
| Blood count | 92.9% | 2.3 | $131 | $7.59 |  |  |
| Blood chemistry | 92.9% | 2.4 | $137 | $7.93 |  |  |
| Liver function | 88.6% | 1.9 | $437 | $25.30 |  |  |
| General urine | 74.3% | 1.4 | $103 | $5.96 |  |  |
| Serum calcium | 80.0% | 1.9 | $81 | $4.69 |  |  |
| Phosphorus | 82.9% | 1.6 | $81 | $4.69 |  |  |
| Alkaline phosphatase | 82.9% | 1.7 | $91 | $5.27 |  |  |
| Creatinine | 81.4% | 1.9 | $81 | $4.69 |  |  |
| Protein electrophoresis | 26.4% | 1.0 | $242 | $14.01 |  |  |
| N-Telopeptide | 28.6% | 1.0 | $586 | $33.93 |  |  |
| Calcium in urine | 32.9% | 1.5 | $81 | $4.69 |  |  |
| 25-hydroxyvitamin D | 51.4% | 1.6 | $123 | $7.12 |  |  |
| Parathyroid hormone | 42.9% | 1.0 | $168 | $9.73 |  |  |

Exchange rate: 17.2708 Mexican pesos (MXN) per 1 US Dollar (USD).

Source: Own estimates with data from experts and references [20] and [21].

**Table S15** Estimate of the cost per event due to laboratory tests by type of fracture: private sector

| **Type of fracture and laboratory test** | **Laboratory tests** | | **Unit cost per test** | | **Weighted cost per event** | |
| --- | --- | --- | --- | --- | --- | --- |
|  | **% Patients** | **Frequency** | **MXN** | **USD** | **MXN** | **USD** |
| **Hip** | | | | | **$19,260** | **$1,115.19** |
| Blood count | 92.9% | 3.1 | $575 | $33.30 |  |  |
| Blood chemistry | 92.9% | 3.1 | $647 | $37.49 |  |  |
| Liver function | 92.9% | 2.3 | $1,891 | $109.49 |  |  |
| General urine | 85.7% | 2.7 | $192 | $11.10 |  |  |
| Serum calcium | 92.9% | 2.1 | $332 | $19.20 |  |  |
| Phosphorus | 92.9% | 1.9 | $272 | $15.77 |  |  |
| Alkaline phosphatase | 92.9% | 2.0 | $575 | $33.30 |  |  |
| Creatinine | 92.9% | 3.1 | $575 | $33.30 |  |  |
| Protein electrophoresis | 42.9% | 1.0 | $1,213 | $70.24 |  |  |
| N-Telopeptide | 37.9% | 1.3 | $2,585 | $149.69 |  |  |
| Calcium in urine | 39.3% | 1.5 | $316 | $18.29 |  |  |
| 25-hydroxyvitamin D | 95.7% | 2.1 | $2,276 | $131.81 |  |  |
| Parathyroid hormone | 42.1% | 1.4 | $1,259 | $72.88 |  |  |
| **Vertebral** | | | | | **$18,732** | **$1,084.61** |
| Blood count | 98.6% | 2.9 | $575 | $33.30 |  |  |
| Blood chemistry | 98.6% | 2.9 | $647 | $37.49 |  |  |
| Liver function | 98.6% | 2.1 | $1,891 | $109.49 |  |  |
| General urine | 85.7% | 2.4 | $192 | $11.10 |  |  |
| Serum calcium | 95.7% | 2.1 | $332 | $19.20 |  |  |
| Phosphorus | 92.9% | 1.9 | $272 | $15.77 |  |  |
| Alkaline phosphatase | 92.9% | 2.0 | $575 | $33.30 |  |  |
| Creatinine | 92.9% | 2.7 | $575 | $33.30 |  |  |
| Protein electrophoresis | 37.9% | 1.0 | $1,213 | $70.24 |  |  |
| N-Telopeptide | 35.0% | 1.3 | $2,585 | $149.69 |  |  |
| Calcium in urine | 42.1% | 1.5 | $316 | $18.29 |  |  |
| 25-hydroxyvitamin D | 95.7% | 2.1 | $2,276 | $131.81 |  |  |
| Parathyroid hormone | 40.7% | 1.5 | $1,259 | $72.88 |  |  |

**Table S15** Estimate of the cost per event due to laboratory tests by type of fracture: private sector (cont.)

| **Type of fracture and laboratory test** | **Laboratory tests** | | **Unit cost per test** | | **Weighted cost per event** | |
| --- | --- | --- | --- | --- | --- | --- |
|  | **% Patients** | **Frequency** | **MXN** | **USD** | **MXN** | **USD** |
| **Proximal humerus / Shoulder, upper arm** | | | | | **$15,231** | **$881.89** |
| Blood count | 92.9% | 2.3 | $575 | $33.30 |  |  |
| Blood chemistry | 92.9% | 2.4 | $647 | $37.49 |  |  |
| Liver function | 92.9% | 1.9 | $1,891 | $109.49 |  |  |
| General urine | 85.7% | 1.9 | $192 | $11.10 |  |  |
| Serum calcium | 92.9% | 2.0 | $332 | $19.20 |  |  |
| Phosphorus | 91.4% | 1.7 | $272 | $15.77 |  |  |
| Alkaline phosphatase | 90.0% | 1.9 | $575 | $33.30 |  |  |
| Creatinine | 90.0% | 2.6 | $575 | $33.30 |  |  |
| Protein electrophoresis | 33.7% | 1.0 | $1,213 | $70.24 |  |  |
| N-Telopeptide | 27.1% | 1.3 | $2,585 | $149.69 |  |  |
| Calcium in urine | 32.1% | 1.6 | $316 | $18.29 |  |  |
| 25-hydroxyvitamin D | 92.9% | 1.7 | $2,276 | $131.81 |  |  |
| Parathyroid hormone | 34.3% | 1.2 | $1,259 | $72.88 |  |  |
| **Distal forearm / Proximal forearm / Other femoral** | | | | | **$15,585** | **$902.36** |
| Blood count | 92.9% | 2.6 | $575 | $33.30 |  |  |
| Blood chemistry | 92.9% | 2.7 | $647 | $37.49 |  |  |
| Liver function | 92.9% | 1.9 | $1,891 | $109.49 |  |  |
| General urine | 85.7% | 2.1 | $192 | $11.10 |  |  |
| Serum calcium | 92.9% | 2.0 | $332 | $19.20 |  |  |
| Phosphorus | 91.4% | 1.7 | $272 | $15.77 |  |  |
| Alkaline phosphatase | 90.0% | 1.9 | $575 | $33.30 |  |  |
| Creatinine | 90.0% | 2.6 | $575 | $33.30 |  |  |
| Protein electrophoresis | 35.7% | 1.0 | $1,213 | $70.24 |  |  |
| N-Telopeptide | 25.7% | 1.3 | $2,585 | $149.69 |  |  |
| Calcium in urine | 33.6% | 1.6 | $316 | $18.29 |  |  |
| 25-hydroxyvitamin D | 92.9% | 1.7 | $2,276 | $131.81 |  |  |
| Parathyroid hormone | 34.3% | 1.2 | $1,259 | $72.88 |  |  |

**Table S15** Estimate of the cost per event due to laboratory tests by type of fracture: private sector (cont.)

| **Type of fracture and laboratory test** | **Laboratory tests** | | **Unit cost per test** | | **Weighted cost per event** | |
| --- | --- | --- | --- | --- | --- | --- |
|  | **% Patients** | **Frequency** | **MXN** | **USD** | **MXN** | **USD** |
| **Lumbar spine, pelvis** | | | | | **$16,900** | **$978.51** |
| Blood count | 97.1% | 2.6 | $575 | $33.30 |  |  |
| Blood chemistry | 97.1% | 2.9 | $647 | $37.49 |  |  |
| Liver function | 97.1% | 2.0 | $1,891 | $109.49 |  |  |
| General urine | 88.6% | 2.3 | $192 | $11.10 |  |  |
| Serum calcium | 94.3% | 2.1 | $332 | $19.20 |  |  |
| Phosphorus | 94.3% | 1.9 | $272 | $15.77 |  |  |
| Alkaline phosphatase | 94.3% | 2.0 | $575 | $33.30 |  |  |
| Creatinine | 92.9% | 2.7 | $575 | $33.30 |  |  |
| Protein electrophoresis | 37.1% | 1.0 | $1,213 | $70.24 |  |  |
| N-Telopeptide | 25.7% | 1.5 | $2,585 | $149.69 |  |  |
| Calcium in urine | 32.1% | 1.8 | $316 | $18.29 |  |  |
| 25-hydroxyvitamin D | 95.7% | 1.7 | $2,276 | $131.81 |  |  |
| Parathyroid hormone | 35.7% | 1.3 | $1,259 | $72.88 |  |  |
| **Thorax, lumbar zone, pelvis** | | | | | **$14,260** | **$825.69** |
| Blood count | 92.9% | 2.0 | $575 | $33.30 |  |  |
| Blood chemistry | 92.9% | 2.3 | $647 | $37.49 |  |  |
| Liver function | 92.9% | 1.7 | $1,891 | $109.49 |  |  |
| General urine | 85.7% | 1.7 | $192 | $11.10 |  |  |
| Serum calcium | 92.9% | 1.9 | $332 | $19.20 |  |  |
| Phosphorus | 90.0% | 1.7 | $272 | $15.77 |  |  |
| Alkaline phosphatase | 90.0% | 1.9 | $575 | $33.30 |  |  |
| Creatinine | 90.0% | 1.9 | $575 | $33.30 |  |  |
| Protein electrophoresis | 37.9% | 1.0 | $1,213 | $70.24 |  |  |
| N-Telopeptide | 24.3% | 1.5 | $2,585 | $149.69 |  |  |
| Calcium in urine | 30.7% | 1.8 | $316 | $18.29 |  |  |
| 25-hydroxyvitamin D | 90.0% | 1.7 | $2,276 | $131.81 |  |  |
| Parathyroid hormone | 33.6% | 1.3 | $1,259 | $72.88 |  |  |

**Table S15** Estimate of the cost per event due to laboratory tests by type of fracture: private sector (cont.)

| **Type of fracture and laboratory test** | **Laboratory tests** | | **Unit cost per test** | | **Weighted cost per event** | |
| --- | --- | --- | --- | --- | --- | --- |
|  | **% Patients** | **Frequency** | **MXN** | **USD** | **MXN** | **USD** |
| **Tibia and fibula** | | | | | **$14,057** | **$813.94** |
| Blood count | 92.9% | 2.1 | $575 | $33.30 |  |  |
| Blood chemistry | 92.9% | 2.4 | $647 | $37.49 |  |  |
| Liver function | 92.9% | 1.7 | $1,891 | $109.49 |  |  |
| General urine | 85.7% | 1.6 | $192 | $11.10 |  |  |
| Serum calcium | 92.9% | 1.9 | $332 | $19.20 |  |  |
| Phosphorus | 90.0% | 1.7 | $272 | $15.77 |  |  |
| Alkaline phosphatase | 90.0% | 1.9 | $575 | $33.30 |  |  |
| Creatinine | 90.0% | 2.0 | $575 | $33.30 |  |  |
| Protein electrophoresis | 33.6% | 1.0 | $1,213 | $70.24 |  |  |
| N-Telopeptide | 24.3% | 1.5 | $2,585 | $149.69 |  |  |
| Calcium in urine | 30.7% | 1.8 | $316 | $18.29 |  |  |
| 25-hydroxyvitamin D | 75.7% | 1.8 | $2,276 | $131.81 |  |  |
| Parathyroid hormone | 32.9% | 1.3 | $1,259 | $72.88 |  |  |

Exchange rate: 17.2708 Mexican pesos (MXN) per 1 US Dollar (USD).

Source: Own estimates with data from experts and prices from a private medical center located in Mexico City.

**Table S16** Estimate of the cost per event due to imaging tests by type of fracture: public sector

| **Type of fracture and imaging test** | **Imaging tests** | | **Unit cost per test** | | **Weighted cost per event** | |
| --- | --- | --- | --- | --- | --- | --- |
|  | **% Patients** | **Frequency** | **MXN** | **USD** | **MXN** | **USD** |
| **Hip** | | | | | **$1,518** | **$87.90** |
| Radiography | 80.0% | 1.3 | $382 | $22.12 |  |  |
| Computed tomography | 30.7% | 1.0 | $1,626 | $94.15 |  |  |
| DEXA | 92.9% | 1.0 | $674 | $39.03 |  |  |
| **Vertebral** | | | | | **$1,567** | **$90.73** |
| Radiography | 82.9% | 1.3 | $382 | $22.12 |  |  |
| Computed tomography | 32.9% | 1.0 | $1,626 | $94.15 |  |  |
| DEXA | 92.9% | 1.0 | $674 | $39.03 |  |  |
| **Proximal humerus / Shoulder, upper arm** | | | | | **$917** | **$53.10** |
| Radiography | 79.9% | 1.1 | $382 | $22.12 |  |  |
| Computed tomography | 3.6% | 1.0 | $1,626 | $94.15 |  |  |
| DEXA | 75.7% | 1.0 | $674 | $39.03 |  |  |
| **Distal forearm / Proximal forearm / Other femoral** | | | | | **$960** | **$55.56** |
| Radiography | 72.9% | 1.1 | $382 | $22.12 |  |  |
| Computed tomography | 2.1% | 1.0 | $1,626 | $94.15 |  |  |
| DEXA | 90.0% | 1.0 | $674 | $39.03 |  |  |
| **Lumbar spine, pelvis** | | | | | **$1,604** | **$92.90** |
| Radiography | 81.4% | 1.4 | $382 | $22.12 |  |  |
| Computed tomography | 32.9% | 1.0 | $1,626 | $94.15 |  |  |
| DEXA | 92.9% | 1.0 | $674 | $39.03 |  |  |
| **Thorax, lumbar zone, pelvis** | | | | | **$954** | **$55.22** |
| Radiography | 72.9% | 1.3 | $382 | $22.12 |  |  |
| Computed tomography | 5.9% | 1.0 | $1,626 | $94.15 |  |  |
| DEXA | 74.3% | 1.0 | $674 | $39.03 |  |  |
| **Tibia and fibula** | | | | | **$905** | **$52.40** |
| Radiography | 72.9% | 1.3 | $382 | $22.12 |  |  |
| Computed tomography | 2.9% | 1.0 | $1,626 | $94.15 |  |  |
| DEXA | 74.3% | 1.0 | $674 | $39.03 |  |  |

DEXA: Dual-energy X-ray absorptiometry. Exchange rate: 17.2708 Mexican pesos (MXN) per 1 US Dollar (USD).

Source: Own estimates with data from experts and reference [20].

**Table S17** Estimate of the cost per event due to imaging tests by type of fracture: private sector

| **Type of fracture and imaging test** | **Imaging tests** | | **Unit cost per test** | | **Weighted cost per event** | |
| --- | --- | --- | --- | --- | --- | --- |
|  | **% Patients** | **Frequency** | **MXN** | **USD** | **MXN** | **USD** |
| **Hip** | | | | | **$4,074** | **$235.91** |
| Radiography | 92.9% | 1.7 | $593 | $34.32 |  |  |
| Computed tomography | 48.6% | 1.0 | $4,916 | $284.64 |  |  |
| DEXA | 92.9% | 1.0 | $800 | $46.32 |  |  |
| **Vertebral** | | | | | **$4,004** | **$231.84** |
| Radiography | 92.9% | 1.7 | $593 | $34.32 |  |  |
| Computed tomography | 47.1% | 1.0 | $4,916 | $284.64 |  |  |
| DEXA | 92.9% | 1.0 | $800 | $46.32 |  |  |
| **Proximal humerus / Shoulder, upper arm** | | | | | **$1,717** | **$99.42** |
| Radiography | 92.9% | 1.4 | $593 | $34.32 |  |  |
| Computed tomography | 4.3% | 1.0 | $4,916 | $284.64 |  |  |
| DEXA | 90.0% | 1.0 | $800 | $46.32 |  |  |
| **Distal forearm / Proximal forearm / Other femoral** | | | | | **$2,255** | **$130.55** |
| Radiography | 90.0% | 1.4 | $593 | $34.32 |  |  |
| Computed tomography | 15.7% | 1.0 | $4,916 | $284.64 |  |  |
| DEXA | 90.0% | 1.0 | $800 | $46.32 |  |  |
| **Lumbar spine, pelvis** | | | | | **$3,225** | **$186.76** |
| Radiography | 95.7% | 1.7 | $593 | $34.32 |  |  |
| Computed tomography | 30.7% | 1.0 | $4,916 | $284.64 |  |  |
| DEXA | 92.9% | 1.0 | $800 | $46.32 |  |  |
| **Thorax, lumbar zone, pelvis** | | | | | **$1,822** | **$105.49** |
| Radiography | 90.0% | 1.4 | $593 | $34.32 |  |  |
| Computed tomography | 7.1% | 1.0 | $4,916 | $284.64 |  |  |
| DEXA | 88.6% | 1.0 | $800 | $46.32 |  |  |
| **Tibia and fibula** | | | | | **$1,557** | **$90.14** |
| Radiography | 92.9% | 1.3 | $593 | $34.32 |  |  |
| Computed tomography | 2.9% | 1.0 | $4,916 | $284.64 |  |  |
| DEXA | 88.6% | 1.0 | $800 | $46.32 |  |  |

DEXA: Dual-energy X-ray absorptiometry. Exchange rate: 17.2708 Mexican pesos (MXN) per 1 US Dollar (USD).

Source: Own estimates with data from experts and prices from a private medical center located in Mexico City.

**Table S18** Estimate of the cost per event due to drugs by type of fracture: public sector

| **Type of fracture and drug or supplementation** | **Drugs** | | **Unit cost per pack** | | **Weighted cost per event** | |
| --- | --- | --- | --- | --- | --- | --- |
|  | **% Patients** | **Packs per year** | **MXN** | **USD** | **MXN** | **USD** |
| **Hip** | | | | | **$1,315** | **$76.13** |
| Alendronic acid 10 mg tablets (pack of 30) | 6.6% | 12.175 | $12.37 | $0.72 |  |  |
| Alendronic acid 70 mg tablets (pack of 4) | 1.1% | 13.045 | $11.25 | $0.65 |  |  |
| Risedronic acid 5 mg pills (pack of 28) | 0.5% | 13.045 | $13.30 | $0.77 |  |  |
| Risedronic acid 35 mg pills (pack of 4) | 8.3% | 13.045 | $11.00 | $0.64 |  |  |
| Zoledronic acid 4 mg vial (pack of 1) | 0.1% | 13.045 | $370.19^a^ | $21.43^a^ |  |  |
| Denosumab 60 mg prefilled syrange (pack of 1) | 4.8% | 2.000 | $3,521.58 | $203.90 |  |  |
| Teriparatide 250 µg prefilled pen (pack of 1)^b^ | 1.2% | 13.045 | $4,180.00 | $242.03 |  |  |
| Calcium carbonate / vitamin D3 tablets (pack of 30) | 31.8% | 24.350 | $38.90 | $2.25 |  |  |
| **Vertebral^c^** | | | | | **$546** | **$31.60** |
| Alendronic acid 10 mg tablets (pack of 30) | 3.1% | 12.175 | $12.37 | $0.72 |  |  |
| Alendronic acid 70 mg tablets (pack of 4) | 0.5% | 13.045 | $11.25 | $0.65 |  |  |
| Risedronic acid 5 mg pills (pack of 28) | 0.2% | 13.045 | $13.30 | $0.77 |  |  |
| Risedronic acid 35 mg pills (pack of 4) | 3.8% | 13.045 | $11.00 | $0.64 |  |  |
| Zoledronic acid 4 mg vial (pack of 1) | 0.1% | 13.045 | $370.19^a^ | $21.43^a^ |  |  |
| Denosumab 60 mg prefilled syrange (pack of 1) | 2.2% | 2.000 | $3,521.58 | $203.90 |  |  |
| Teriparatide 250 µg prefilled pen^b^ (pack of 1) | 0.6% | 13.045 | $4,180.00 | $242.03 |  |  |
| Calcium carbonate / vitamin D3 tablets (pack of 30) | 7.9% | 24.350 | $38.90 | $2.25 |  |  |

**Table S18** Estimate of the cost per event due to drugs by type of fracture: public sector (cont.)

| **Type of fracture and drug or supplementation** | **Drugs** | | **Unit cost per pack** | | **Weighted cost per event** | |
| --- | --- | --- | --- | --- | --- | --- |
|  | **% Patients** | **Packs per year** | **MXN** | **USD** | **MXN** | **USD** |
| **Distal forearm / Proximal forearm** | | | | | **$92** | **$5.33** |
| Alendronic acid 10 mg tablets (pack of 30) | 0.5% | 12.175 | $12.37 | $0.72 |  |  |
| Alendronic acid 70 mg tablets (pack of 4) | 0.1% | 13.045 | $11.25 | $0.65 |  |  |
| Risedronic acid 5 mg pills (pack of 28) | 0.0% | 13.045 | $13.30 | $0.77 |  |  |
| Risedronic acid 35 mg pills (pack of 4) | 0.7% | 13.045 | $11.00 | $0.64 |  |  |
| Zoledronic acid 4 mg vial (pack of 1) | 0.0% | 13.045 | $370.19^a^ | $21.43^a^ |  |  |
| Denosumab 60 mg prefilled syrange (pack of 1) | 0.4% | 2.000 | $3,521.58 | $203.90 |  |  |
| Teriparatide 250 µg prefilled pen (pack of 1)^b^ | 0.1% | 13.045 | $4,180.00 | $242.03 |  |  |
| Calcium carbonate / vitamin D3 tablets (pack of 30) | 1.2% | 24.350 | $38.90 | $2.25 |  |  |

^a^Includes an administration cost of $322 MXN (18.64 USD) equivalent to a procedure of an intravenous infusion.

^b^Containing 28 daily doses of 20 mcg.

^c^Applies also to the rest of type of fractures included in the analysis.

Exchange rate: 17.2708 Mexican pesos (MXN) per 1 US Dollar (USD).

Source: Own estimates with data from references [19], [22] and [23].

**Table S19** Estimate of the cost per event due to drugs by type of fracture: private sector

| **Type of fracture and drug or supplementation** | **Drugs** | | **Unit cost per pack** | | **Weighted cost per event** | |
| --- | --- | --- | --- | --- | --- | --- |
|  | **% Patients** | **Packs per year** | **MXN** | **USD** | **MXN** | **USD** |
| **Hip** | | | | | **$5,605** | **$324.56** |
| Alendronic acid 10 mg tablets (pack of 30) | 6.6% | 12.175 | $353.0 | $20.44 |  |  |
| Alendronic acid 70 mg tablets (pack of 4) | 1.1% | 13.045 | $295.00 | $17.08 |  |  |
| Risedronic acid 5 mg pills (pack of 28) | 0.5% | 13.045 | $1,083.50 | $62.74 |  |  |
| Risedronic acid 35 mg pills (pack of 4) | 8.3% | 13.045 | $1,124.50 | $65.11 |  |  |
| Zoledronic acid 5 mg vial (pack of 1) | 0.1% | 13.045 | $15,909.00^a^ | $921.15 |  |  |
| Denosumab 60 mg prefilled syrange (pack of 1) | 4.8% | 2.000 | $9,299.00 | $538.42 |  |  |
| Teriparatide 250 µg prefilled pen (pack of 1)^b^ | 1.2% | 13.045 | $9,545.00 | $552.67 |  |  |
| Calcium carbonate / vitamin D3 tablets (pack of 30) | 31.8% | 24.350 | $208.00 | $12.04 |  |  |
| **Vertebral^c^** | | | | | **$2,256** | **$130.63** |
| Alendronic acid 10 mg tablets (pack of 30) | 3.1% | 12.175 | $353.0 | $20.44 |  |  |
| Alendronic acid 70 mg tablets (pack of 4) | 0.5% | 13.045 | $295.00 | $17.08 |  |  |
| Risedronic acid 5 mg pills (pack of 28) | 0.2% | 13.045 | $1,083.50 | $62.74 |  |  |
| Risedronic acid 35 mg pills (pack of 4) | 3.8% | 13.045 | $1,124.50 | $65.11 |  |  |
| Zoledronic acid 5 mg vial (pack of 1) | 0.1% | 13.045 | $15,909.00^a^ | $921.15 |  |  |
| Denosumab 60 mg prefilled syrange (pack of 1) | 2.2% | 2.000 | $9,299.00 | $538.42 |  |  |
| Teriparatide 250 µg prefilled pen^b^ (pack of 1) | 0.6% | 13.045 | $9,545.00 | $552.67 |  |  |
| Calcium carbonate / vitamin D3 tablets (pack of 30) | 7.9% | 24.350 | $208.00 | $12.04 |  |  |

**Table S19** Estimate of the cost per event due to drugs by type of fracture: private sector (cont.)

| **Type of fracture and drug or supplementation** | **Drugs** | | **Unit cost per pack** | | **Weighted cost per event** | |
| --- | --- | --- | --- | --- | --- | --- |
|  | **% Patients** | **Packs per year** | **MXN** | **USD** | **MXN** | **USD** |
| **Distal forearm / Proximal forearm** | | | | | **$379** | **$21.94** |
| Alendronic acid 10 mg tablets (pack of 30) | 0.5% | 12.175 | $353.0 | $20.44 |  |  |
| Alendronic acid 70 mg tablets (pack of 4) | 0.1% | 13.045 | $295.00 | $17.08 |  |  |
| Risedronic acid 5 mg pills (pack of 28) | 0.0% | 13.045 | $1,083.50 | $62.74 |  |  |
| Risedronic acid 35 mg pills (pack of 4) | 0.7% | 13.045 | $1,124.50 | $65.11 |  |  |
| Zoledronic acid 5 mg vial (pack of 1) | 0.0% | 13.045 | $15,909.00^a^ | $921.15 |  |  |
| Denosumab 60 mg prefilled syrange (pack of 1) | 0.4% | 2.000 | $9,299.00 | $538.42 |  |  |
| Teriparatide 250 µg prefilled pen (pack of 1)^b^ | 0.1% | 13.045 | $9,545.00 | $552.67 |  |  |
| Calcium carbonate / vitamin D3 tablets (pack of 30) | 1.2% | 24.350 | $208.00 | $12.04 |  |  |

^a^Includes an administration cost of $2,510 MXN (145.33 USD) equivalent to a procedure of an intravenous infusion.

^b^Containing 28 daily doses of 20 mcg.

^c^Applies also to the rest of type of fractures included in the analysis.

Exchange rate: 17.2708 Mexican pesos (MXN) per 1 US Dollar (USD).

Note: The percentage of patients receiving each medicine was assumed to be the same as in the public sector.

Source: Own estimates with data from Biológicos Especializados, Farmacia del Ahorro (online) and Farmalisto (online).

**Table S20** Estimate of the indirect costs per event by type of fracture

| **Description** | **Men 50-59 years** | **Men 60+ years** | **Women 50-59 years** | **Women 60+ years** | **All 50+ years** |
| --- | --- | --- | --- | --- | --- |
| Distribution by sex and age group among those with hip fracture^a^ | 1.86% | 26.80% | 2.93% | 68.41% | 100.00% |
| Patients who were economically active when sustained the fracture^b^ (%) | 94.11% | 53.62% | 56.68% | 23.60% | 33.93% |
| Average daily income in Mexico^c^ |  | | | | $264.89 MXN / $15.34 USD |
| Average sick days due to the fracture^d^ |  | | | | 42 days |
| Indirect costs due to the fracture^e^ |  | | | | $3,775 MXN / $218.55 USD |
|  | | | | | |
| Distribution by sex and age group among those with vertebral fracture^a^ | 8.01% | 23.84% | 11.12% | 57.04% | 100.00% |
| Patients who were economically active when sustained the fracture^b^ (%) | 94.11% | 53.62% | 56.68% | 23.60% | 40.08% |
| Average daily income in Mexico^c^ |  | | | | $264.89 MXN / $15.34 USD |
| Average sick days due to the fracture^d^ |  | | | | 20 days |
| Indirect costs due to the fracture^e^ |  | | | | $2,123 MXN / $122.95 USD |

**Table S20** Estimate of the indirect costs per event by type of fracture (cont.)

| **Description** | **Men 50-59 years** | **Men 60+ years** | **Women 50-59 years** | **Women 60+ years** | **All 50+ years** |
| --- | --- | --- | --- | --- | --- |
| Distribution by sex and age group among those with proximal humerus fracture^a^ | 5.40% | 16.10% | 11.22% | 67.28% | 100.00% |
| Patients who were economically active when sustained the fracture^b^ (%) | 94.11% | 53.62% | 56.68% | 23.60% | 35.95% |
| Average daily income in Mexico^c^ |  | | | | $264.89 MXN / $15.34 USD |
| Average sick days due to the fracture^d^ |  | | | | 12 days |
| Indirect costs due to the fracture^e^ |  | | | | $1,143 MXN / $66.16 USD |
|  | | | | | |
| Distribution by sex and age group among those with distal forearm fracture^a^ | 5.50% | 7.60% | 26.28% | 60.62% | 100.00% |
| Patients who were economically active when sustained the fracture^b^ (%) | 94.11% | 53.62% | 56.68% | 23.60% | 38.45% |
| Average daily income in Mexico^c^ |  | | | | $264.89 MXN / $15.34 USD |
| Average sick days due to the fracture^d^ |  | | | | 12 days |
| Indirect costs due to the fracture^e^ |  | | | | $1,222 MXN / $70.77 USD |

**Table S20** Estimate of the indirect costs per event by type of fracture (cont.)

| **Description** | **Men 50-59 years** | **Men 60+ years** | **Women 50-59 years** | **Women 60+ years** | **All 50+ years** |
| --- | --- | --- | --- | --- | --- |
| Distribution by sex and age group among those with lumbar spine, pelvis fracture^a^ | 5.91% | 21.89% | 6.35% | 65.84% | 100.00% |
| Patients who were economically active when sustained the fracture^b^ (%) | 94.11% | 53.62% | 56.68% | 23.60% | 36.44% |
| Average daily income in Mexico^c^ |  | | | | $264.89 MXN / $15.34 USD |
| Average sick days due to the fracture^d^ |  | | | | 12 days |
| Indirect costs due to the fracture^e^ |  | | | | $1,158 MXN / $67.07 USD |
|  | | | | | |
| Distribution by sex and age group among those with thorax, lumbar zone, pelvis fracture^a^ | 10.79% | 29.96% | 8.31% | 50.93% | 100.00% |
| Patients who were economically active when sustained the fracture^b^ (%) | 94.11% | 53.62% | 56.68% | 23.60% | 42.96% |
| Average daily income in Mexico^c^ |  | | | | $264.89 MXN / $15.34 USD |
| Average sick days due to the fracture^d^ |  | | | | 12 days |
| Indirect costs due to the fracture^e^ |  | | | | $1,365 MXN / $79.06 USD |

**Table S20** Estimate of the indirect costs per event by type of fracture (cont.)

| **Description** | **Men 50-59 years** | **Men 60+ years** | **Women 50-59 years** | **Women 60+ years** | **All 50+ years** |
| --- | --- | --- | --- | --- | --- |
| Distribution by sex and age group among those with shoulder, upper arm fracture^a^ | 12.64% | 20.55% | 11.81% | 55.00% | 100.00% |
| Patients who were economically active when sustained the fracture^b^ (%) | 94.11% | 53.62% | 56.68% | 23.60% | 42.59% |
| Average daily income in Mexico^c^ |  | | | | $264.89 MXN / $15.34 USD |
| Average sick days due to the fracture^d^ |  | | | | 12 days |
| Indirect costs due to the fracture^e^ |  | | | | $1,354 MXN / $78.39 USD |
|  | | | | | |
| Distribution by sex and age group among those with proximal forearm fracture^a^ | 7.28% | 13.19% | 23.94% | 55.58% | 100.00% |
| Patients who were economically active when sustained the fracture^b^ (%) | 94.11% | 53.62% | 56.68% | 23.60% | 40.62% |
| Average daily income in Mexico^c^ |  | | | | $264.89 MXN / $15.34 USD |
| Average sick days due to the fracture^d^ |  | | | | 12 days |
| Indirect costs due to the fracture^e^ |  | | | | $1,291 MXN / $74.75 USD |

**Table S20** Estimate of the indirect costs per event by type of fracture (cont.)

| **Description** | **Men 50-59 years** | **Men 60+ years** | **Women 50-59 years** | **Women 60+ years** | **All 50+ years** |
| --- | --- | --- | --- | --- | --- |
| Distribution by sex and age group among those with other femoral fracture^a^ | 4.25% | 24.38% | 6.02% | 65.36% | 100.00% |
| Patients who were economically active when sustained the fracture^b^ (%) | 94.11% | 53.62% | 56.68% | 23.60% | 35.90% |
| Average daily income in Mexico^c^ |  | | | | $264.89 MXN / $15.34 USD |
| Average sick days due to the fracture^d^ |  | | | | 12 days |
| Indirect costs due to the fracture^e^ |  | | | | $1,141 MXN / $66.08 USD |
|  | | | | | |
| Distribution by sex and age group among those with tibia and fibula fracture^a^ | Not apply | Not apply | 45.33% | 54.67% | 100.00% |
| Patients who were economically active when sustained the fracture^b^ (%) | Not apply | Not apply | 56.68% | 23.60% | 38.59% |
| Average daily income in Mexico^c^ |  | | | | $264.89 MXN / $15.34 USD |
| Average sick days due to the fracture^d^ |  | | | | 12 days |
| Indirect costs due to the fracture^e^ |  | | | | $1,227 MXN / $71.03 USD |

^a^Derived from Tables S21 and S22 (see next). ^b^Assumed to be equal to the national percentage of occupation by sex and age-group from references [15; 24].

^c^Derived from Reference [24]. It corresponds to the weighted monthly income in individuals 50+ years divided by 30.4375 ^d^From Borgstrom et al. [2] ^e^Product of the three previous parameters (i.e. % economical active in all 50+years, average daily income and average sick days)

**Table S21** Estimated number of fragility fractures in men 50+ years

| **Type of fragility fracture** | **Category of initial care** | **Age groups** | | | | | |
| --- | --- | --- | --- | --- | --- | --- | --- |
|  |  | **50-59 years** | **60-69 years** | **70-79 years** | **80-89 years** | **90+ years** | **50+ years** |
| **Hip** | **All cases** | **1,004** | **2,250** | **4,575** | **5,396** | **2,207** | **15,431** |
|  | **Hospital discharges** | **685** | **1,435** | **3,220** | **3,655** | **1,460** | **10,455** |
|  | Public sector | 510 | 1,069 | 2,399 | 2,723 | 1,088 | 7,789 |
|  | Private sector | 175 | 366 | 821 | 932 | 372 | 2,666 |
|  | **Emergency room visit** | **319** | **815** | **1,354** | **1,741** | **747** | **4,976** |
|  | Public sector | 237 | 607 | 1,009 | 1,297 | 556 | 3,707 |
|  | Private sector | 81 | 208 | 345 | 444 | 190 | 1,269 |
|  | | | | | | | |
| **Vertebral** | **All cases** | **3,322** | **3,016** | **3,975** | **2,401** | **491** | **13,204** |
|  | **Hospital discharges** | **121** | **162** | **109** | **46** | **5** | **444** |
|  | Public sector | 90 | 121 | 81 | 34 | 4 | 331 |
|  | Private sector | 31 | 41 | 28 | 12 | 1 | 113 |
|  | **Emergency room visit** | **105** | **81** | **75** | **39** | **3** | **305** |
|  | Public sector | 78 | 61 | 56 | 29 | 3 | 227 |
|  | Private sector | 27 | 21 | 19 | 10 | 1 | 78 |
|  | **Specialty visit** | **3,095** | **2,772** | **3,790** | **2,315** | **482** | **12,455** |
|  | Public sector | 2,306 | 2,065 | 2,824 | 1,725 | 359 | 9,279 |
|  | Private sector | 789 | 707 | 967 | 590 | 123 | 3,176 |

**Table S21** Estimated number of fragility fractures in men 50+ years (cont.)

| **Type of fragility fracture** | **Category of initial care** | **Age groups** | | | | | |
| --- | --- | --- | --- | --- | --- | --- | --- |
|  |  | **50-59 years** | **60-69 years** | **70-79 years** | **80-89 years** | **90+ years** | **50+ years** |
| **Proximal humerus** | **All cases** | **982** | **1,103** | **1,133** | **455** | **239** | **3,913** |
|  | **Hospital discharges** | **497** | **443** | **440** | **188** | **77** | **1,646** |
|  | Public sector | 371 | 330 | 328 | 140 | 58 | 1,226 |
|  | Private sector | 127 | 113 | 112 | 48 | 20 | 420 |
|  | **Emergency room visit** | **485** | **661** | **693** | **267** | **162** | **2,267** |
|  | Public sector | 361 | 492 | 516 | 199 | 120 | 1,689 |
|  | Private sector | 124 | 168 | 177 | 68 | 41 | 578 |
|  | | | | | | | |
| **Distal forearm** | **All cases** | **1,622** | **1,173** | **626** | **367** | **75** | **3,863** |
|  | **Hospital discharges** | **642** | **366** | **218** | **90** | **8** | **1,324** |
|  | Public sector | 478 | 273 | 162 | 67 | 6 | 987 |
|  | Private sector | 164 | 93 | 56 | 23 | 2 | 338 |
|  | **Emergency room visit** | **980** | **806** | **408** | **277** | **67** | **2,539** |
|  | Public sector | 730 | 601 | 304 | 206 | 50 | 1,891 |
|  | Private sector | 250 | 206 | 104 | 71 | 17 | 647 |

**Table S21** Estimated number of fragility fractures in men 50+ years (cont.)

| **Type of fragility fracture** | **Category of initial care** | **Age groups** | | | | | |
| --- | --- | --- | --- | --- | --- | --- | --- |
|  |  | **50-59 years** | **60-69 years** | **70-79 years** | **80-89 years** | **90+ years** | **50+ years** |
| **Lumbar spine, pelvis** | **All cases** | **81** | **74** | **91** | **101** | **34** | **381** |
|  | **Hospital discharges** | **44** | **38** | **40** | **35** | **11** | **167** |
|  | Public sector | 33 | 28 | 30 | 26 | 8 | 125 |
|  | Private sector | 11 | 10 | 10 | 9 | 3 | 43 |
|  | **Emergency room visit** | **37** | **36** | **51** | **67** | **23** | **213** |
|  | Public sector | 28 | 27 | 38 | 50 | 17 | 159 |
|  | Private sector | 9 | 9 | 13 | 17 | 6 | 54 |
|  | | | | | | | |
| **Thorax, lumbar zone, pelvis** | **All cases** | **731** | **871** | **656** | **399** | **103** | **2,761** |
|  | **Hospital discharges** | **155** | **225** | **168** | **97** | **22** | **669** |
|  | Public sector | 116 | 168 | 125 | 73 | 17 | 498 |
|  | Private sector | 40 | 58 | 43 | 25 | 6 | 171 |
|  | **Emergency room visit** | **576** | **646** | **488** | **302** | **81** | **2,092** |
|  | Public sector | 429 | 481 | 364 | 225 | 60 | 1,558 |
|  | Private sector | 147 | 165 | 124 | 77 | 21 | 533 |

**Table S21** Estimated number of fragility fractures in men 50+ years (cont.)

| **Type of fragility fracture** | **Category of initial care** | **Age groups** | | | | | |
| --- | --- | --- | --- | --- | --- | --- | --- |
|  |  | **50-59 years** | **60-69 years** | **70-79 years** | **80-89 years** | **90+ years** | **50+ years** |
| **Shoulder, upper arm** | **All cases** | **1,333** | **1,016** | **671** | **393** | **88** | **3,501** |
|  | **Hospital discharges** | **392** | **270** | **153** | **86** | **19** | **920** |
|  | Public sector | 292 | 201 | 114 | 64 | 14 | 686 |
|  | Private sector | 100 | 69 | 39 | 22 | 5 | 235 |
|  | **Emergency room visit** | **941** | **746** | **518** | **306** | **69** | **2,580** |
|  | Public sector | 701 | 556 | 386 | 228 | 52 | 1,922 |
|  | Private sector | 240 | 190 | 132 | 78 | 18 | 658 |
|  | | | | | | | |
| **Proximal forearm** | **All cases** | **2,534** | **2,135** | **1,297** | **945** | **211** | **7,122** |
|  | **Hospital discharges** | **1,001** | **750** | **391** | **261** | **30** | **2,432** |
|  | Public sector | 746 | 558 | 291 | 195 | 22 | 1,812 |
|  | Private sector | 255 | 191 | 100 | 67 | 8 | 620 |
|  | **Emergency room visit** | **1,533** | **1,385** | **906** | **684** | **182** | **4,690** |
|  | Public sector | 1,142 | 1,032 | 675 | 509 | 135 | 3,494 |
|  | Private sector | 391 | 353 | 231 | 174 | 46 | 1,196 |

**Table S21** Estimated number of fragility fractures in men 50+ years (cont.)

| **Type of fragility fracture** | **Category of initial care** | **Age groups** | | | | | |
| --- | --- | --- | --- | --- | --- | --- | --- |
|  |  | **50-59 years** | **60-69 years** | **70-79 years** | **80-89 years** | **90+ years** | **50+ years** |
| **Other femoral** | **All cases** | **846** | **950** | **1,758** | **1,630** | **521** | **5,706** |
|  | **Hospital discharges** | **555** | **647** | **995** | **935** | **336** | **3,468** |
|  | Public sector | 413 | 482 | 741 | 696 | 250 | 2,583 |
|  | Private sector | 141 | 165 | 254 | 238 | 86 | 884 |
|  | **Emergency room visit** | **291** | **303** | **763** | **695** | **185** | **2,238** |
|  | Public sector | 217 | 226 | 569 | 518 | 138 | 1,667 |
|  | Private sector | 74 | 77 | 195 | 177 | 47 | 571 |

Note: Fractures in tibia and fibula occurred in men were not considered for the analysis.

**Table S22** Estimated number of fragility fractures in women 50+ years

| **Type of fragility fracture** | **Category of initial care** | **Age groups** | | | | | |
| --- | --- | --- | --- | --- | --- | --- | --- |
|  |  | **50-59 years** | **60-69 years** | **70-79 years** | **80-89 years** | **90+ years** | **50+ years** |
| **Hip** | **All cases** | **1,579** | **5,631** | **11,888** | **14,421** | **4,892** | **38,411** |
|  | **Hospital discharges** | **1,001** | **3,554** | **7,849** | **9,965** | **3,144** | **25,513** |
|  | Public sector | 746 | 2,648 | 5,847 | 7,424 | 2,343 | 19,008 |
|  | Private sector | 255 | 906 | 2,001 | 2,541 | 802 | 6,506 |
|  | **Emergency room visit** | **578** | **2,077** | **4,039** | **4,456** | **1,748** | **12,898** |
|  | Public sector | 430 | 1,547 | 3,009 | 3,320 | 1,302 | 9,609 |
|  | Private sector | 147 | 530 | 1,030 | 1,136 | 446 | 3,289 |
|  | | | | | | | |
| **Vertebral** | **All cases** | **4,609** | **8,101** | **9,890** | **4,835** | **820** | **28,255** |
|  | **Hospital discharges** | **167** | **233** | **213** | **77** | **10** | **700** |
|  | Public sector | 125 | 174 | 159 | 57 | 7 | 521 |
|  | Private sector | 43 | 59 | 54 | 20 | 2 | 178 |
|  | **Emergency room visit** | **179** | **247** | **148** | **139** | **0** | **713** |
|  | Public sector | 133 | 184 | 111 | 103 | 0 | 531 |
|  | Private sector | 46 | 63 | 38 | 35 | 0 | 182 |
|  | **Specialty visit** | **4,262** | **7,621** | **9,529** | **4,619** | **811** | **26,843** |
|  | Public sector | 3,175 | 5,678 | 7,099 | 3,441 | 604 | 19,998 |
|  | Private sector | 1,087 | 1,943 | 2,430 | 1,178 | 207 | 6,845 |

**Table S22** Estimated number of fragility fractures in women 50+ years (cont.)

| **Type of fragility fracture** | **Category of initial care** | **Age groups** | | | | | |
| --- | --- | --- | --- | --- | --- | --- | --- |
|  |  | **50-59 years** | **60-69 years** | **70-79 years** | **80-89 years** | **90+ years** | **50+ years** |
| **Proximal humerus** | **All cases** | **2,043** | **4,507** | **4,794** | **2,321** | **626** | **14,291** |
|  | **Hospital discharges** | **854** | **1,593** | **1,604** | **808** | **148** | **5,007** |
|  | Public sector | 636 | 1,187 | 1,195 | 602 | 110 | 3,730 |
|  | Private sector | 218 | 406 | 409 | 206 | 38 | 1,277 |
|  | **Emergency room visit** | **1,189** | **2,914** | **3,190** | **1,514** | **478** | **9,284** |
|  | Public sector | 886 | 2,171 | 2,376 | 1,128 | 356 | 6,917 |
|  | Private sector | 303 | 743 | 813 | 386 | 122 | 2,368 |
|  | | | | | | | |
| **Distal forearm** | **All cases** | **7,751** | **8,148** | **6,193** | **3,046** | **492** | **25,630** |
|  | **Hospital discharges** | **1,883** | **2,257** | **1,592** | **825** | **120** | **6,677** |
|  | Public sector | 1,403 | 1,681 | 1,186 | 615 | 89 | 4,974 |
|  | Private sector | 480 | 575 | 406 | 210 | 31 | 1,703 |
|  | **Emergency room visit** | **5,868** | **5,891** | **4,602** | **2,221** | **372** | **18,953** |
|  | Public sector | 4,372 | 4,389 | 3,428 | 1,654 | 277 | 14,120 |
|  | Private sector | 1,496 | 1,502 | 1,173 | 566 | 95 | 4,833 |

**Table S22** Estimated number of fragility fractures in women 50+ years (cont.)

| **Type of fragility fracture** | **Category of initial care** | **Age groups** | | | | | |
| --- | --- | --- | --- | --- | --- | --- | --- |
|  |  | **50-59 years** | **60-69 years** | **70-79 years** | **80-89 years** | **90+ years** | **50+ years** |
| **Lumbar spine, pelvis** | **All cases** | **87** | **191** | **246** | **351** | **113** | **988** |
|  | **Hospital discharges** | **42** | **49** | **91** | **97** | **22** | **301** |
|  | Public sector | 31 | 37 | 68 | 72 | 16 | 224 |
|  | Private sector | 11 | 13 | 23 | 25 | 6 | 77 |
|  | **Emergency room visit** | **45** | **141** | **155** | **254** | **91** | **687** |
|  | Public sector | 33 | 105 | 116 | 189 | 68 | 512 |
|  | Private sector | 11 | 36 | 40 | 65 | 23 | 175 |
|  | | | | | | | |
| **Thorax, lumbar zone, pelvis** | **All cases** | **563** | **714** | **1,390** | **1,147** | **199** | **4,013** |
|  | **Hospital discharges** | **77** | **118** | **141** | **126** | **43** | **505** |
|  | Public sector | 57 | 88 | 105 | 94 | 32 | 376 |
|  | Private sector | 20 | 30 | 36 | 32 | 11 | 129 |
|  | **Emergency room visit** | **486** | **596** | **1,249** | **1,020** | **156** | **3,508** |
|  | Public sector | 362 | 444 | 930 | 760 | 116 | 2,613 |
|  | Private sector | 124 | 152 | 318 | 260 | 40 | 895 |

**Table S22** Estimated number of fragility fractures in women 50+ years (cont.)

| **Type of fragility fracture** | **Category of initial care** | **Age groups** | | | | | |
| --- | --- | --- | --- | --- | --- | --- | --- |
|  |  | **50-59 years** | **60-69 years** | **70-79 years** | **80-89 years** | **90+ years** | **50+ years** |
| **Shoulder, upper arm** | **All cases** | **1,245** | **2,082** | **1,998** | **1,399** | **320** | **7,045** |
|  | **Hospital discharges** | **336** | **515** | **466** | **266** | **63** | **1,646** |
|  | Public sector | 250 | 384 | 347 | 198 | 47 | 1,226 |
|  | Private sector | 86 | 131 | 119 | 68 | 16 | 420 |
|  | **Emergency room visit** | **909** | **1,567** | **1,532** | **1,133** | **257** | **5,399** |
|  | Public sector | 677 | 1,168 | 1,141 | 844 | 192 | 4,022 |
|  | Private sector | 232 | 400 | 391 | 289 | 66 | 1,377 |
|  | | | | | | | |
| **Proximal forearm** | **All cases** | **8,328** | **8,599** | **6,891** | **3,160** | **682** | **27,661** |
|  | **Hospital discharges** | **2,437** | **2,681** | **2,019** | **898** | **157** | **8,192** |
|  | Public sector | 1,815 | 1,997 | 1,504 | 669 | 117 | 6,103 |
|  | Private sector | 621 | 684 | 515 | 229 | 40 | 2,089 |
|  | **Emergency room visit** | **5,891** | **5,918** | **4,872** | **2,262** | **525** | **19,469** |
|  | Public sector | 4,389 | 4,409 | 3,630 | 1,685 | 391 | 14,504 |
|  | Private sector | 1,502 | 1,509 | 1,242 | 577 | 134 | 4,965 |

**Table S22** Estimated number of fragility fractures in women 50+ years (cont.)

| **Type of fragility fracture** | **Category of initial care** | **Age groups** | | | | | |
| --- | --- | --- | --- | --- | --- | --- | --- |
|  |  | **50-59 years** | **60-69 years** | **70-79 years** | **80-89 years** | **90+ years** | **50+ years** |
| **Other femoral** | **All cases** | **1,200** | **2,861** | **4,001** | **4,671** | **1,493** | **14,227** |
|  | **Hospital discharges** | **741** | **1,860** | **2,490** | **2,850** | **859** | **8,800** |
|  | Public sector | 552 | 1,386 | 1,855 | 2,123 | 640 | 6,556 |
|  | Private sector | 189 | 474 | 635 | 727 | 219 | 2,244 |
|  | **Emergency room visit** | **459** | **1,001** | **1,511** | **1,821** | **634** | **5,427** |
|  | Public sector | 342 | 745 | 1,126 | 1,357 | 473 | 4,043 |
|  | Private sector | 117 | 255 | 385 | 464 | 162 | 1,384 |
|  | | | | | | | |
| **Tibia and fibula** | **All cases** | **5,819** | **4,001** | **1,859** | **1,005** | **154** | **12,838** |
|  | **Hospital discharges** | **2,249** | **1,707** | **792** | **496** | **67** | **5,311** |
|  | Public sector | 1,675 | 1,272 | 590 | 370 | 50 | 3,957 |
|  | Private sector | 573 | 435 | 202 | 127 | 17 | 1,354 |
|  | **Emergency room visit** | **3,570** | **2,294** | **1,067** | **509** | **87** | **7,527** |
|  | Public sector | 2,660 | 1,709 | 795 | 379 | 65 | 5,608 |
|  | Private sector | 910 | 585 | 272 | 130 | 22 | 1,919 |

**Table S23** Estimated number of fragility fractures in all 50+ years

| **Type of fragility fracture** | **Category of initial care** | **Age groups** | | | | | |
| --- | --- | --- | --- | --- | --- | --- | --- |
|  |  | **50-59 years** | **60-69 years** | **70-79 years** | **80-89 years** | **90+ years** | **50+ years** |
| **Hip** | **All cases** | **2,582** | **7,881** | **16,463** | **19,816** | **7,100** | **53,842** |
|  | **Hospital discharges** | **1,686** | **4,989** | **11,069** | **13,619** | **4,605** | **35,968** |
|  | Public sector | 1,256 | 3,717 | 8,246 | 10,146 | 3,431 | 26,796 |
|  | Private sector | 430 | 1,272 | 2,823 | 3,473 | 1,174 | 9,172 |
|  | **Emergency room visit** | **896** | **2,892** | **5,394** | **6,197** | **2,495** | **17,874** |
|  | Public sector | 668 | 2,154 | 4,018 | 4,617 | 1,859 | 13,316 |
|  | Private sector | 229 | 737 | 1,375 | 1,580 | 636 | 4,558 |
|  | | | | | | | |
| **Vertebral** | **All cases** | **7,930** | **11,117** | **13,865** | **7,235** | **1,311** | **41,459** |
|  | **Hospital discharges** | **289** | **395** | **322** | **123** | **15** | **1,144** |
|  | Public sector | 215 | 295 | 240 | 91 | 11 | 852 |
|  | Private sector | 74 | 101 | 82 | 31 | 4 | 292 |
|  | **Emergency room visit** | **284** | **328** | **224** | **178** | **3** | **1,017** |
|  | Public sector | 212 | 244 | 167 | 133 | 3 | 758 |
|  | Private sector | 72 | 84 | 57 | 45 | 1 | 259 |
|  | **Specialty visit** | **7,358** | **10,393** | **13,319** | **6,935** | **1,293** | **39,298** |
|  | Public sector | 5,481 | 7,743 | 9,923 | 5,166 | 963 | 29,277 |
|  | Private sector | 1,876 | 2,650 | 3,396 | 1,768 | 330 | 10,021 |

**Table S23** Estimated number of fragility fractures in all 50+ years (cont.)

| **Type of fragility fracture** | **Category of initial care** | **Age groups** | | | | | |
| --- | --- | --- | --- | --- | --- | --- | --- |
|  |  | **50-59 years** | **60-69 years** | **70-79 years** | **80-89 years** | **90+ years** | **50+ years** |
| **Proximal humerus** | **All cases** | **3,025** | **5,610** | **5,927** | **2,776** | **865** | **18,204** |
|  | **Hospital discharges** | **1,351** | **2,036** | **2,045** | **995** | **225** | **6,652** |
|  | Public sector | 1,007 | 1,517 | 1,523 | 741 | 168 | 4,956 |
|  | Private sector | 345 | 519 | 521 | 254 | 57 | 1,696 |
|  | **Emergency room visit** | **1,674** | **3,574** | **3,882** | **1,781** | **640** | **11,552** |
|  | Public sector | 1,247 | 2,663 | 2,892 | 1,327 | 477 | 8,606 |
|  | Private sector | 427 | 911 | 990 | 454 | 163 | 2,946 |
|  | | | | | | | |
| **Distal forearm** | **All cases** | **9,373** | **9,321** | **6,819** | **3,413** | **567** | **29,493** |
|  | **Hospital discharges** | **2,525** | **2,623** | **1,809** | **916** | **128** | **8,001** |
|  | Public sector | 1,881 | 1,954 | 1,348 | 682 | 95 | 5,961 |
|  | Private sector | 644 | 669 | 461 | 234 | 33 | 2,040 |
|  | **Emergency room visit** | **6,848** | **6,698** | **5,009** | **2,498** | **439** | **21,492** |
|  | Public sector | 5,102 | 4,990 | 3,732 | 1,861 | 327 | 16,011 |
|  | Private sector | 1,746 | 1,708 | 1,277 | 637 | 112 | 5,480 |

**Table S23** Estimated number of fragility fractures in all 50+ years (cont.)

| **Type of fragility fracture** | **Category of initial care** | **Age groups** | | | | | |
| --- | --- | --- | --- | --- | --- | --- | --- |
|  |  | **50-59 years** | **60-69 years** | **70-79 years** | **80-89 years** | **90+ years** | **50+ years** |
| **Lumbar spine, pelvis** | **All cases** | **168** | **264** | **337** | **452** | **147** | **1,369** |
|  | **Hospital discharges** | **86** | **87** | **131** | **132** | **33** | **469** |
|  | Public sector | 64 | 65 | 98 | 98 | 25 | 349 |
|  | Private sector | 22 | 22 | 33 | 34 | 9 | 119 |
|  | **Emergency room visit** | **82** | **177** | **206** | **321** | **114** | **900** |
|  | Public sector | 61 | 132 | 154 | 239 | 85 | 671 |
|  | Private sector | 21 | 45 | 53 | 82 | 29 | 230 |
|  | | | | | | | |
| **Thorax, lumbar zone, pelvis** | **All cases** | **1,294** | **1,586** | **2,046** | **1,546** | **302** | **6,774** |
|  | **Hospital discharges** | **232** | **344** | **309** | **224** | **65** | **1,174** |
|  | Public sector | 173 | 256 | 230 | 167 | 49 | 875 |
|  | Private sector | 59 | 88 | 79 | 57 | 17 | 299 |
|  | **Emergency room visit** | **1,062** | **1,242** | **1,737** | **1,322** | **237** | **5,600** |
|  | Public sector | 791 | 925 | 1,294 | 985 | 176 | 4,172 |
|  | Private sector | 271 | 317 | 443 | 337 | 60 | 1,428 |

**Table S23** Estimated number of fragility fractures in all 50+ years (cont.)

| **Type of fragility fracture** | **Category of initial care** | **Age groups** | | | | | |
| --- | --- | --- | --- | --- | --- | --- | --- |
|  |  | **50-59 years** | **60-69 years** | **70-79 years** | **80-89 years** | **90+ years** | **50+ years** |
| **Shoulder, upper arm** | **All cases** | **2,578** | **3,098** | **2,669** | **1,791** | **409** | **10,545** |
|  | **Hospital discharges** | **728** | **785** | **619** | **352** | **82** | **2,566** |
|  | Public sector | 542 | 585 | 461 | 262 | 61 | 1,912 |
|  | Private sector | 186 | 200 | 158 | 90 | 21 | 654 |
|  | **Emergency room visit** | **1,850** | **2,313** | **2,050** | **1,439** | **327** | **7,979** |
|  | Public sector | 1,378 | 1,723 | 1,527 | 1,072 | 243 | 5,944 |
|  | Private sector | 472 | 590 | 523 | 367 | 83 | 2,035 |
|  | | | | | | | |
| **Proximal forearm** | **All cases** | **10,862** | **10,734** | **8,188** | **4,105** | **894** | **34,783** |
|  | **Hospital discharges** | **3,437** | **3,431** | **2,410** | **1,159** | **187** | **10,624** |
|  | Public sector | 2,561 | 2,556 | 1,795 | 863 | 139 | 7,915 |
|  | Private sector | 877 | 875 | 615 | 296 | 48 | 2,709 |
|  | **Emergency room visit** | **7,424** | **7,303** | **5,778** | **2,946** | **707** | **24,159** |
|  | Public sector | 5,531 | 5,441 | 4,305 | 2,195 | 526 | 17,998 |
|  | Private sector | 1,893 | 1,862 | 1,473 | 751 | 180 | 6,160 |

**Table S23** Estimated number of fragility fractures in all 50+ years (cont.)

| **Type of fragility fracture** | **Category of initial care** | **Age groups** | | | | | |
| --- | --- | --- | --- | --- | --- | --- | --- |
|  |  | **50-59 years** | **60-69 years** | **70-79 years** | **80-89 years** | **90+ years** | **50+ years** |
| **Other femoral** | **All cases** | **2,046** | **3,811** | **5,760** | **6,301** | **2,015** | **19,932** |
|  | **Hospital discharges** | **1,295** | **2,507** | **3,486** | **3,785** | **1,195** | **12,268** |
|  | Public sector | 965 | 1,868 | 2,597 | 2,820 | 890 | 9,140 |
|  | Private sector | 330 | 639 | 889 | 965 | 305 | 3,128 |
|  | **Emergency room visit** | **751** | **1,304** | **2,274** | **2,516** | **820** | **7,664** |
|  | Public sector | 559 | 971 | 1,694 | 1,875 | 611 | 5,710 |
|  | Private sector | 191 | 332 | 580 | 642 | 209 | 1,954 |
|  | | | | | | | |
| **Tibia and fibula^a^** | **All cases** | **5,819** | **4,001** | **1,859** | **1,005** | **154** | **12,838** |
|  | **Hospital discharges** | **2,249** | **1,707** | **792** | **496** | **67** | **5,311** |
|  | Public sector | 1,675 | 1,272 | 590 | 370 | 50 | 3,957 |
|  | Private sector | 573 | 435 | 202 | 127 | 17 | 1,354 |
|  | **Emergency room visit** | **3,570** | **2,294** | **1,067** | **509** | **87** | **7,527** |
|  | Public sector | 2,660 | 1,709 | 795 | 379 | 65 | 5,608 |
|  | Private sector | 910 | 585 | 272 | 130 | 22 | 1,919 |

^a^Only fractures occurred in women were considered for the analysis.

**Table S24** Projected costs due to fragility fractures: Mexico 2023 (US Dollars)

| **Section A) Data broken down by main categories of FF** | | | | |
| --- | --- | --- | --- | --- |
| **Description** | **Hip** | **Vertebral** | **MOF** | **All FF** |
| **Direct medical costs** | **$422,222,296** | **$53,655,867** | **$596,674,224** | **$876,426,164** |
| Acute care | $362,303,254 | $15,314,539 | $461,600,674 | $678,799,407 |
| Follow-up | $59,919,042 | $38,341,327 | $135,073,550 | $197,626,757 |
| Rehabilitation | $13,088,957 | $8,054,214 | $32,437,407 | $50,808,624 |
| Specialty visits | $9,753,777 | $5,995,570 | $19,761,722 | $26,557,625 |
| Laboratory test | $22,801,451 | $16,680,940 | $56,049,970 | $84,227,815 |
| Imaging tests | $6,764,994 | $5,253,540 | $15,562,580 | $21,590,314 |
| Drugs^a^ | $7,509,863 | $2,357,063 | $11,261,871 | $14,442,379 |
| **Indirect costs** | **$11,767,450** | **$5,097,394** | **$20,248,286** | **$26,439,459** |
| **Total costs** | **$433,989,746** | **$58,753,261** | **$616,922,511** | **$902,865,623** |
|  | | | | |
| **Section B) Data broken down by health care sector** | | | | |
| **Description** | **Public sector** | **Private sector** | **Both sectors** |  |
| **Direct medical costs** | **$442,990,136** | **$433,436,028** | **$876,426,164** |  |
| Acute care | $349,155,145 | $329,644,262 | $678,799,407 |  |
| Follow-up | $93,834,991 | $103,791,766 | $197,626,757 |  |
| Rehabilitation | $30,909,187 | $19,899,437 | $50,808,624 |  |
| Specialty visits | $17,998,861 | $8,558,764 | $26,557,625 |  |
| Laboratory test | $27,175,971 | $57,051,844 | $84,227,815 |  |
| Imaging tests | $11,825,773 | $9,764,540 | $21,590,314 |  |
| Drugs^a^ | $5,925,198 | $8,517,181 | $14,442,379 |  |
| **Indirect costs** | **$19,697,397** | **$6,742,062** | **$26,439,459** |  |
| **Total costs** | **$462,687,533** | **$440,178,090** | **$902,865,623** |  |

FF: fragility fractures; MOF: major osteoporotic fractures.

^a^Includes pharmacological treatment and supplementation with calcium plus vitamin D.

**Table S25** Projected costs by type of fragility fracture: hip

| **Description** | **Number of FF** | **Cost per event** | | **Costs** | |
| --- | --- | --- | --- | --- | --- |
|  |  | **MXN** | **USD** | **MXN** | **USD** |
| **Direct medical costs** | **53,842** |  |  | **$7,292,116,837** | **$422,222,296** |
| Acute care | 53,842 |  |  | $6,257,267,045 | $362,303,254 |
| Hospital discharge^a^ | 26,796 | $117,933 | $6,828 | $3,160,185,770 | $182,978,540 |
| Hospital discharge^b^ | 9,172 | $333,751 | $19,325 | $3,061,138,336 | $177,243,575 |
| ER visit^a^ | 13,316 | $1,900 | $110 | $25,300,412 | $1,464,924 |
| ER visit^b^ | 4,558 | $2,335 | $135 | $10,642,527 | $616,215 |
| Specialty visit^a^ | NA | NA | NA | NA | NA |
| Specialty visit^b^ | NA | NA | NA | NA | NA |
| Follow-up | 53,842 |  |  | $1,034,849,792 | $59,919,042 |
| Rehabilitation | 53,842 |  |  | $226,056,751 | $13,088,957 |
| Public sector | 40,112 | $3,400 | $197 | $136,384,269 | $7,896,812 |
| Private sector | 13,730 | $6,531 | $378 | $89,672,482 | $5,192,144 |
| Specialty visits | 53,842 |  |  | $168,455,539 | $9,753,777 |
| Public sector | 40,112 | $2,786 | $161 | $111,757,242 | $6,470,878 |
| Private sector | 13,730 | $4,130 | $239 | $56,698,297 | $3,282,899 |
| Laboratory tests | 53,842 |  |  | $393,799,308 | $22,801,451 |
| Public sector | 40,112 | $3,225 | $187 | $129,360,869 | $7,490,149 |
| Private sector | 13,730 | $19,260 | $1,115 | $264,438,439 | $15,311,302 |
| Imaging tests | 53,842 |  |  | $116,836,852 | $6,764,994 |
| Public sector | 40,112 | $1,518 | $88 | $60,898,119 | $3,526,074 |
| Private sector | 13,730 | $4,074 | $236 | $55,938,733 | $3,238,920 |
| Drugs^c^ | 53,842 |  |  | $129,701,341 | $7,509,863 |
| Public sector | 40,112 | $1,315 | $76 | $52,741,575 | $3,053,800 |
| Private sector | 13,730 | $5,605 | $325 | $76,959,766 | $4,456,063 |
| **Indirect costs** | **53,842** |  |  | **$203,233,275** | **$11,767,450** |
| Public sector | 40,112 | $3,775 | $219 | $151,408,790 | $8,766,750 |
| Private sector | 13,730 | $3,775 | $219 | $51,824,485 | $3,000,700 |
| **Total costs** | **53,842** |  |  | **$7,495,350,112** | **$433,989,746** |

FF: fragility fractures; NA: not applicable. Exchange rate: 17.2708 Mexican pesos (MXN) per 1 US Dollar (USD).

^a^Public sector.

^b^Private sector.

^c^Includes pharmacological treatment and supplementation with calcium plus vitamin D.

**Table S26** Projected costs by type of fragility fracture: vertebral

| **Description** | **Number of FF** | **Cost per event** | | **Costs** | |
| --- | --- | --- | --- | --- | --- |
|  |  | **MXN** | **USD** | **MXN** | **USD** |
| **Direct medical costs** | **41,459** |  |  | **$926,679,743** | **$53,655,867** |
| Acute care | 41,459 |  |  | $264,494,346 | $15,314,539 |
| Hospital discharge^a^ | 852 | $62,546 | $3,622 | $53,294,893 | $3,085,838 |
| Hospital discharge^b^ | 292 | $177,006 | $10,249 | $51,624,510 | $2,989,121 |
| ER visit^a^ | 758 | $1,900 | $110 | $1,440,073 | $83,382 |
| ER visit^b^ | 259 | $2,335 | $135 | $605,761 | $35,074 |
| Specialty visit^a^ | 29,277 | $3,877 | $224 | $113,506,919 | $6,572,186 |
| Specialty visit^b^ | 10,021 | $4,393 | $254 | $44,022,190 | $2,548,938 |
| Follow-up | 41,459 |  |  | $662,185,397 | $38,341,327 |
| Rehabilitation | 41,459 |  |  | $139,102,727 | $8,054,214 |
| Public sector | 30,887 | $2,707 | $157 | $83,599,379 | $4,840,504 |
| Private sector | 10,572 | $5,250 | $304 | $55,503,347 | $3,213,710 |
| Specialty visits | 41,459 |  |  | $103,548,295 | $5,995,570 |
| Public sector | 30,887 | $2,248 | $130 | $69,441,515 | $4,020,747 |
| Private sector | 10,572 | $3,226 | $187 | $34,106,780 | $1,974,823 |
| Laboratory tests | 41,459 |  |  | $288,093,172 | $16,680,940 |
| Public sector | 30,887 | $2,916 | $169 | $90,056,014 | $5,214,351 |
| Private sector | 10,572 | $18,732 | $1,085 | $198,037,158 | $11,466,589 |
| Imaging tests | 41,459 |  |  | $90,732,831 | $5,253,540 |
| Public sector | 30,887 | $1,567 | $91 | $48,401,847 | $2,802,525 |
| Private sector | 10,572 | $4,004 | $232 | $42,330,985 | $2,451,015 |
| Drugs^c^ | 41,459 |  |  | $40,708,372 | $2,357,063 |
| Public sector | 30,887 | $546 | $32 | $16,857,020 | $976,042 |
| Private sector | 10,572 | $2,256 | $131 | $23,851,352 | $1,381,022 |
| **Indirect costs** | **41,459** |  |  | **$88,036,073** | **$5,097,394** |
| Public sector | 30,887 | $2,123 | $123 | $65,586,875 | $3,797,559 |
| Private sector | 10,572 | $2,123 | $123 | $22,449,199 | $1,299,835 |
| **Total costs** | **41,459** |  |  | **$1,014,715,816** | **$58,753,261** |

FF: fragility fractures; NA: not applicable. Exchange rate: 17.2708 Mexican pesos (MXN) per 1 US Dollar (USD).

^a^Public sector.

^b^Private sector.

^c^Includes pharmacological treatment and supplementation with calcium plus vitamin D.

**Table S27** Projected costs by type of fragility fracture: proximal humerus

| **Description** | **Number of FF** | **Cost per event** | | **Costs** | |
| --- | --- | --- | --- | --- | --- |
|  |  | **MXN** | **USD** | **MXN** | **USD** |
| **Direct medical costs** | **18,204** |  |  | **$868,081,622** | **$50,262,965** |
| Acute care | 18,204 |  |  | $626,817,265 | $36,293,470 |
| Hospital discharge^a^ | 4,956 | $61,863 | $3,582 | $306,598,583 | $17,752,425 |
| Hospital discharge^b^ | 1,696 | $175,073 | $10,137 | $296,989,083 | $17,196,024 |
| ER visit^a^ | 8,606 | $1,900 | $110 | $16,351,429 | $946,767 |
| ER visit^b^ | 2,946 | $2,335 | $135 | $6,878,170 | $398,254 |
| Specialty visit^a^ | NA | NA | NA | NA | NA |
| Specialty visit^b^ | NA | NA | NA | NA | NA |
| Follow-up | 18,204 |  |  | $241,264,356 | $13,969,495 |
| Rehabilitation | 18,204 |  |  | $75,052,044 | $4,345,603 |
| Public sector | 13,562 | $3,400 | $197 | $46,111,742 | $2,669,925 |
| Private sector | 4,642 | $6,234 | $361 | $28,940,302 | $1,675,678 |
| Specialty visits | 18,204 |  |  | $24,225,545 | $1,402,688 |
| Public sector | 13,562 | $1,233 | $71 | $16,728,629 | $968,608 |
| Private sector | 4,642 | $1,615 | $94 | $7,496,916 | $434,080 |
| Laboratory tests | 18,204 |  |  | $103,704,926 | $6,004,639 |
| Public sector | 13,562 | $2,433 | $141 | $33,002,051 | $1,910,858 |
| Private sector | 4,642 | $15,231 | $882 | $70,702,874 | $4,093,781 |
| Imaging tests | 18,204 |  |  | $20,407,337 | $1,181,609 |
| Public sector | 13,562 | $917 | $53 | $12,436,685 | $720,099 |
| Private sector | 4,642 | $1,717 | $99 | $7,970,652 | $461,510 |
| Drugs^c^ | 18,204 |  |  | $17,874,504 | $1,034,955 |
| Public sector | 13,562 | $546 | $32 | $7,401,693 | $428,567 |
| Private sector | 4,642 | $2,256 | $131 | $10,472,811 | $606,388 |
| **Indirect costs** | **18,204** |  |  | **$20,801,994** | **$1,204,460** |
| Public sector | 13,562 | $1,143 | $66 | $15,497,485 | $897,323 |
| Private sector | 4,642 | $1,143 | $66 | $5,304,508 | $307,137 |
| **Total costs** | **18,204** |  |  | **$888,883,615** | **$51,467,426** |

FF: fragility fractures; NA: not applicable. Exchange rate: 17.2708 Mexican pesos (MXN) per 1 US Dollar (USD).

^a^Public sector.

^b^Private sector.

^c^Includes pharmacological treatment and supplementation with calcium plus vitamine D.

**Table S28** Projected costs by type of fragility fracture: distal forearm

| **Description** | **Number of FF** | **Cost per event** | | **Costs** | |
| --- | --- | --- | --- | --- | --- |
|  |  | **MXN** | **USD** | **MXN** | **USD** |
| **Direct medical costs** | **29,493** |  |  | **$1,142,562,339** | **$66,155,728** |
| Acute care | 29,493 |  |  | $770,421,130 | $44,608,306 |
| Hospital discharge^a^ | 5,961 | $61,970 | $3,588 | $369,390,002 | $21,388,123 |
| Hospital discharge^b^ | 2,040 | $175,374 | $10,154 | $357,812,477 | $20,717,771 |
| ER visit^a^ | 16,011 | $1,900 | $110 | $30,421,821 | $1,761,460 |
| ER visit^b^ | 5,480 | $2,335 | $135 | $12,796,830 | $740,952 |
| Specialty visit^a^ | NA | NA | NA | NA | NA |
| Specialty visit^b^ | NA | NA | NA | NA | NA |
| Follow-up | 29,493 |  |  | $372,141,209 | $21,547,422 |
| Rehabilitation | 29,493 |  |  | $114,134,506 | $6,608,525 |
| Public sector | 21,972 | $3,117 | $180 | $68,481,368 | $3,965,153 |
| Private sector | 7,521 | $6,070 | $351 | $45,653,139 | $2,643,371 |
| Specialty visits | 29,493 |  |  | $41,551,988 | $2,405,910 |
| Public sector | 21,972 | $1,318 | $76 | $28,954,009 | $1,676,472 |
| Private sector | 7,521 | $1,675 | $97 | $12,597,978 | $729,438 |
| Laboratory tests | 29,493 |  |  | $173,541,541 | $10,048,263 |
| Public sector | 21,972 | $2,564 | $148 | $56,334,779 | $3,261,851 |
| Private sector | 7,521 | $15,585 | $902 | $117,206,762 | $6,786,412 |
| Imaging tests | 29,493 |  |  | $38,039,655 | $2,202,542 |
| Public sector | 21,972 | $960 | $56 | $21,082,789 | $1,220,719 |
| Private sector | 7,521 | $2,255 | $131 | $16,956,867 | $981,823 |
| Drugs^c^ | 29,493 |  |  | $4,873,519 | $282,183 |
| Public sector | 21,972 | $92 | $5 | $2,023,608 | $117,169 |
| Private sector | 7,521 | $379 | $22 | $2,849,911 | $165,013 |
| **Indirect costs** | **29,493** |  |  | **$36,047,397** | **$2,087,187** |
| Public sector | 21,972 | $1,222 | $71 | $26,855,310 | $1,554,955 |
| Private sector | 7,521 | $1,222 | $71 | $9,192,086 | $532,233 |
| **Total costs** | **29,493** |  |  | **$1,178,609,735** | **$68,242,915** |

FF: fragility fractures; NA: not applicable. Exchange rate: 17.2708 Mexican pesos (MXN) per 1 US Dollar (USD).

^a^Public sector.

^b^Private sector.

^c^Includes pharmacological treatment and supplementation with calcium plus vitamin D.

**Table S29** Projected costs by type of fragility fracture: lumbar spine, pelvis

| **Description** | **Number of FF** | **Cost per event** | | **Costs** | |
| --- | --- | --- | --- | --- | --- |
|  |  | **MXN** | **USD** | **MXN** | **USD** |
| **Direct medical costs** | **1,369** |  |  | **$75,600,650** | **$4,377,368** |
| Acute care | 1,369 |  |  | $53,213,143 | $3,081,105 |
| Hospital discharge^a^ | 349 | $74,803 | $4,331 | $26,110,818 | $1,511,848 |
| Hospital discharge^b^ | 119 | $211,692 | $12,257 | $25,292,446 | $1,464,463 |
| ER visit^a^ | 671 | $1,900 | $110 | $1,273,983 | $73,765 |
| ER visit^b^ | 230 | $2,335 | $135 | $535,896 | $31,029 |
| Specialty visit^a^ | NA | NA | NA | NA | NA |
| Specialty visit^b^ | NA | NA | NA | NA | NA |
| Follow-up | 1,369 |  |  | $22,387,507 | $1,296,263 |
| Rehabilitation | 1,369 |  |  | $5,873,942 | $340,108 |
| Public sector | 1,020 | $3,683 | $213 | $3,755,504 | $217,448 |
| Private sector | 349 | $6,070 | $351 | $2,118,438 | $122,660 |
| Specialty visits | 1,369 |  |  | $3,519,381 | $203,776 |
| Public sector | 1,020 | $2,521 | $146 | $2,570,111 | $148,812 |
| Private sector | 349 | $2,720 | $157 | $949,270 | $54,964 |
| Laboratory tests | 1,369 |  |  | $8,888,880 | $514,677 |
| Public sector | 1,020 | $2,934 | $170 | $2,991,180 | $173,193 |
| Private sector | 349 | $16,900 | $979 | $5,897,700 | $341,484 |
| Imaging tests | 1,369 |  |  | $2,761,523 | $159,895 |
| Public sector | 1,020 | $1,604 | $93 | $1,635,895 | $94,720 |
| Private sector | 349 | $3,225 | $187 | $1,125,627 | $65,175 |
| Drugs^c^ | 1,369 |  |  | $1,343,781 | $77,807 |
| Public sector | 1,020 | $546 | $32 | $556,449 | $32,219 |
| Private sector | 349 | $2,256 | $131 | $787,332 | $45,587 |
| **Indirect costs** | **1,369** |  |  | **$1,585,367** | **$91,795** |
| Public sector | 1,020 | $1,158 | $67 | $1,181,098 | $68,387 |
| Private sector | 349 | $1,158 | $67 | $404,268 | $23,408 |
| **Total costs** | **1,369** |  |  | **$77,186,016** | **$4,469,163** |

FF: fragility fractures; NA: not applicable. Exchange rate: 17.2708 Mexican pesos (MXN) per 1 US Dollar (USD).

^a^Public sector.

^b^Private sector.

^c^Includes pharmacological treatment and supplementation with calcium plus vitamin D.

**Table S30** Projected costs by type of fragility fracture: thorax, lumbar zone, pelvis

| **Description** | **Number of FF** | **Cost per event** | | **Costs** | |
| --- | --- | --- | --- | --- | --- |
|  |  | **MXN** | **USD** | **MXN** | **USD** |
| **Direct medical costs** | **6,774** |  |  | **$242,488,707** | **$14,040,386** |
| Acute care | 6,774 |  |  | $176,136,991 | $10,198,543 |
| Hospital discharge^a^ | 875 | $95,765 | $5,545 | $83,750,669 | $4,849,264 |
| Hospital discharge^b^ | 299 | $271,014 | $15,692 | $81,125,732 | $4,697,277 |
| ER visit^a^ | 4,172 | $1,900 | $110 | $7,926,385 | $458,947 |
| ER visit^b^ | 1,428 | $2,335 | $135 | $3,334,206 | $193,054 |
| Specialty visit^a^ | NA | NA | NA | NA | NA |
| Specialty visit^b^ | NA | NA | NA | NA | NA |
| Follow-up | 6,774 |  |  | $66,351,716 | $3,841,844 |
| Rehabilitation | 6,774 |  |  | $8,223,479 | $476,149 |
| Public sector | 5,046 | $1,148 | $66 | $5,794,510 | $335,509 |
| Private sector | 1,727 | $1,406 | $81 | $2,428,969 | $140,640 |
| Specialty visits | 6,774 |  |  | $7,262,668 | $420,517 |
| Public sector | 5,046 | $1,011 | $59 | $5,102,703 | $295,453 |
| Private sector | 1,727 | $1,251 | $72 | $2,159,965 | $125,065 |
| Laboratory tests | 6,774 |  |  | $36,254,779 | $2,099,195 |
| Public sector | 5,046 | $2,303 | $133 | $11,623,388 | $673,008 |
| Private sector | 1,727 | $14,260 | $826 | $24,631,391 | $1,426,187 |
| Imaging tests | 6,774 |  |  | $7,959,846 | $460,885 |
| Public sector | 5,046 | $954 | $55 | $4,812,964 | $278,676 |
| Private sector | 1,727 | $1,822 | $105 | $3,146,883 | $182,208 |
| Drugs^c^ | 6,774 |  |  | $6,650,944 | $385,098 |
| Public sector | 5,046 | $546 | $32 | $2,754,104 | $159,466 |
| Private sector | 1,727 | $2,256 | $131 | $3,896,840 | $225,632 |
| **Indirect costs** | **6,774** |  |  | **$9,248,812** | **$535,517** |
| Public sector | 5,046 | $1,365 | $79 | $6,890,365 | $398,960 |
| Private sector | 1,727 | $1,365 | $79 | $2,358,447 | $136,557 |
| **Total costs** | **6,774** |  |  | **$251,737,519** | **$14,575,904** |

FF: fragility fractures; NA: not applicable. Exchange rate: 17.2708 Mexican pesos (MXN) per 1 US Dollar (USD).

^a^Public sector.

^b^Private sector.

^c^Includes pharmacological treatment and supplementation with calcium plus vitamin D.

**Table S31** Projected costs by type of fragility fracture: shoulder, upper arm

| **Description** | **Number of FF** | **Cost per event** | | **Costs** | |
| --- | --- | --- | --- | --- | --- |
|  |  | **MXN** | **USD** | **MXN** | **USD** |
| **Direct medical costs** | **10,545** |  |  | **$382,242,524** | **$22,132,300** |
| Acute care | 10,545 |  |  | $242,485,039 | $14,040,174 |
| Hospital discharge^a^ | 1,912 | $60,161 | $3,483 | $115,022,676 | $6,659,951 |
| Hospital discharge^b^ | 654 | $170,255 | $9,858 | $111,417,603 | $6,451,213 |
| ER visit^a^ | 5,944 | $1,900 | $110 | $11,293,985 | $653,935 |
| ER visit^b^ | 2,035 | $2,335 | $135 | $4,750,774 | $275,076 |
| Specialty visit^a^ | NA | NA | NA | NA | NA |
| Specialty visit^b^ | NA | NA | NA | NA | NA |
| Follow-up | 10,545 |  |  | $139,757,485 | $8,092,126 |
| Rehabilitation | 10,545 |  |  | $43,475,485 | $2,517,283 |
| Public sector | 7,856 | $3,400 | $197 | $26,711,203 | $1,546,611 |
| Private sector | 2,689 | $6,234 | $361 | $16,764,282 | $970,672 |
| Specialty visits | 10,545 |  |  | $14,033,160 | $812,537 |
| Public sector | 7,856 | $1,233 | $71 | $9,690,412 | $561,086 |
| Private sector | 2,689 | $1,615 | $94 | $4,342,747 | $251,450 |
| Laboratory tests | 10,545 |  |  | $60,073,273 | $3,478,314 |
| Public sector | 7,856 | $2,433 | $141 | $19,117,137 | $1,106,905 |
| Private sector | 2,689 | $15,231 | $882 | $40,956,136 | $2,371,409 |
| Imaging tests | 10,545 |  |  | $11,821,382 | $684,472 |
| Public sector | 7,856 | $917 | $53 | $7,204,213 | $417,133 |
| Private sector | 2,689 | $1,717 | $99 | $4,617,169 | $267,340 |
| Drugs^c^ | 10,545 |  |  | $10,354,185 | $599,520 |
| Public sector | 7,856 | $546 | $32 | $4,287,587 | $248,256 |
| Private sector | 2,689 | $2,256 | $131 | $6,066,597 | $351,263 |
| **Indirect costs** | **10,545** |  |  | **$14,275,949** | **$826,595** |
| Public sector | 7,856 | $1,354 | $78 | $10,635,582 | $615,813 |
| Private sector | 2,689 | $1,354 | $78 | $3,640,367 | $210,782 |
| **Total costs** | **10,545** |  |  | **$396,518,473** | **$22,958,894** |

FF: fragility fractures; NA: not applicable. Exchange rate: 17.2708 Mexican pesos (MXN) per 1 US Dollar (USD).

^a^Public sector.

^b^Private sector.

^c^Includes pharmacological treatment and supplementation with calcium plus vitamin D.

**Table S32** Projected costs by type of fragility fracture: proximal forearm

| **Description** | **Number of FF** | **Cost per event** | | **Costs** | |
| --- | --- | --- | --- | --- | --- |
|  |  | **MXN** | **USD** | **MXN** | **USD** |
| **Direct medical costs** | **34,783** |  |  | **$1,453,065,784** | **$84,134,249** |
| Acute care | 34,783 |  |  | $1,014,180,888 | $58,722,288 |
| Hospital discharge^a^ | 7,915 | $61,970 | $3,588 | $490,486,434 | $28,399,752 |
| Hospital discharge^b^ | 2,709 | $175,374 | $10,154 | $475,113,470 | $27,509,639 |
| ER visit^a^ | 17,998 | $1,900 | $110 | $34,196,394 | $1,980,012 |
| ER visit^b^ | 6,160 | $2,335 | $135 | $14,384,591 | $832,885 |
| Specialty visit^a^ | NA | NA | NA | NA | NA |
| Specialty visit^b^ | NA | NA | NA | NA | NA |
| Follow-up | 34,783 |  |  | $438,884,896 | $25,411,961 |
| Rehabilitation | 34,783 |  |  | $134,604,579 | $7,793,766 |
| Public sector | 25,913 | $3,117 | $180 | $80,763,531 | $4,676,305 |
| Private sector | 8,870 | $6,070 | $351 | $53,841,049 | $3,117,461 |
| Specialty visits | 34,783 |  |  | $49,004,355 | $2,837,411 |
| Public sector | 25,913 | $1,318 | $76 | $34,146,923 | $1,977,148 |
| Private sector | 8,870 | $1,675 | $97 | $14,857,431 | $860,263 |
| Laboratory tests | 34,783 |  |  | $204,666,291 | $11,850,423 |
| Public sector | 25,913 | $2,564 | $148 | $66,438,446 | $3,846,866 |
| Private sector | 8,870 | $15,585 | $902 | $138,227,845 | $8,003,558 |
| Imaging tests | 34,783 |  |  | $44,862,084 | $2,597,568 |
| Public sector | 25,913 | $960 | $56 | $24,863,996 | $1,439,655 |
| Private sector | 8,870 | $2,255 | $131 | $19,998,088 | $1,157,913 |
| Drugs^c^ | 34,783 |  |  | $5,747,587 | $332,792 |
| Public sector | 25,913 | $92 | $5 | $2,386,543 | $138,184 |
| Private sector | 8,870 | $379 | $22 | $3,361,044 | $194,608 |
| **Indirect costs** | **34,783** |  |  | **$44,905,239** | **$2,600,067** |
| Public sector | 25,913 | $1,291 | $75 | $33,454,403 | $1,937,050 |
| Private sector | 8,870 | $1,291 | $75 | $11,450,836 | $663,017 |
| **Total costs** | **34,783** |  |  | **$1,497,971,022** | **$86,734,316** |

FF: fragility fractures; NA: not applicable. Exchange rate: 17.2708 Mexican pesos (MXN) per 1 US Dollar (USD).

^a^Public sector.

^b^Private sector.

^c^Includes pharmacological treatment and supplementation with calcium plus vitamin D.

**Table S33** Projected costs by type of fragility fracture: other femoral

| **Description** | **Number of FF** | **Cost per event** | | **Costs** | |
| --- | --- | --- | --- | --- | --- |
|  |  | **MXN** | **USD** | **MXN** | **USD** |
| **Direct medical costs** | **19,932** |  |  | **$2,009,354,295** | **$116,344,020** |
| Acute care | 19,932 |  |  | $1,741,573,369 | $100,839,183 |
| Hospital discharge^a^ | 9,140 | $95,937 | $5,555 | $876,821,299 | $50,769,003 |
| Hospital discharge^b^ | 3,128 | $271,501 | $15,720 | $849,339,718 | $49,177,787 |
| ER visit^a^ | 5,710 | $1,900 | $110 | $10,848,830 | $628,160 |
| ER visit^b^ | 1,954 | $2,335 | $135 | $4,563,522 | $264,233 |
| Specialty visit^a^ | NA | NA | NA | NA | NA |
| Specialty visit^b^ | NA | NA | NA | NA | NA |
| Follow-up | 19,932 |  |  | $267,780,926 | $15,504,836 |
| Rehabilitation | 19,932 |  |  | $77,135,249 | $4,466,223 |
| Public sector | 14,849 | $3,117 | $180 | $46,281,598 | $2,679,760 |
| Private sector | 5,083 | $6,070 | $351 | $30,853,651 | $1,786,463 |
| Specialty visits | 19,932 |  |  | $28,081,980 | $1,625,980 |
| Public sector | 14,849 | $1,318 | $76 | $19,567,918 | $1,133,006 |
| Private sector | 5,083 | $1,675 | $97 | $8,514,061 | $492,974 |
| Laboratory tests | 19,932 |  |  | $117,284,162 | $6,790,893 |
| Public sector | 14,849 | $2,564 | $148 | $38,072,598 | $2,204,449 |
| Private sector | 5,083 | $15,585 | $902 | $79,211,564 | $4,586,444 |
| Imaging tests | 19,932 |  |  | $25,708,249 | $1,488,538 |
| Public sector | 14,849 | $960 | $56 | $14,248,330 | $824,995 |
| Private sector | 5,083 | $2,255 | $131 | $11,459,918 | $663,543 |
| Drugs^c^ | 19,932 |  |  | $19,571,286 | $1,133,201 |
| Public sector | 14,849 | $546 | $32 | $8,104,318 | $469,250 |
| Private sector | 5,083 | $2,256 | $131 | $11,466,969 | $663,951 |
| **Indirect costs** | **19,932** |  |  | **$22,747,577** | **$1,317,112** |
| Public sector | 14,849 | $1,141 | $66 | $16,946,944 | $981,248 |
| Private sector | 5,083 | $1,141 | $66 | $5,800,632 | $335,864 |
| **Total costs** | **19,932** |  |  | **$2,032,101,871** | **$117,661,132** |

FF: fragility fractures; NA: not applicable. Exchange rate: 17.2708 Mexican pesos (MXN) per 1 US Dollar (USD).

^a^Public sector.

^b^Private sector.

^c^Includes pharmacological treatment and supplementation with calcium plus vitamin D.

**Table S34** Projected costs by type of fragility fracture: tibia and fibula

| **Description** | **Number of FF** | **Cost per event** | | **Costs** | |
| --- | --- | --- | --- | --- | --- |
|  |  | **MXN** | **USD** | **MXN** | **USD** |
| **Direct medical costs** | **12,838** |  |  | **$744,388,496** | **$43,100,985** |
| Acute care | 12,838 |  |  | $576,819,588 | $33,398,545 |
| Hospital discharge^a^ | 3,957 | $72,103 | $4,175 | $285,312,901 | $16,519,959 |
| Hospital discharge^b^ | 1,354 | $204,052 | $11,815 | $276,370,543 | $16,002,185 |
| ER visit^a^ | 5,608 | $1,900 | $110 | $10,654,406 | $616,903 |
| ER visit^b^ | 1,919 | $2,335 | $135 | $4,481,738 | $259,498 |
| Specialty visit^a^ | NA | NA | NA | NA | NA |
| Specialty visit^b^ | NA | NA | NA | NA | NA |
| Follow-up | 12,838 |  |  | $167,568,908 | $9,702,440 |
| Rehabilitation | 12,838 |  |  | $53,846,819 | $3,117,795 |
| Public sector | 9,565 | $3,758 | $218 | $35,943,285 | $2,081,159 |
| Private sector | 3,274 | $5,469 | $317 | $17,903,533 | $1,036,636 |
| Specialty visits | 12,838 |  |  | $18,988,522 | $1,099,458 |
| Public sector | 9,565 | $1,348 | $78 | $12,895,266 | $746,651 |
| Private sector | 3,274 | $1,861 | $108 | $6,093,256 | $352,807 |
| Laboratory tests | 12,838 |  |  | $68,375,418 | $3,959,019 |
| Public sector | 9,565 | $2,337 | $135 | $22,354,305 | $1,294,341 |
| Private sector | 3,274 | $14,057 | $814 | $46,021,113 | $2,664,678 |
| Imaging tests | 12,838 |  |  | $13,752,229 | $796,271 |
| Public sector | 9,565 | $905 | $52 | $8,655,728 | $501,177 |
| Private sector | 3,274 | $1,557 | $90 | $5,096,501 | $295,094 |
| Drugs^c^ | 12,838 |  |  | $12,605,920 | $729,898 |
| Public sector | 9,565 | $546 | $32 | $5,220,013 | $302,245 |
| Private sector | 3,274 | $2,256 | $131 | $7,385,907 | $427,653 |
| **Indirect costs** | **12,838** |  |  | **$15,748,922** | **$911,881** |
| Public sector | 9,565 | $1,227 | $71 | $11,732,947 | $679,352 |
| Private sector | 3,274 | $1,227 | $71 | $4,015,975 | $232,530 |
| **Total costs** | **12,838** |  |  | **$760,137,418** | **$44,012,867** |

FF: fragility fractures; NA: not applicable. Exchange rate: 17.2708 Mexican pesos (MXN) per 1 US Dollar (USD).

^a^Public sector.

^b^Private sector.

^c^Includes pharmacological treatment and supplementation with calcium plus vitamin D.

**Table S35** Projected costs by category of type of fragility fracture

| **Description** | **Major osteoporotic fracture** | | | **All fragility fractures** | | |
| --- | --- | --- | --- | --- | --- | --- |
|  | **FF** | **MXN** | **USD** | **FF** | **MXN** | **USD** |
| **Direct medical costs** | **144,367** | **$10,305,041,190** | **$596,674,224** | **229,239** | **$15,136,580,995** | **$876,426,164** |
| Acute care | 144,367 | $7,972,212,929 | $461,600,674 | 229,239 | $11,723,408,804 | $678,799,407 |
| Hospital discharge^a^ | 38,914 | $3,915,580,066 | $226,716,774 | 62,712 | $5,766,974,044 | $333,914,703 |
| Hospital discharge^b^ | 13,320 | $3,792,856,852 | $219,610,953 | 21,465 | $5,586,223,918 | $323,449,054 |
| ER visit^a^ | 39,362 | $74,787,717 | $4,330,298 | 78,794 | $149,707,719 | $8,668,256 |
| ER visit^b^ | 13,473 | $31,459,185 | $1,821,524 | 26,970 | $62,974,015 | $3,646,271 |
| Specialty visit^a^ | 29,277 | $113,506,919 | $6,572,186 | 29,277 | $113,506,919 | $6,572,186 |
| Specialty visit^b^ | 10,021 | $44,022,190 | $2,548,938 | 10,021 | $44,022,190 | $2,548,938 |
| Follow-up | 144,367 | $2,332,828,261 | $135,073,550 | 229,239 | $3,413,172,191 | $197,626,757 |
| Rehabilitation | 144,367 | $560,219,970 | $32,437,407 | 229,239 | $877,505,581 | $50,808,624 |
| Public sector | 107,553 | $338,332,262 | $19,589,843 | 170,783 | $533,826,389 | $30,909,187 |
| Private sector | 36,814 | $221,887,708 | $12,847,564 | 58,456 | $343,679,193 | $19,899,437 |
| Specialty visits | 144,367 | $341,300,748 | $19,761,722 | 229,239 | $458,671,433 | $26,557,625 |
| Public sector | 107,553 | $229,451,506 | $13,285,517 | 170,783 | $310,854,730 | $17,998,861 |
| Private sector | 36,814 | $111,849,242 | $6,476,205 | 58,456 | $147,816,703 | $8,558,764 |
| Laboratory tests | 144,367 | $968,027,826 | $56,049,970 | 229,239 | $1,454,681,749 | $84,227,815 |
| Public sector | 107,553 | $311,744,893 | $18,050,403 | 170,783 | $469,350,767 | $27,175,971 |
| Private sector | 36,814 | $656,282,932 | $37,999,568 | 58,456 | $985,330,982 | $57,051,844 |
| Imaging tests | 144,367 | $268,778,198 | $15,562,580 | 229,239 | $372,881,988 | $21,590,314 |
| Public sector | 107,553 | $144,455,335 | $8,364,137 | 170,783 | $204,240,566 | $11,825,773 |
| Private sector | 36,814 | $124,322,864 | $7,198,443 | 58,456 | $168,641,423 | $9,764,540 |
| Drugs^c^ | 144,367 | $194,501,518 | $11,261,871 | 229,239 | $249,431,440 | $14,442,379 |
| Public sector | 107,553 | $79,580,346 | $4,607,797 | 170,783 | $102,332,912 | $5,925,198 |
| Private sector | 36,814 | $114,921,172 | $6,654,073 | 58,456 | $147,098,528 | $8,517,181 |
| **Indirect costs** | **144,367** | **$349,704,105** | **$20,248,286** | **229,239** | **$456,630,604** | **$26,439,459** |
| Public sector | 107,553 | $260,529,558 | $15,084,973 | 170,783 | $340,189,800 | $19,697,397 |
| Private sector | 36,814 | $89,174,547 | $5,163,313 | 58,456 | $116,440,804 | $6,742,062 |
| **Total costs** | **144,367** | **$10,654,745,295** | **$616,922,511** | **229,239** | **$15,593,211,598** | **$902,865,623** |

FF: fragility fractures. Exchange rate: 17.2708 Mexican pesos (MXN) per 1 US Dollar (USD).

Major osteoporotic fractures defined as a grouping of the most common fractures comprising hip, spine (clinical vertebral and some major fractures of the lumbar spine and pelvis), proximal humerus and distal forearm fractures.

^a^Public sector.

^b^Private sector.

^c^Includes pharmacological treatment and supplementation with calcium plus vitamin D.

**Table S36** Projected costs by category of health care sector

| **Description** | **Public sector** | | | **Private sector** | | |
| --- | --- | --- | --- | --- | --- | --- |
|  | **FF** | **MXN** | **USD** | **FF** | **MXN** | **USD** |
| **Direct medical costs** | **170,783** | **$7,650,794,043** | **$442,990,136** | **58,456** | **$7,485,786,951** | **$433,436,028** |
| Acute care | 170,783 | $6,030,188,681 | $349,155,145 | 58,456 | $5,693,220,123 | $329,644,262 |
| Hospital discharge | 62,712 | $5,766,974,044 | $333,914,703 | 21,465 | $5,586,223,918 | $323,449,054 |
| ER visit | 78,794 | $149,707,719 | $8,668,256 | 26,970 | $62,974,015 | $3,646,271 |
| Specialty visit | 29,277 | $113,506,919 | $6,572,186 | 10,021 | $44,022,190 | $2,548,938 |
| Follow-up | 170,783 | $1,620,605,362 | $93,834,991 | 58,456 | $1,792,566,828 | $103,791,766 |
| Rehabilitation | 170,783 | $533,826,389 | $30,909,187 | 48,435 | $343,679,193 | $19,899,437 |
| Specialty visits | 170,783 | $310,854,730 | $17,998,861 | 48,435 | $147,816,703 | $8,558,764 |
| Laboratory tests | 170,783 | $469,350,767 | $27,175,971 | 48,435 | $985,330,982 | $57,051,844 |
| Imaging tests | 170,783 | $204,240,566 | $11,825,773 | 48,435 | $168,641,423 | $9,764,540 |
| Drugs^a^ | 170,783 | $102,332,912 | $5,925,198 | 48,435 | $147,098,528 | $8,517,181 |
| **Indirect costs** | **170,783** | **$340,189,800** | **$19,697,397** | **58,456** | **$116,440,804** | **$6,742,062** |
| **Total costs** | **170,783** | **$7,990,983,843** | **$462,687,533** | **58,456** | **$7,602,227,755** | **$440,178,090** |

FF: fragility fractures. Exchange rate: 17.2708 Mexican pesos (MXN) per 1 US Dollar (USD).

^a^Includes pharmacological treatment and supplementation with calcium plus vitamin D.
